# Global and single-cell proteomics view of the co-evolution between neural progenitors and breast cancer cells in a co-culture model

**DOI:** 10.1016/j.ebiom.2024.105325

**Published:** 2024-09-04

**Authors:** Ole Vidhammer Bjørnstad, Manuel Carrasco, Kenneth Finne, Vandana Ardawatia, Ingeborg Winge, Cecilie Askeland, Jarle B. Arnes, Gøril Knutsvik, Dimitrios Kleftogiannis, Joao A. Paulo, Lars A. Akslen, Heidrun Vethe

**Affiliations:** a Centre for Cancer Biomarkers CCBIO, Department of Clinical Medicine, Section for Pathology, University of Bergen, Bergen N-5021, Norway; b Department of Pathology, Haukeland University Hospital, Bergen N-5021, Norway; c Computational Biology Unit (CBU), Department of Informatics, University of Bergen, Bergen N-5021, Norway; d Department of Cell Biology, Harvard Medical School, Boston, MA, USA

**Keywords:** Breast cancer, Molecular subtypes, Cancer innervation, Neural progenitor cells, Imaging mass cytometry, Co-culture model, Proteomics, Doublecortin (DCX)

## Abstract

**Background:**

Presence of nerves in tumours, by axonogenesis and neurogenesis, is gaining increased attention for its impact on cancer initiation and development, and the new field of cancer neuroscience is emerging. A recent study in prostate cancer suggested that the tumour microenvironment may influence cancer progression by recruitment of Doublecortin (DCX)-expressing neural progenitor cells (NPCs). However, the presence of such cells in human breast tumours has not been comprehensively explored.

**Methods:**

Here, we investigate the presence of DCX-expressing cells in breast cancer stromal tissue from patients using Imaging Mass Cytometry. Single-cell analysis of 372,468 cells across histopathological images of 107 breast cancers enabled spatial resolution of neural elements in the stromal compartment in correlation with clinicopathological features of these tumours. In parallel, we established a 3D *in vitro* model mimicking breast cancer neural progenitor-innervation and examined the two cell types as they co-evolved in co-culture by using mass spectrometry-based global proteomics.

**Findings:**

Stromal presence of DCX + cells is associated with tumours of higher histological grade, a basal-like phenotype, and shorter patient survival in tumour tissue from patients with breast cancer. Global proteomics analysis revealed significant changes in the proteomic landscape of both breast cancer cells and neural progenitors in co-culture.

**Interpretation:**

These results support that neural involvement plays an active role in breast cancer and warrants further studies on the relevance of nerve elements for tumour progression.

**Funding:**

This work was supported by the 10.13039/501100005416Research Council of Norway through its Centre of Excellence funding scheme, project number 223250 (to L.A.A), the 10.13039/100008730Norwegian Cancer Society (to L.A.A. and H.V.), the Regional Health Trust Western Norway (Helse Vest) (to L.A.A.), the 10.13039/501100008728Meltzer Research Fund (to H.V.) and the 10.13039/100000002National Institutes of Health (NIH)/10.13039/100000057NIGMS grant R01 GM132129 (to J.A.P.).

**Research in context**

**Evidence before this study**

Investigations into the importance of nerve elements in tumours have revealed its potential impact on cancer development, with studies on prostate cancer indicating that the tumour microenvironment could recruit neural progenitor cells, contributing to cancer progress. However, the presence of such cells in breast tumours has not been thoroughly assessed. Breast cancers with a basal-like or triple-negative phenotype are particularly aggressive with a poor prognosis, emphasizing the necessity for research to explore their biology and discover effective targeted treatments.

**Added value of this study**

Here, we investigate the presence of DCX-expressing stromal cells, assumed to represent neural progenitors in breast cancer tissues, correlating higher stromal DCX + cells with presence of more aggressive cancer features and poorer patient outcome. This cancer cell-neural progenitor cell interaction is further studied using our 3D co-culture model including breast cancer epithelial cells and neural progenitors. This research offers insights into the proteomic changes in both breast cancer cells and neural progenitors following *in vitro* interaction.

**Implications of all the available evidence**

The presence of DCX^+^ neural progenitors in breast cancer stroma, as indicated by this study, might promote aggressive features in these tumours. Further, DCX + stromal cells might have a potential as prognostic biomarkers and therapeutic targets. The findings underscore the need for further research into the role of neural elements in cancer progression, with significant implications for improving patient stratification and treatment approaches.

## Introduction

Nerve involvement in cancer is an expanding topic in current cancer investigations.^1^, ^2^, ^3^ Increased nerve density is associated with tumour aggressiveness in solid tumours, including breast, prostate, gastric, and head and neck cancers.^4^, ^5^, ^6^, ^7^ The interaction between tumour cells and neural elements in the tumour microenvironment (TME) includes perineural invasion, a process in which tumour cells invade nerves, as a route for metastatic spread and a sign of poor prognosis.^8^ It is further suggested that tumours may stimulate their own innervation by activating neural outgrowth from pre-existing nerves in the TME, *i.e.* axonogenesis, similar to the initiation of angiogenesis.^9^^,^^10^

As the normal prostate is considered a highly innervated organ, with anatomical distinct neural input from both sympathetic and parasympathetic peripheral nerves,^11^ tumour innervation is frequently studied in prostate cancer.^4^^,^^12^^,^^13^ However, new findings have indicated that the processes of tumour-nerve crosstalk might also include communication between cancer cells and different neural elements recruited from both the peripheral and central nervous systems.

Recent observations in prostate cancer mouse models have suggested that tumour cells can recruit neural progenitor cells expressing doublecortin (DCX) from the central nervous system (CNS). In this process, termed neo-neurogenesis, *de novo* development of new neurons occurs from NPCs in the TME, giving rise to peripheral nerves of sympathetic character,^5^ presenting a mechanism for how tumours establish their neural elements. Neo-neurogenesis was for long believed to be limited to the subventricular zone near the lateral ventricles and the dentate gyrus of the hippocampus, where new neurons are generated from adult neural stem cells (NSC).^14^^,^^15^ Notably, NPCs found in the TME could potentially also originate from mesenchymal stem cells or other bone marrow-derived cells^16^ or reprogrammed microvascular pericytes.^17^

Although the first evidence of nerve presence in tumours was demonstrated in breast cancer,^18^ the role of nerve involvement in these tumours is an understudied topic. Histological assessments indicate that the normal breast is innervated by sympathetic and sensory nerves,^19^^,^^20^ where sympathetic nerves innervate blood vessels and ducts, while sensory nerves supply the skin including the nipple. In breast cancer, presence of nerve fibres in the TME correlates with poor differentiation, a triple-negative phenotype, lymph node (LN) metastasis, and higher clinical stage.^21^, ^22^, ^23^, ^24^, ^25^ A recent experimental study indicated that triple-negative breast cancers (TNBC) are innervated by sensory nerves, and it was reported that TNBC cells might adhere to sensory nerve fibres from murine dorsal root ganglia in co-culture,^26^ arguing for the use of direct-contact co-culture models to investigate the interaction between nerves and breast cancer cells. Whether breast cancer contain stromal DCX + cells that might contribute to tumour neurogenesis, and how direct-contact co-culture interaction with NPCs would influence breast cancer cells at the functional protein level, remains unknown.

Obtaining CNS-derived neurons from living humans that maintain a differentiation potential for *in vitro* studies is challenging. However, stem cell technology and differentiation of pluripotent stem cells into specific human cell populations represents an important tool for developmental studies, disease modelling and regenerative medicine.^27^^,^^28^ Such technology can be used to generate otherwise quite inaccessible human cell populations for *in vitro* models.

Here, we aim to explore the presence and localization of DCX + cells in primary breast cancer tissue by single-cell spatial proteomics using Imaging Mass Cytometry (IMC). We also generate a 3D *in vitro* model for NPC innervation in breast cancer spheroids to capture mutual interactions and alterations in protein expression by global mass spectrometry proteomics. Our work presents a resource for further studies on cancer-neural crosstalk.

## Methods

### Cohorts of patients with breast cancer

Breast cancers from two independent study cohorts were included in our analysis. *Cohort 1* represents a series of primary invasive breast carcinomas diagnosed in women aged 50–69 as part of the prospective Norwegian Breast Cancer Screening Program in Hordaland County, Norway, during 1996–2003 (n = 534).^29^, ^30^, ^31^ From this cohort, a subgroup of 41 tumour tissues were assessed using available tissue microarrays (TMAs), constructed as previously described.^30^ From these, nine were luminal A, 14 luminal B and 18 triple-negative, basal-like breast cancers. *Cohort 2* is a *BRCA* case–control series established in cooperation with Prof. William D. Foulkes, McGill University, Canada^31^^,^^32^ and collected from patients counselled at the Hereditary Cancer Clinic at McGill University Health Centre (MUHC) and the Jewish General Hospital, Montreal, Canada during 1981–2005. Cohort 2 was supplemented with a selection of confirmed BRCA negative cases from our archive at Haukeland University Hospital and tested by the Department of Genetics, Haukeland University Hospital, as reported.^32^ The project was approved by the Institutional Review Board at McGill University Hospital, A03-M33-02A (Canada), and the Western Regional Committee for Medical and Health Research Ethics, REC West (REK 2014/1984) (Norway). The informed consent was waived by REC West (REK 2014/1984), based on national guidelines. The actual patients included were informed about the research project and the possibility to withdraw. All studies were performed in accordance with guidelines and regulations by the University of Bergen and REK, and in accordance with the Declaration of Helsinki Principles. For Cohort 2, 66 tumour tissues were assessed using available TMAs. Basic clinico-pathological information for both cohorts (n = 107) can be found in Supplementary Table S2. The table includes case ID, molecular subtype, ER, PR and HER2 status, where 0 = negative and 1 = positive (≥10%) for ER and PR, 0 = negative (0,1+,2+) and 1 = positive (3+) for HER2, DAKO Hercep test. Histological grade 1–3, 0 = no lymph node metastases and 1 = lymph node metastasis present.

### Imaging mass cytometry (IMC)

#### Antibody panel

The IMC panel comprised 35 lanthanide (Ln) metal-conjugated antibodies (Supplementary Table S1), in addition to the free metals: iridium which binds double stranded DNA, and ruthenium which binds to tissue components.^33^ Half of the antibodies were purchased pre-conjugated (Fluidigm) including CD45, CD3, CD4, CD8, FoxP3, CKAE1/AE3, and CD31. The remaining antibody clones, herein CD34 and Stathmin, were conjugated in our lab as described below. CD31 and CD34 were conjugated to the same metal isotope.

#### Conjugation of antibodies

The antibodies were conjugated to their respective metal isotope using the Maxpar® X8 antibody labelling kits and protocol (Fluidigm, CA, USA). Five μL of working solution of the metals and 100 ug of the glycerol- and carrier-free antibodies were used for conjugation as described in the protocol. The quantity of the conjugated antibody was determined using a NanoDrop spectrophotometer measuring absorbance of the conjugate at 280 nm. All antibodies were eluted with 20 μL W-buffer (Fluidigm) and diluted to 0.5 mg/mL (or 1:1 for the weakest antibodies) with antibody stabilizer (Candor Biosciences, Wangen, Germany) and stored at 4 °C.

#### Antibody validation

All individual antibodies included in our panel were validated by IHC. Stainings were performed on a test-TMA with positive control tissues such as tonsillar tissue, placenta, hippocampus, cerebellum, autonomic ganglion and peripheral nerve tissue, normal breast tissue, and selected breast carcinomas (ER+/PR+/HER2+; ER-/PR-/HER2-and basal-like tumours). In some cases, IHC staining patterns from the Human Protein Atlas^34^ were also consulted for comparison. As part of the validation process, IMC test stains were then performed on the test-TMA and a pilot-TMA (n = 10) with five luminal-like and five basal-like breast carcinomas.

#### IMC staining protocol

Antibody hybridization was performed according to the “Imaging Mass Cytometry Staining Protocol for FFPE Sectioned Tissue” (Fluidigm) with slight modifications. Freshly cut TMA slides underwent dewaxing, rehydration, and antigen retrieval for 48 min in a Ventana Discovery Ultra Autostainer (Roche Diagnostics GmbH) using CC1-buffer (pH 9). Slides were washed with a soap detergent and rinsed in hot water to remove the oil before they were transferred to a Coplin jar and washed with Maxpar H_2_O and then MaxPar phosphate buffered saline (PBS) (Fluidigm). To avoid non-specific binding, slides were blocked with 3% freshly made bovine serum albumin (BSA)/PBS buffer (Sigma–Aldrich/Merck). The antibody mix containing the individually diluted metal-conjugated antibodies in 0.5% BSA/PBS was applied to the slides, and the slides were stored overnight at 4 °C in a hydration chamber. After antibody incubation, slides were washed first in 0.2% Triton X-100/PBS (Thermo Scientific) and then in Maxpar PBS before being stained with 0.3 μM Iridium (Ir)-intercalator (Fluidigm) for 30 min. Next, slides were washed in Maxpar H_2_O and then incubated with 0.0005% Ruthenium (RuO_4_)/PBS (Electron Microscopy Sciences)^33^ for 3 min. Finally, the slides were briefly washed in Maxpar H_2_O and air-dried.

#### IMC analysis and data pre-processing

Data from 107 breast cancers were acquired by a Helios time-of-flight mass cytometer (CyTOF, RRID:SCR_021055) coupled to a Hyperion Imaging System (Fluidigm, RRID:SCR_023195). The square inscribed in each circular TMA core (diameter 1.0 mm) was laser ablated at 200 Hz at a resolution of approximately 1 μm^2^. Pre-processing of raw data was performed using the CyTOF Software (v7.0.8493; Fluidigm), while the MCD™ Viewer software v1.0.560.6 (Fluidigm, RRID:SCR_023007) was used for visualization of IMC images. The ImcSegmentationPipeline was utilized to process the raw data for downstream analyses.^35^^,^^36^ Using histoCAT (v1.7.6),^37^ the marker intensities and the spatial and morphological features of all images were exported in the csv-file format. To note that despite a well-performed segmentation, nuclei-mismatched signals might occur, especially in cellular areas. Such nuclei-mismatched signals appear when signals from “overlapping cell units” that do not capture the nucleus of an individual cell, are assigned to neighboring cells.

Inspired by the methodology presented by Keren and colleagues,^38^ cell-type annotation was performed in a hierarchical scheme using unsupervised clustering and prior knowledge of cell type defining markers of our antibody panel. The annotated images were visually inspected by expert pathologists and compared with the corresponding H&E images. Areas were inspected for quality within the tissues, like necrosis, scarring, or other tumour features. After this inspection step, Phenograph algorithm (RRID:SCR_016919)^39^ was used to refine the cellular annotation (Supplementary Fig. S1).

#### Downstream analysis of neural markers using IMC

After the single-cell annotation step, we used R language to investigate the intensity level of neural markers DCX, NCAM, INA and Nestin. We deployed a gating strategy based on FlowDensity software,^40^ a supervised clustering algorithm based on density estimation of antibody intensities to define subgroups of cells with relatively high/low expression of DCX, INA, NCAM and Nestin (Supplementary Fig. S1).

### Neural progenitor cell differentiation and passaging

Human embryonic stem cells, H9inGFPhES cells, (passage 37–40, WiCell Research Institute, RRID:CVCL_U163) were cultured in mTeSR1 medium supplemented with 0.5% Penicillin Streptomycin on Matrigel® coated wells. “Monolayer Culture Protocol” from “Generation and Culture of Neural Progenitor Cells Using the STEMdiff Neural System” (STEMCELL Technologies) was used to generate third passage NPC-like cells. Differentiation of Neural Progenitor Cells derived from XCL-1 DCXp-GFP were conducted in accordance with “Protocols for Neural Progenitor Cell Expansion and Dopaminergic Neuron Differentiation” from ATCC.

### Breast cancer cell spheroid generation and maintenance

The breast cancer cell lines MDA-MB-231 (ATCC® HTB-26™, RRID:CVCL_0062), BT549 (ATCC® HTB-122™, RRID:CVCL_1092), HS578T (ATCC® HTB-126™, RRID:CVCL_0332), MCF7 (ATCC® HTB-22™, RRID:CVCL_0031), BT474 (ATCC® HTB-20™, RRID:CVCL_0179), and T47D (ATCC® HTB-133™, RRID:CVCL_0553) were obtained from ATCC. The six basal- and luminal-like cell lines were disassociated with 0.25% trypsin and added individually to wells of a 6 well Ultra-low attachment plate in 2 mL Mammocult culturing media (STEMCELL Technologies). Accutase (STEMCELL Technologies) was further used to disassociate NPC cells. NPCs were added to breast cancer cell containing wells equal to 20% of the total breast cancer cell number. Plates were kept on an orbital shaker (INFORS HT Celltron) at 70 rpm in a 37 °C incubator for the duration of the co-culture. Media was changed every other day. Doxycycline (2 ug/mL) was added to the media to induce GFP expression in the NPCs 48 and 24 h before collection. After 7 days of co-culture, spheroids were collected for processing. All cells used tested negative for mycoplasma contamination using MycoAlert Mycoplasm Detection Kit (Lonza, LT07-318).

### Fluorescence staining and imaging

#### Whole mounting and coverslip

Live spheroids were collected and fixated for 15 min in 10% formalin. To permeabilize the cells, spheroids were put into PBS with 2% Triton 100X for 2 h. Protein blocking was done with 3% BSA for 2 h. The primary antibodies guinea pig polyclonal anti-doublecortin antibody (Merck, AB2253, 1:200 dilution, RRID:AB_1586992), mouse monoclonal anti-CKAE1/AE3 (Dako, M3515, 1:200 dilution, RRID:AB_2132885) were added to their respective spheroids and incubated for 2 days at 4°. The following secondary antibodies were added at 1:500 concentration to their respective primary antibodies: Goat anti-guinea-pig AF 488 (A11073, RRID:AB_2534117), donkey anti-rabbit AF 594 (A21207, RRID:AB_141637) & donkey anti-mouse AF 647 (A31571, RRID:AB_162542). Counterstaining was done with DAPI at a 1:1000 solution for 24 h at 4°. Image acquisition and analysis was conducted with the Andor Dragonfly confocal microscope and Imaris 9.1.3 (Bitplane AG, RRID:SCR_007370). All confocal tile-scan images were merged as a maximum projection.

#### Neural cells

Imaging was conducted using the same protocol principles as for the spheroids. However, neural cells were collected on coverslips before processing. Following the whole mounting protocol, neural cells were stained with rabbit polyclonal anti-tyrosine hydroxylase antibody (Merck, AB152, 1:200 dilution, RRID:AB_390204), guinea pig polyclonal anti-DCX antibody (Merck, AB2253, 1:200 dilution, RRID:AB_1586992), rabbit polyclonal anti-INA antibody (Merck, AB5354, 1:200 dilution, RRID:AB_91800), mouse monoclonal anti-Nestin antibody (CST, 33475, 1:200 dilution, RRID:AB_2799037), rabbit monoclonal anti-Ki-67 antibody (Epredia, RM9106S, 1:200 dilution), chicken polyclonal anti-NFH antibody (Merck, AB5539, 1:200 dilution, RRID:AB_11212161), mouse monoclonal anti-AE1/AE3 antibody (M3515, Dako, 1:200 dilution, RRID:AB_2132885), rabbit polyclonal anti-NFL antibody (AB9568, Merck, 1:200 dilution, RRID:AB_11213875), mouse monoclonal anti-TUBB3 antibody (Santa Cruz, sc-80005, 1:200 dilution, RRID:AB_2210816), rabbit monoclonal anti-SOX1 antibody (Thermo, MA5-32447, 1:200 dilution, RRID:AB_2809724). The following secondary antibodies were utilized for neural cell staining: goat anti-guinea pig secondary antibody AF488 (A11073, RRID:AB_2534117), donkey anti-mouse, secondary antibody AF594 (A21203, RRID:AB_141633), donkey anti-rabbit secondary antibody AF594 (A21207, RRID:AB_141637), goat anti-rabbit secondary antibody AF647 (A27040, RRID:AB_2536101), goat anti-chicken secondary antibody AF647 (A21449, RRID:AB_2535866), donkey anti-mouse secondary antibody AF647 (A31571, RRID:AB_162542). Slides were then mounted and imaged using a Leica TCS SP8 STED 3X confocal microscope.

### Spheroid invasion assay

To measure breast cancer spheroid cell invasion into surrounding extracellular matrix (Matrigel, Corning #356234), we generated spheroids by seeding MDA-MB-231 and MCF7 cells alone (5000 cells per well), or together with NPCs (6000 cells per well, of which 1000 were NPCs), in a ULA round bottom 96-well plate (Corning #7007). Spheroids were allowed to form for 3 days in an incubator at 37 °C, 5% CO_2_. Doxycycline (2 μg/mL) was added to the media for induction of GFP expression in NPCs on day 3. Spheroids were subsequently embedded in Matrigel (4.45 mg/mL) on day 4. IncuCyte® depth of focus brightfield (DF-BF) and green-channel fluorescence images were obtained every 6 h for 7 consecutive days allowing us to quantify invading cells and whole spheroid area. Time course plots of Invading Cell BF Area were generated to show differences in invasive capacity of breast cancer cells in the presence or absence of NPCs.

### Histology and hematoxylin-eosin staining

Spheroids were fixated in 10% formalin for 15 min, washed with PBS twice and resuspended into a 5% agarose in PBS solution. The agarose solution containing spheroids was quickly centrifuged to centre spheroids before the agarose block was cast in paraffin. Sections of 4 μm were cut from the paraffin block and subsequently deparaffinized and rehydrated using xylene and graded ethanol washes, respectively. Hematoxylin and eosin (H&E) staining was then performed using standard procedures.

### Spheroid FACS sorting

Spheroids were dissociated using Accutase (STEMCELL Technologies, 07920), incubated on shaker table for 10 min at 37 °C before being manually pipetted until a single cell suspension remained. Cells were washed with PBS before being transferred to a PBS solution with 5% FBS. 5 min before sorting, cells were stained 1:500 with propidium iodide.

### Proteomic analysis

#### Cell lysis, protein digestion and single-pot, solid-phase-enhanced samples preparation

Cells were washed in PBS and spun down before the cell pellets were resuspended in lysis buffer containing an 8 M Urea, 200 mM EPPS pH8.5 and protease inhibitors (Roche Complete with EDTA (Sigma Aldrich)), homogenized, and sonicated in water bath for 30 s, three times. Protein concentration was determined by a Qubit 3.0 Fluorometer. In total we performed four TMT16-plex experiments (n = 4). After lysis, hESC-derived neural cells were reduced with 100 mM DTT (DiThioThreitol, CAT# 171318-02, Amersham Biosciences) for 1 h at RT, alkylated with 200 mM IAA (Iodoacetamide, CAT# I-6125, Sigma Aldrich) for 1 h at RT (in the dark), followed by digestion of proteins into peptides using Trypsin Porcine (Promega, CAT# V5111) at trypsin-to-protein ratio 1:50 at 37 °C on a shaker. Reaction was quenched with formic acid. Breast cancer cells were processed using Single-pot, solid-phase-enhanced sample preparation (SP3) protocol with Sera-Mag™ SpeedBead Carboxylate-Modified [E3] Magnetic Particles (Thermo Scientific, CAT#65152105050250, CAT#45152105050250) at a 10:1 (wt/wt) bead/protein ratio.^41^ Samples were desalted with Oasis Elution plates. Plates were prepared by centrifuging 500 μL of 70% acetonitrile (ACN)/1% formic acid (FA) at 200 g for 1 min. After, 500 μL 5% ACN/1% FA were centrifuged twice at 200 g for 1 min. Samples were added and centrifuged at 100 g for 3 min. 500 μL 5% ACN/1% FA were added and centrifuged thrice at 200 g for 1 min each. For elution, 100 μL 70% ACN/1% FA were added twice at 100 × *g* for 3 min. Peptides were subsequently concentrated in a SpeedVac.

#### Tandem mass tag (TMT) 16-plex labelling

TMTpro reagents (0.8 mg) were dissolved in anhydrous acetonitrile (40 μL) of which 7 μL was added to the peptides (50 μg) with 13 μL of acetonitrile to achieve a final concentration of approximately 30% (v/v). Following incubation at room temperature for 1 h, the reaction was quenched with hydroxylamine to a final concentration of 0.3% (v/v). TMT-labelled samples were pooled at a 1:1 ratio across all samples. For each experiment, the pooled sample was vacuum centrifuged to near dryness and subjected to C18 solid-phase extraction (SPE) (Sep-Pak, Waters).

#### Off-line basic pH reversed-phase (BPRP) fractionation

We fractionated the pooled, labelled peptide sample using BPRP HPLC^42^ and an Agilent 1200 pump equipped with a degasser and a UV detector (set at 220 and 280 nm wavelength). Peptides were subjected to a 50-min linear gradient from 5% to 35% acetonitrile in 10 mM ammonium bicarbonate pH 8 at a flow rate of 0.6 mL/min over an Agilent 300Extend C18 column (3.5 μm particles, 4.6 mm ID and 220 mm in length). The peptide mixture was fractionated into a total of 96 fractions, which were consolidated into 12 super-fractions,^43^ from which we analyse non-adjacent super-fractions (n = 12). Samples were subsequently acidified with 1% formic acid and vacuum centrifuged to near dryness. Each super-fraction was desalted via StageTip, dried again via vacuum centrifugation, and reconstituted in 5% ACN, 5% FA for LC-MS/MS processing.

#### Liquid chromatography and tandem mass spectrometry

Mass spectrometry data were collected using an Orbitrap Eclipse mass spectrometer (Thermo-Fisher Scientific, San Jose, CA, RRID:SCR_023618) coupled to a Proxeon EASY-nLC 1200 liquid chromatography (LC) pump (ThermoFisher Scientific, San Jose, CA). Peptides were separated on a 100 μm inner diameter microcapillary column packed with ∼40 cm of Accucore150 resin (2.6 μm, 150 Å, ThermoFisher Scientific, San Jose, CA). For each analysis, ∼2 μg was loaded onto the column and separation was achieved using a 90 min gradient of 6–28% acetonitrile in 0.125% formic acid at a flow rate of ∼420 nL/min. For the high-resolution MS2 (hrMS2) method, the scan sequence began with an MS1 spectrum (Orbitrap analysis; resolution, 60,000; mass range, 400−1600 Th; automatic gain control (AGC) target 100%; maximum injection time, auto). All data were acquired with FAIMS using three CVs (−40 V, −60 V, and −80 V) each with a 1 s. TopSpeed method. MS2 analysis consisted of high energy collision-induced dissociation (HCD) with the following settings: resolution, 50000; AGC target, 200%; isolation width, 0.7 Th; normalized collision energy (NCE), 36; maximum injection time, 120 ms.

#### Proteomics data analysis

Mass spectra were processed using a Comet-based software pipeline.^44^^,^^45^ Spectra were converted to mzXML using a modified version of ReAdW.exe. Database searching included all entries from the human UniProt database. This database was concatenated with one composed of all protein sequences in the reversed order. Searches were performed using a 50-ppm precursor ion tolerance for total protein level profiling. TMTpro tags on lysine residues and peptide N termini (+304.207 Da) and carbamidomethylation of cysteine residues (+304.207 Da) were set as static modifications, while oxidation of methionine residues (+15.995 Da) was set as a variable modification. Peptide-spectrum matches (PSMs) were adjusted to a 1% FDR.^46^^,^^47^ PSM filtering was performed using a linear discriminant analysis, as described previously,^48^ while considering the following parameters: XCorr, ΔCn, missed cleavages, peptide length, charge state, and precursor mass accuracy. For TMT-based reporter ion quantitation, we extracted the summed signal-to-noise (S/N) ratio for each TMT channel and found the closest matching centroid to the expected mass of the TMT reporter ion. PSMs were identified, quantified, and collapsed to a 1% peptide false discovery rate (FDR) and then collapsed further to a final protein-level FDR of 1%. Moreover, protein assembly was guided by principles of parsimony to produce the smallest set of proteins necessary to account for all observed peptides. Proteins were quantified by summing reporter ion counts across all matching PSMs, as described previously.^48^ PSMs with poor quality and reporter summed signal-to-noise ratio less than 100, or no MS3 spectra were excluded from quantification.^49^

### Patient survival analyses

The cancer neural interaction proteins (CNIP) signature was scored in the METABRIC Discovery cohort (n = 939; normal-like breast cancer was excluded). Signature scoring was performed as described previously.^50^ In short, the normalized expression value of the gene corresponding to each signature protein were normalized and summed. The signature scores were used to group patients into upper quartile vs. the rest. Survival analyses were performed using the survival R package (v3.6-4). The endpoint was death from breast cancer, and the follow-up time was the time from diagnosis to death or last follow-up. Univariate survival analysis by the Kaplan–Meier method was performed using the log-rank test for differences. Patients who were alive at last date of follow-up or died of other causes were censored.

### Statistics

Correlation plots were made in Agilent GeneSpring GX software (version 14.9, RRID:SCR_010972). Statistically significant proteins were identified by using t-tests between all cancer cell lines compared to co-cultured cancer cell lines (BH adjusted p < 0.05) with baseline transformation being applied to the median of all samples. Subsequent analyses were conducted using unbiased hierarchical cluster analysis (Spearman centered distance metrics with Ward’s drawing rules).

MDS, volcano plots and heatmaps were produced in R language using RStudio (RRID:SCR_000432). For heatmaps, statistically significant proteins were identified by using t-tests between all cancer cell lines compared to co-cultured cancer cell lines (BH FDR, at a corrected p < 0.05). Volcano plots were generated by identifying significant proteins between MCF-7 or MDA-MB-231 and their co-cultured cancer cell line counterparts (BH FDR, at a corrected p < 0.01). Significant proteins were imported into Ingenuity Pathway Analysis program (IPA®, QIAGEN Redwood City, www.qiagen.com/ingenuity, RRID:SCR_008653), working as previously described.^51^ In brief, following settings were used: Expression Fold Change (Exp Fold Change), Relationships to consider (Direct and Indirect Relationships), Reference set (Corresponding data analysis), Interaction networks (70 molecules/network; 25 networks/analysis), Molecule & Canonical Pathway subcategories were determined by “all” data types if not otherwise stated.

### Ethics declaration

The studies have been approved by the Regional Committee for Medical and Health Research Ethics (2014/1984/REK Vest), as well as the work on human embryonic stem cells for generation of different neural cell types (212824/REK Vest).

### Role of funders

The sources of funding did not influence the design of the study, the gathering of data, the analysis of data, the interpretation of results, or the composition of the manuscript.

## Results

### Breast tumours show presence of cells expressing the neural progenitor marker doublecortin

**Fig. 1:**
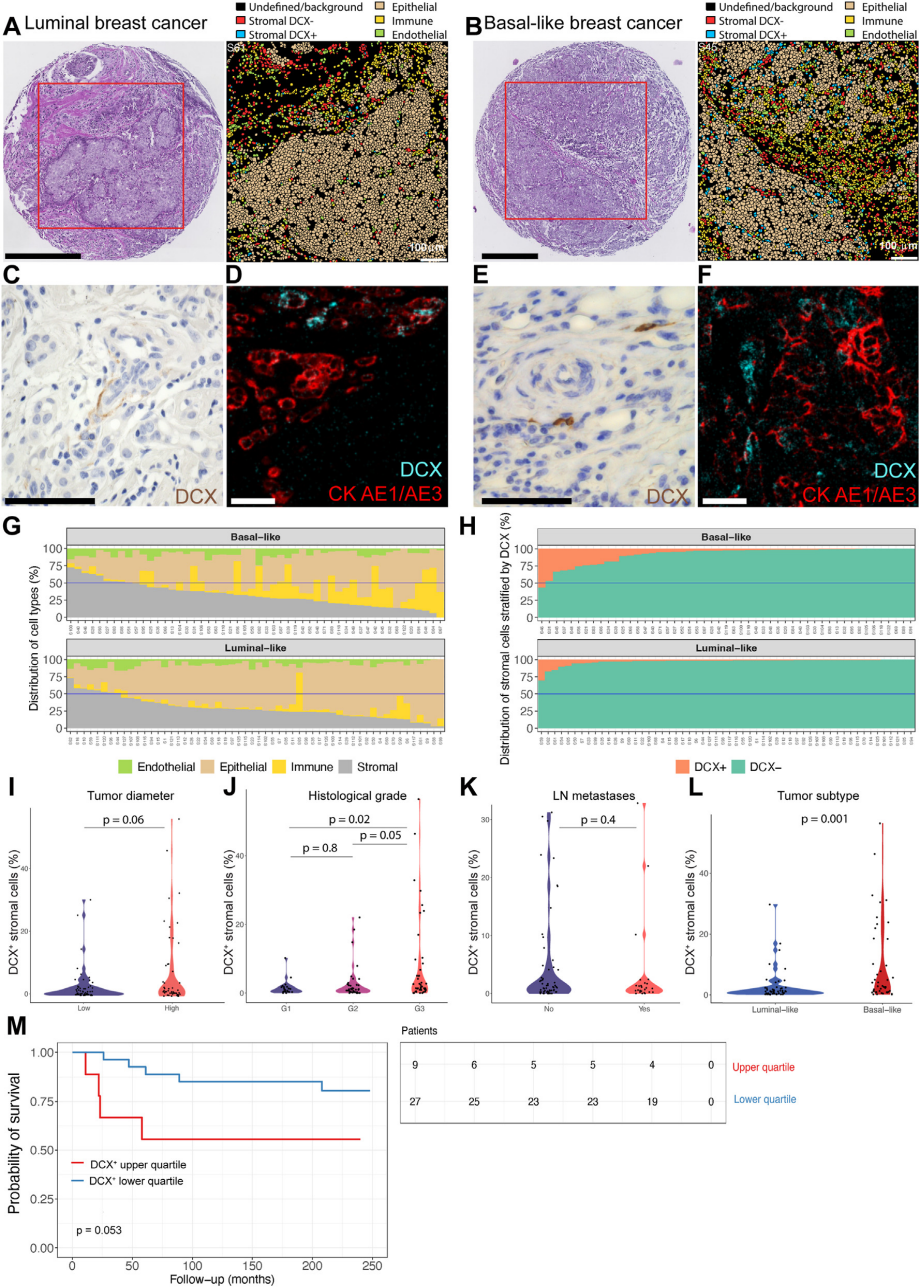
**Stromal expression of doublecortin in breast cancer.** H&E staining of TMA core analysed, imaging segmentation and representative segmentation visualized with AE1/AE3 (epithelial), CD45 (immune), CD31/CD34 (endothelial), DCX-positive and DCX-negative stromal cells, AE1/AE3-negative, vimentin- and αSMA-positive (stromal) of **a** luminal- and **b** basal-like breast cancer tissue. Scale bar: H&E − 300 μm, segmentation plot – 100 μm. **c–f** IHC staining and IMC pseudo image of stromal DCX-positive cells in luminal-like breast cancer (**c and d**) and basal-like breast cancer (**e and f**). Scale bar C, E: 50 μm, D, F: 100 μm **g** Cell compartment distributions in basal- and luminal-like patients with breast cancer. Displaying the endothelial, epithelial, immune, and stromal components of each patient in the selected cohort (n = 107). **h** Subdivision of the stromal cell compartment in basal- and luminal-like breast cancer cases by using DCX to stratify the selected patient cohort. **i** Tumour diameter difference (by Mann–Whitney U test (p = 0.06) by presence of DCX + cell populations in breast cancer. High (>20 mm) and low tumour diameter (≤20 mm). **j** Histological grade (by one-way ANOVA with Tukey multiple comparison test (p = 0.8, 0.02, 0.050) by presence of DCX + cell populations in breast cancer tissue. **k** Lymph node (LN) metastasis by presence of DCX + cell populations in breast cancer tissue (p = 0.46). **l** Proportion of stromal DCX+ in basal-like (number of cells: n = 4696 DCX + cells/n = 55,567 (8.5%) basal stromal cells analysed) and luminal-like (number of cells: n = 2188 DCX + cells/n = 53,974 (4.1%) luminal stromal cells analysed) breast cancer tissue, determined by Mann–Whitney U test (p = 0.001). **m** Kaplan–Meier patient survival plot by content of DCX + stromal cell population log-rank test, p = 0.053). Number of patients at risk is displayed under the plot.

Doublecortin (DCX) is a marker associated with neural progenitor cells and the axonal growth cone of migrating central and peripheral neurons^52^ and has been used to detect neural recruitment processes. Recent findings in mouse models of prostate and breast tumours have demonstrated that DCX + neural progenitors migrate from the CNS to primary and metastatic tumour sites. After migration, these DCX + cells in the TME differentiate to form maturing neurons contributing to tumour progression.^5^ To assess whether human primary breast tumours contain DCX-expressing cells, we used imaging mass cytometry (IMC) analysis to capture DCX-positive cells in the stromal compartment of basal-like and luminal-like breast tumours (Fig. 1a and b). Representative images of stromal DCX-positive cells in both breast cancer subtypes from IHC and IMC analysis are shown Fig. 1 c-f (positive and negative control tissue staining for DCX in Supplementary Fig. S5). We found positive stromal staining for DCX, similar to findings in prostate tumours.^5^

To investigate the population of stromal DCX + cells present in breast tumours in more detail, using our IMC panel of 35 antibodies, we focused on DCX, Nestin, Neural Cell Adhesion Molecule (NCAM) and Internexin-alpha (INA) as representative neural markers (Supplementary Table S1).^53^ First, we evaluated the compartment distributions of cells from the patient cohort (Fig. 1g) and stratified the stromal compartment by DCX (Fig. 1h). Breast cancer stromal tissue showed presence of DCX + cells in a population of 34,244 DCX + cells among 372,468 total single-cells or equally denoted cellular units (9.2% of the total cell population) in tissue from 107 patients with breast cancer, including 56 luminal-like (luminal A n = 26, luminal B n = 30) and 51 with basal-like (TNBC) subtypes (Supplementary Table S2). Further investigations of the DCX + cellular population indicate that they also express other neural progenitor markers including Nestin, NCAM and INA, as shown by higher expression of these markers as compared to all other cells (Supplementary Fig. S1), supporting a neural phenotype of the DCX + cells.

As cancer cells, including prostate cancer cells and migratory glioma cells, have also been shown to express DCX,^54^^,^^55^ we first assessed DCX expression in normal (benign) mammary tissue (n = 3 cases with no concurrent cancer) with IHC staining for DCX, and found no DCX expression in normal mammary epithelium (Supplementary Fig. S5). We also evaluated whole sections of five luminal and five basal-like tumour tissues not included in the cohorts for IMC analysis by IHC staining for DCX, and we could only identify a few cancer cells with weak DCX positivity. Therefore, to avoid any potential contribution of DCX positivity coming from the tumour cells, we focus on stromal DCX expression. To assess the spatial localization of DCX + cells, we first categorized our IMC single-cell data into a tumour cell compartment (marked by AE1/AE3, which recognizes the acidic and basic subfamilies of cytokeratin, making it a stringent marker for epithelial cells) and a stromal compartment (negative for AE1/AE3, positive for vimentin and αSMA) within the luminal-like and basal-like breast cancer subtypes. By analysing the full cohort of 107 cases, we found that the stromal compartment contained ∼ 6.3% DCX + cells (6884 DCX + cells out of 109,541 stomal cells).

### Stromal expression of DCX correlates with tumour aggressiveness in breast cancer

To assess the potential clinical relevance of candidate neural progenitors in breast cancer tissues, stromal DCX + cells (negative for AE1/AE3) were quantified across our cohort of 107 breast tumours (Supplementary Fig. S1). The presence of DCX + cells in the stromal compartment was associated with aggressive tumour features, with higher proportion of DCX + cells in breast tumours with increasing histological grade (Fig. 1j). No significant differences with regards to tumour diameter (Mann–Whitney U test, p = 0.06) and LN metastasis (Mann–Whitney U test, p = 0.4) were found (Fig. 1i and k). When comparing the proportion of stromal DCX + cells in basal-like vs. luminal-like breast cancer tissue, we found higher proportion of DCX + cells in basal-like breast tumours (Fig. 1l; Supplementary Table S3). Higher proportion of DCX + cells (stratification based on 75th quantile) in tumour stroma in our cohort were associated with shorter patient survival (Fig. 1m).

In contrast to the above findings, the presence of DCX + cells in the epithelial compartment showed no significant associations with tumour diameter, histological grade, LN metastasis, or patient survival. Our data indicates that stromal presence of DCX + cells may play a role in breast cancer progression which warrants further investigations into the direct relationship between neural progenitors and breast cancer cells.

### Human pluripotent stem cell differentiation to neural progenitor cells

**Fig. 2:**
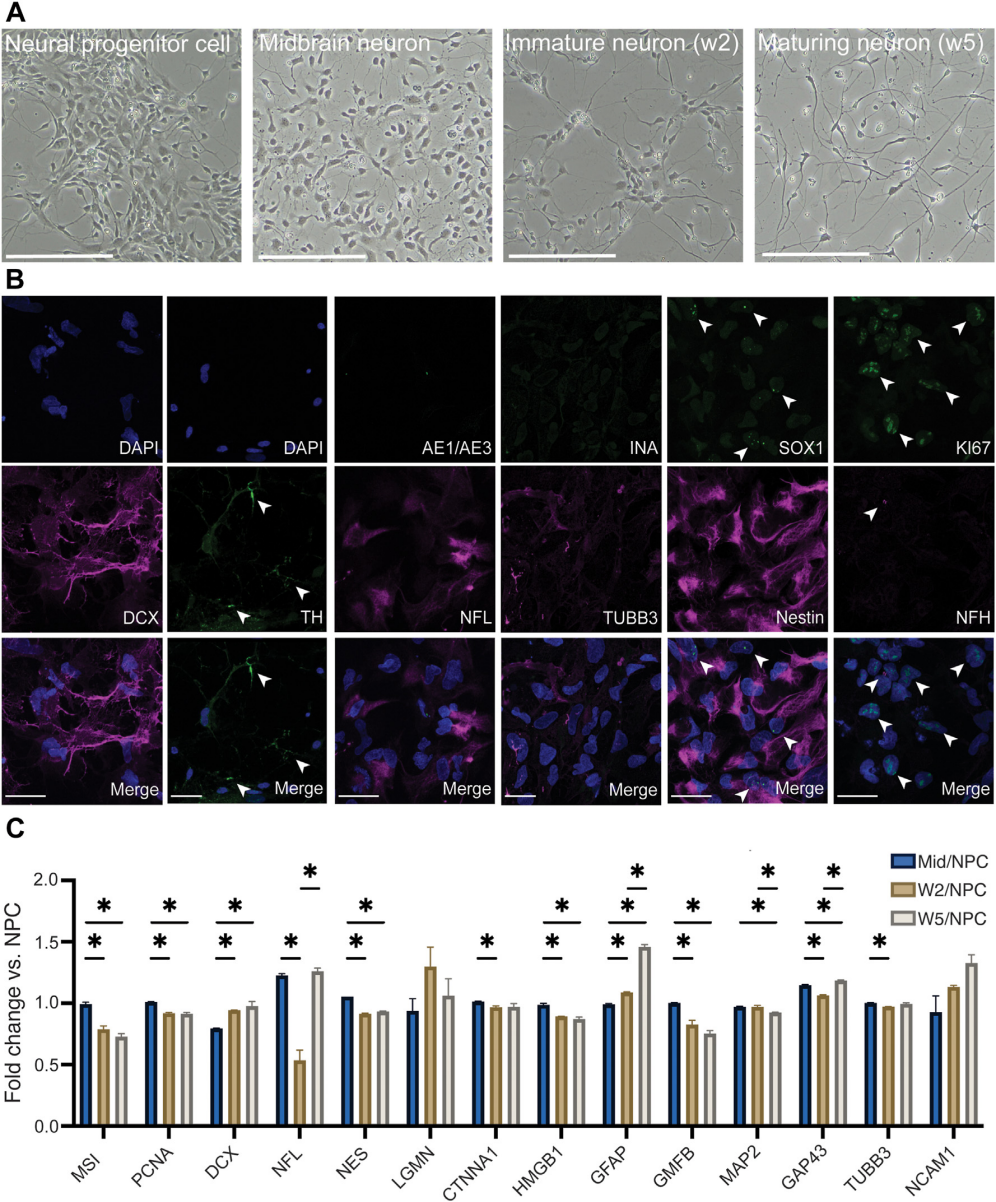
**Neural progenitor cell differentiation. a** Brightfield images displaying the morphological changes in NPCs following differentiation into midbrain dopaminergic neurons, immature neurons (after two weeks of differentiation) and maturing neurons (after five weeks of differentiation). Scale bar – 200 μm. **b** Sp8 confocal images showing presence of DCX, NFL, SOX1, (low) TUBB3, Nestin, KI67 in the hESC-derived NPCs. With staining showing negative expression of INA, NFH and AE1/AE3. Scale bar – 42 μm. Presence of tyrosine hydroxylase (TH) in green. Expression marked by white arrows. **c** Fold change of neural markers comparing midbrain neurons (n = 2 samples), immature neurons (n = 2), and maturing neurons (n = 2 samples at two weeks of maturation and n = 2, 5 weeks) to NPC protein expression SEM bar (∗p ≤ 0.05, significance was determined by unpaired t-test).

To investigate the direct interaction between relevant NPCs from the CNS and breast cancer cells, we generated NPCs from the human embryonic stem cell (hESCs) line H9inGFPhESCs. To test the differentiation potency of NPCs into mature neuron-like cells *in vitro*, we differentiated the H9inGFPhESCs line based on a well-established differentiation protocol generating dopaminergic neurons (Fig. 2a).^56^ Our stem cell-derived NPCs were proliferative (marked by Ki67) and express neural progenitor markers such as: DCX, SOX1, (low) TUBB3 and Nestin, while being negative for mature neural fibre marker NFH, epithelial marker AE1/AE3, and alpha-internexin INA (Fig. 2b). After five weeks of differentiation, our maturing neurons changed morphology (Fig. 2a) and expressed tyrosine hydroxylase (TH) (Fig. 2b).

To characterize the differentiation potential of the NPCs, we assessed the protein expression profiles specific to neural stem cells, neural progenitors, astrocytes and maturing neural cells in hESC-derived NPCs as compared to NPC differentiated cells for up to 5 weeks of *in vitro* maturation, by global proteomics analysis. As expected, the protein profile of the neural stem cell marker (Mushi 1) and proliferation marker (PCNA) decreased during differentiation and subsequent maturation (Fig. 2c). Our hESC-derived NPCs also expressed DCX, the neural growth cone marker neuromodulin (GAP-43),^57^ other neural markers including Nestin, NFL, NCAM and TUBB3, including markers of axonal regeneration (LGMN, CTNNA1, HMGB). We found an increased expression of the astrocyte marker glial fibrillar acidic protein (GFAP) following *in vitro* maturation for up to 5 weeks, whereas the glial maturation factor beta (GMFB) decreased. This suggests a heterogeneous cell population at the end of differentiation containing both neural and glial cells, confirming the multipotent differentiation potential of NPCs.

### Neural progenitor cells interact with breast cancer cells in 3D models *in vitro*

**Fig. 3:**
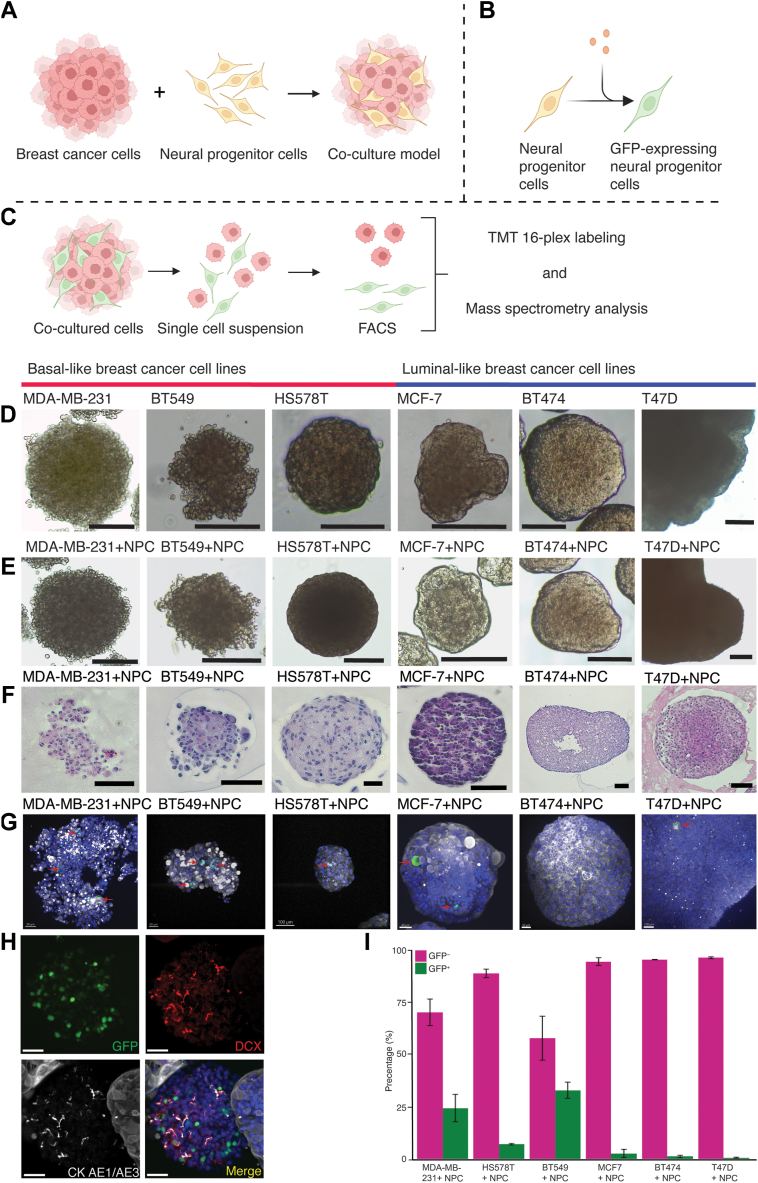
**Model setup of co-cultured breast cancer cell lines and NPCs. a** Schematic presentation of the co-culture model, where breast cancer cells were combined with NPCs. **b** GFP-expression induction by doxycycline in hESCs-derived NPCs. **c** Spheroids containing GFP-expressing NPCs and breast cancer cells were disaggregated into single-cell suspension followed by FACS sorting. Cells negative or positive for GFP were subsequently prepared for mass spectrometry analysis. **d** Brightfield photos of breast cancer spheroids. **e** Brightfield photos of spheroids when combining breast cancer cells and NPCs. **f** H&E staining of the respective BC cell lines as spheroids. **g** High magnification immunofluorescence photos following whole mounted immunofluorescence of cancer-NPC spheroids (DAPI – blue, GFP – green, DCX – red, AE1/AE3 – grey); GFP expressing cells indicated by red arrows. **h** Staining showing spheroids of NPCs in our co-culture model (green – GFP, red – DCX, AE1/AE3 – grey, in merge DAPI – blue,). **i** FACS distribution of GFP positive and negative events during respective sorting of breast cancer cell lines + NPC co-culture (n = 3) SEM bar. Scale bars: d and E = 250 μm, f = 100 μm, g = 50 μm for all but MCF7 = 30 μm and T47D = 100 μm, h = 40 μm. Figure **a–c** created using Biorender.

To model breast cancer innervation, we generated breast cancer spheroids from three basal-like (MDA-MB-231, HS578T, BT549) and three luminal-like (MCF-7, BT474, T47D) breast cancer cell lines, and combined these with H9inGFPhESC-derived NPCs in co-culture (Fig. 3a). Our co-cultures were maintained for seven days; at day six, doxycycline was added to the media for induction of GFP expression in the NPCs (Fig. 3b). After seven days, co-cultured spheroids were collected for whole mounting or cell sorting (Fig. 3c) and subsequent downstream proteomics analysis. The basal-like breast cancer spheroids (MDA-MB-231 and BT549) showed a typical grape-like morphology^58^ (Fig. 3d), where HS578T and the luminal-like breast cancer spheroids (MCF-7, BT474, T47D) showed a mass-like morphology, displaying more compact cell aggregation (Fig. 3d–e, H&E staining of spheroids in Fig. 3f).

When comparing breast cancer spheroids before and after co-culture with NPCs, no obvious morphological differences were observed (Fig. 3d–e). Spheroids varied in diameter, with co-cultures of MDA-MB-231 + NPC, BT549 + NPC and HS578T  +  NPC growing to around 3–500 μm. HS578T  +  NPC co-cultures were more spherical, assuming a circumscribed and firm morphology. MCF-7+NPC and BT474 + NPC formed compact spheroids containing cystic pockets, with an average diameter of 100–250 μm, where BT474 + NPC formed larger spheroids. Smaller T47D + NPC spheroids seem to fuse together and form densely packed spheroids up to 1500 μm in diameter, with no visible cystic areas.

NPCs integrated and proliferated well with MDA-MB-231 and BT549, where the co-cultures contained multiple clusters of NPC cells within the breast cancer spheroids (Fig. 3g). Spheroids containing mostly NPC cells were also produced during the co-culture period (Fig. 3h). In the HS578T + NPC co-culture, there were fewer NPC cells integrated into the HS578T spheroids, with some spheroids containing individual NPCs. The larger number of GFP-positive cells observed in Fig. 3i and Supplementary Fig. S2, might be attributed to NPC cells generating NPC-predominant spheroids in culture. Most luminal-like spheroids seemed to integrate individual cells, but not at similar levels as basal-like breast cancer spheroids, suggesting that basal-like breast cancer cells produce a more favourable microenvironment for NPCs than luminal-like breast cancer cells.

### Global proteome changes in breast cancer cells after interaction with NPCs

**Fig. 4:**
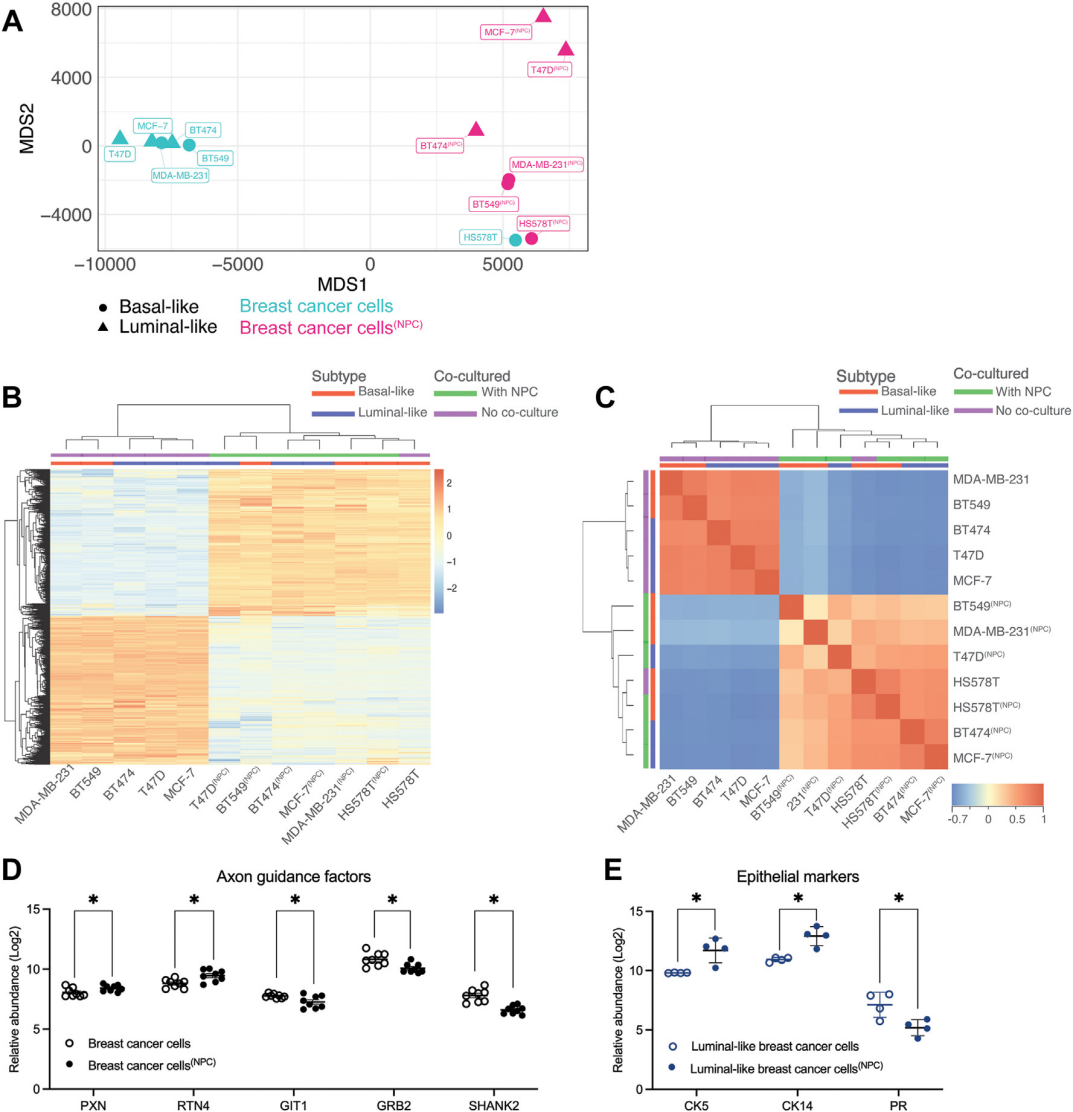
**Proteomic landscape in breast cancer cells before and after NPC co-culture. a** Multidimensional scaling plot displaying the breast cancer cell proteome after the selection of statistically significant proteins before and after co-culture with NPCs (Benjamini Hochberg (BH) adjusted FDR < 0.05), n = 6 breast cancer cell lines. Superscripted text indicates the co-culture conditions. **b** Hierarchical clustering of normalized TMT-ratios pooling breast cancer cells together, except for HS578T, when comparing statistically significant proteins before and after co-culture with NPCs (BH adjusted FDR < 0.05). Colour bar: Blue represents a decreased protein expression compared to the mean intensity with a gradient into beige for no change in expression and into red for proteins with increased expression. **c** Correlation plot of breast cancer cells comparing statistically significant proteins before and after co-culture with NPCs (BH adjusted FDR < 0.05). Each square represents the Spearman correlation coefficient between two samples with comparisons hierarchically clustered. Colour bar: Blue represents a negative correlation with a gradient into beige for no correlation and into red for positive correlation. **d** Selected proteins involved in axon guidance with changed expression pattern before and after co-culture with NPCs (∗p ≤ 0.05, significance was determined by unpaired t-test). **e** Expression of epithelial markers - cytokeratin (CK14, CK5) and progesterone receptor (PR) in luminal-like breast cancer comparing before and after co-culture with NPCs (∗p ≤ 0.05, significance was determined by unpaired t-test).

The interaction between tumour cells and nerves is bidirectional.^4^^,^^59^^,^^60^ Cancer cells can secrete neurotrophic factors, neurotransmitters, and axon guidance factors to stimulate nerve sprouting and infiltration, while nerves can secrete neuroactive factors that can boost tumour growth and spread. To characterize the effect of NPC interaction on the breast cancer cellular proteome, we compared the proteome of FACS-sorted breast cancer cells from the three basal-like (MDA-MB-231, HS578T, BT549) and three luminal-like (MCF-7, BTB474, T47D) spheroid models before and after co-culture with NPCs. Overall, our global proteomics comparison indicated that NPC-interaction changed the expression pattern of 3643 proteins in breast cancer cells (Benjamini Hochberg (BH) adjusted FDR < 0.05). Hierarchical clustering, multidimensional scaling (MDS) and correlation analysis indicated a clear separation of breast cancer cells before and after NPC interaction (Fig. 4a–c), except for HS578T^(NPC)^ which positioned near HS578T, which could indicate that co-culture with NPCs did not change the protein expression pattern of HS578T cells at the same level as for the other breast cancer cell lines studied.

### Global proteomics indicates enhanced expression of proteins involved in aggressive tumour features in breast cancer cells after co-culture with NPCs

With a statistical overrepresentation test using PantherDB,^61^ we found that 10% of the proteins with altered expression in breast cancer cells after co-culture with NPCs, were involved in axon guidance, nervous system development, and signalling via ROBO receptors (n = 232 proteins, FDR 3.92E-23, Fisher’s Exact Test; Supplementary Table S4). To further investigate whether NPCs induced the expression of single proteins involved in driving aggressive tumour features in breast cancer spheroids, we explored the expression of proteins involved in *axon guidance*, *epithelial-to-mesenchymal transition (EMT)*, *migration*, *invasion*, and *proliferation* (Complete list of protein markers in Supplementary Table S5). Axon guidance signalling proteins Paxillin (PXN) and Reticulon4 (RTN4) showed increased abundance, while GIT1, GRB2 and SHRANK2 showed decreased abundance in breast cancer cells after co-culture (Fig. 4d).

**Fig. 5:**
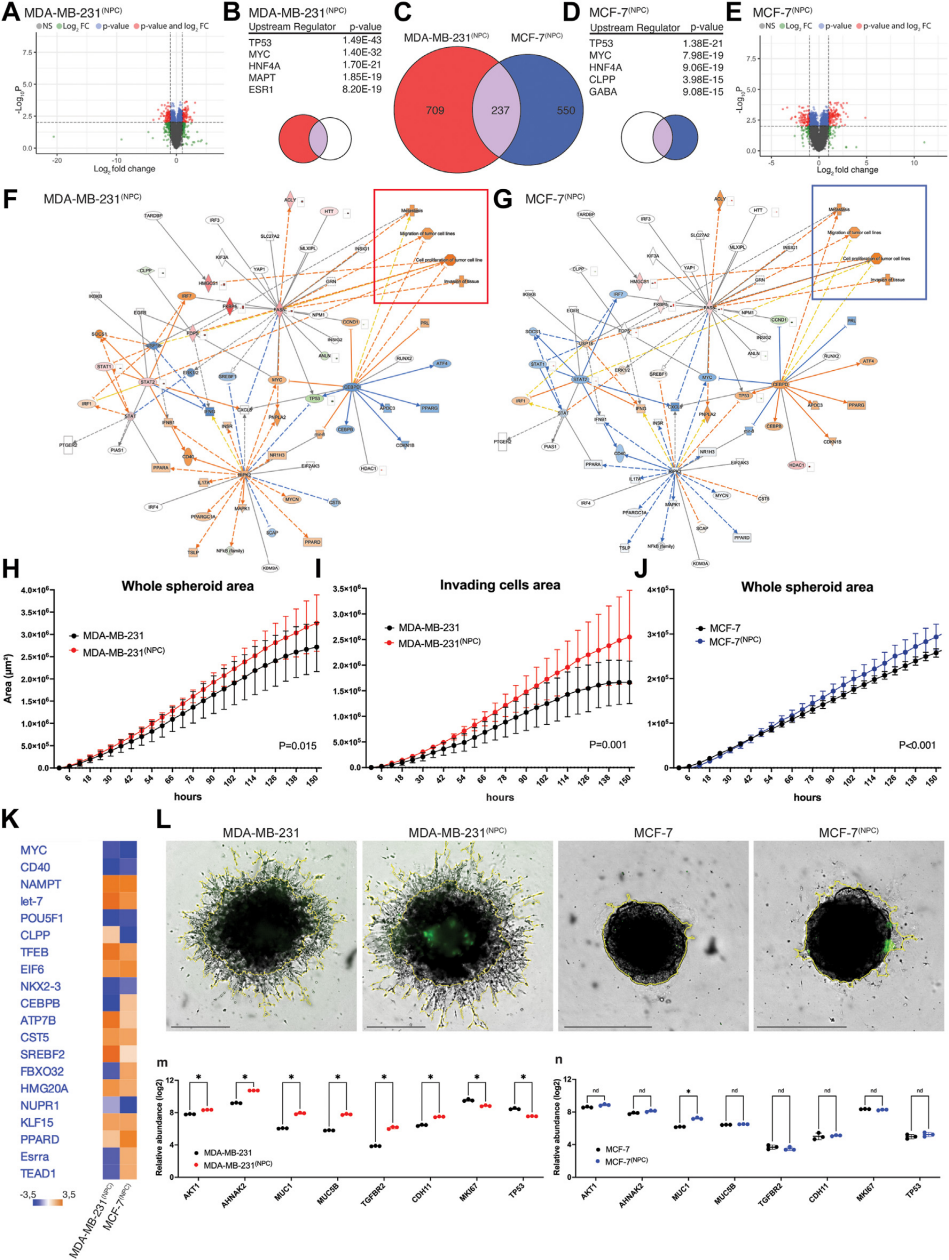
**Differentially expressed proteins (DEPs) in MDA-MB-231 and MCF-7 *after* as compared to *before* co-culture with NPCs. a** Volcano plot representing all DEPs for MDA-MB-231^(NPC)^ relative to MDA-MB-231 (BH adjusted FDR<0.01). **b** IPA generated graphical representation of predicted upstream regulators by DEPs for MDA-MB-231^(NPC)^ with associated p-values (Purple and red, middle and left of Venn diagram). **c** Venn diagram representing the common DEPs between MDA-MB-231^(NPC)^ and MCF-7^(NPC)^, with 237 in common proteins, n = 3 replicated samples for each breast cancer cell line. **d** IPA generated graphical representations of predicted upstream regulators by DEPs for MCF-7^(NPC)^ with associated p-values (Purple and blue, middle, and right of Venn diagram). **e** Volcano plot representing all DEPs for MCF-7^(NPC)^ relative to MCF-7 (BH adjusted FDR <0.01). **f** Selected radial network for the up- and downregulated proteins for the common DEPs in both MDA-MB-231^(NPC)^ and **g** in MCF-7^(NPC)^. Downstream effects are based on pre-existing IPA protein lists. The red frame indicates the predicted activated processes of “*Metastasis*”, “*Migration of tumour cell lines*”, “*Cell proliferation of tumour cell lines*” and “*Invasion of tissue*” for both MDA-MB-231^(NPC)^ and the blue frame shows the same predictions in MCF-7^(NPC)^. Orange – predicted activation, blue – predicted inhibition, red – increased measurement, green – decreased measurement. **h** Tumour spheroid invasion assay: quantification of single spheroid invasion using IncuCyte®. Time course plots show the individual whole spheroid well area and invading area for MDA-MB-231 spheroids, and **j** whole spheroid area for MCF7 spheroids. **i** Brightfield and fluorescence image area showed for MDA-MB-231, MDA-MB-231 co-cultured with NPCs, in MCF7 and MCF7 co-cultured with NPCs (yellow outline mask). All images captured are at 4× magnification. Scale bar 800 μm. Areas are normalized to hour 0. Each point represents mean ± SEM, n = 10 technical replicates for each condition, n = 3 experiments. **k** IPA generated upstream analysis showing predicted upstream regulators based on DEPs calculated as a ratio by comparing protein expression in FACS isolated breast cancer cells after co-culture with NPCs (MDA-MB-231^(NPC)^ and MCF-7^(NPC)^) as compared to breast cancer cells before co-culture. **m** Relative protein expression levels based on quantitative proteomics measurements of single-protein levels of markers involved in EMT, cell migration and proliferation in MDA-MB-231^(NPC)^ cells and **n** MCF-7^(NPC)^ cells (not significant (n.s.) p > 0.05, ∗p ≤ 0.05, significance was determined by unpaired t-test).

We performed an additional TMT 16-plex experiment focusing on one basal-like cell line (MDA-MB-231, n = 3) and one luminal-like cell line (MCF-7, n = 3) before and after co-culture with NPC (Fig. 5), to explore these differences further. When comparing breast cancer spheroids before and after NPC interaction, we found 946 differentially expressed proteins (DEPs) in the basal-like breast cancer MDA-MB-231 spheroids (BH adjusted FDR < 0.01, of which 488 proteins had a FC> ± 1.5) (Fig. 5a–c), while luminal-like MCF-7 spheroids showed 787 DEPs (BH adjusted FDR < 0.01, of which 424 proteins had a FC > ±1.5) (Fig. 5c–e). By analysing the predicted upstream regulators after co-culture, both MDA-MB-231^(NPC)^ and MCF-7s^(NPC)^ top three regulated proteins were TP53, MYC and HNF4A (Fig. 5b–d), with MAPT and ESR1 being the subsequent ranked upstream regulators for MDA-MB-231^(NPC)^, with CLPP and GABA for MCF-7^(NPC)^. Network analysis of the comparative analysis displayed the following key predicted master regulators: RIPK2, CEBPD and FASN (Fig. 5f–g). We found that the expression profile of key regulators predicted activation of processes such as “*cell proliferation of tumour cell lines*”, “*invasion of tissue*”, “*metastasis*” and “*migration of tumour cells lines*” for both MDA-MB-231^(NPC)^ and MCF-7^(NPC)^, suggesting that NPC interactions with our two breast cancer cell lines induced aggressive tumour features. Comparative analysis between MDA-MB-231^(NPC)^ and MCF-7^(NPC)^ breast cancer cell lines following co-culture with NPCs, predicted that the upstream regulators most affected by NPC interaction showed similar alteration levels in both cell lines (Fig. 5f–g). This included stem cell markers such as MYC, POU5F1 (OCT4), LET-7 (Lin28), the microenvironmental stress response marker NUPR1, and the neuronal differentiation marker HMG20A, which together may serve as a panel of candidate markers for further evaluation of neural interaction in other breast cancer cell lines.

### Co-culture with neural progenitor cells increases growth and invasion capacity in MDA-MB-231 breast cancer cells

We further evaluated the effect of co-culture with NPCs on breast cancer cell growth and invasion by measuring breast cancer spheroid extension into surrounding extracellular matrix (Matrigel®) using the IncuCyte Live-Cell Analysis System of 3D Tumour Spheroid Invasion.

The whole spheroid area was significantly larger and the invasive capacity of MDA-MB-231 co-cultured with NPCs was increased as compared to MDA-MB-231 spheroids alone (Fig. 5h–i), suggesting that NPCs drive MDA-MB-231 cells to a more aggressive and invasive phenotype. The integration of NPCs in MDA-MB-231 spheroids was evident though GFP expression, whereas NPCs did not integrate with MCF7 spheroids (Fig. 5l). Instead, NPCs remained on the surface of the MCF7 spheroids, where the NPCs may contribute to increase the overall spheroid area over time (Fig. 5j). As previously reported,^62^^,^^63^ MCF7 spheroids lack sprouts, consistent with their non-invasive nature, which hampers the quantification of invading cell area. Our findings are supported by a previous report showing MDA-MB-231 co-cultured in direct contact with DRG neurons increase migratory speed and invasion of the basal-like cell line.^26^

### Neural progenitors induce a shift in MCF7 breast cancer cells towards a more aggressive basal-like phenotype

To further investigate the predicted aggressive phenotypic alterations observed, we also investigated the expression of specific EMT marker, such as: AKT1, AHNAK2, MUC1 and MUC5B, which were upregulated in both breast cancer cell lines (relative protein abundance levels in Supplementary Tables S6 and S7). In MDA-MB-231^(NPC)^, we detected a significant upregulation of TGFBR2 and CHD11 (Fig. 5h), whereas the expression levels of these two proteins were not significantly changed in MCF-7^(NPC)^ (Fig. 5i). This might reflect the impact of subtype differences between basal- and luminal-like cell lines. The proliferation marker Ki67 was significantly decreased in breast cancer spheroids after co-culture with NPCs, both when comparing MDA-MB-231 and MCF7, and basal-like (MDA-MB-231, BT549, HS578T) combined, but not in the luminal-like cell comparison (MCF7, T47D, BT474). Finally, the top predicted protein TP53 was downregulated in MDA-MB-231^(NPC)^ (Fig. 5h, Supplementary Fig. S6), but not significant in basal-like breast cancer cells (MDA-MB-231, BT549, HS578T) combined. It should be noted that all breast cancer cell line included in this study, except for MCF7, has been annotated with TP53 mutations^64^ and this could influence our results.

As the observed differences in predicted upstream regulators after co-culture with NPCs could be attributed to variations between subtypes, we compared the DEPs between MDA-MB-231 and MCF7 breast cancer cells (1745 proteins, p < 0.05, FC > 1.5) to the DEPs in MCF7 cells co-cultured with NPCs (424 protein, p < 0.05, FC > 1.5) with 182 overlapping proteins While the expected number of DEPs was 144 proteins based on random sampling (Expected 144 (23,8%) Observed 182 (42.9%); Hypergeometric p = 4E-32) (Supplementary Fig. S4). We further asked whether co-culture with NPCs influenced the protein expression of specific cytokeratins (basal cytokeratins CK5, CK14), and progesterone receptor (PR) (Fig. 4e), estrogen receptor and human epidermal growth factor receptor 2 (ERBB2) was not detected in the data set. We found no significant change in expression of basal cytokeratins in basal-like breast cancer cells (MDA-MB-231, BT549, HS578T) after co-culture with NPCs. In contrast, luminal-like breast cancer cells (MCF7, T47D, BT474) showed a fourfold increase in expression of basal cytokeratins CK5 (Student’s t-test, p = 0.03) and CK14 (Student’s t-test, p = 0.02), and a significant reduction in PR expression after co-culture with NPCs (Student’s t-test, p = 0.04). These data could indicate that MCF7 cells shifts towards a more aggressive phenotype like basal-like breast cancer cells.

### Proteomics changes in the co-culture model indicate relevance for human patients

**Fig. 6:**
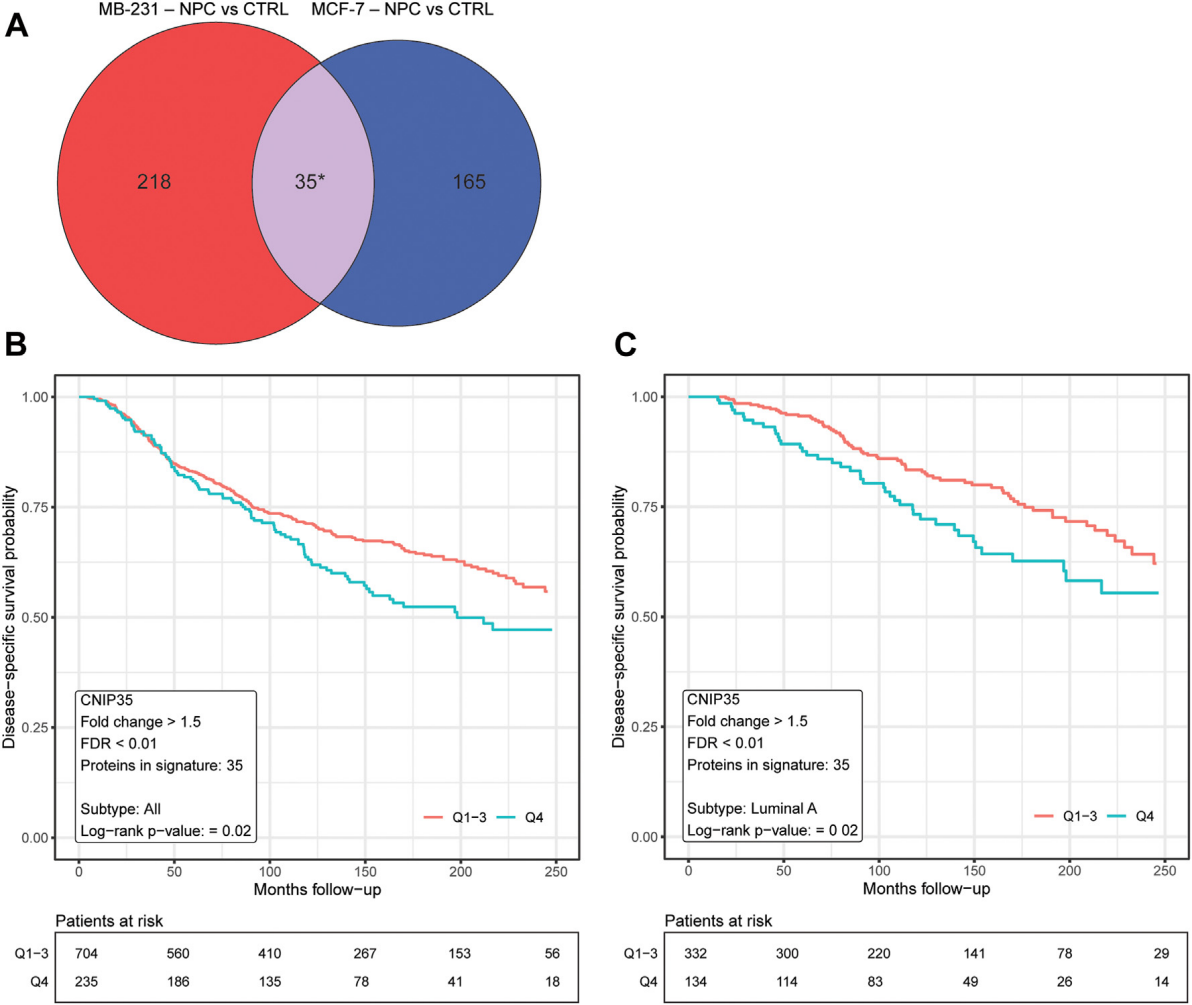
**Univariate survival analysis of consensus protein signature. a** Venn diagram showing the derivation of the consensus protein signature, “Cancer Neural Interaction Protein Signature”, generated by extracting the set of proteins that were significantly upregulated in both MCF7 and MDA-MB-231 breast cancer cells after co-culture with NPC cells (FDR < 0.05, FC > 1.5, Student’s t-test, corrected for multiple testing (Benjamini-Hochberg)). **b** Univariate survival analysis of the consensus signature in the METABRIC Discovery Cohort using Kaplan–Meier method. The upper quartile (blue line) shows reduced probability compared the rest (Q1-Q3; red line) of disease-specific survival for all breast cancer subtypes (p = 0.02, log-rank test) and for **c** luminal A subtype (p = 0.019, log-rank test). Number of patients at risk is displayed under the plot.

To investigate whether the results from these *in vitro* co-cultures were applicable to human patients, we extracted the protein set that was significantly upregulated in both MDA-MB-231 and MCF7 cells after co-culture with NPCs (FDR < 0.05, FC > 1.5, Student’s t-test, corrected for multiple testing (BH)). We termed this set of proteins the “cancer neural interaction proteins (CNIP) signature”, and we interrogated whether this signature score associated with patient survival in the large METABRIC collection of cases with breast cancer, based on corresponding mRNA values. Thus, we found 35 proteins that were upregulated in both cell lines following NPC co-culture and matched with gene entries in the METABRIC Discovery cohort. By scoring each patient based on the expression values of the signature, we performed a univariate survival analysis by the Kaplan–Meier method and found that patients with breast cancer (all subtypes) and with luminal A breast cancer with a high signature score had lower probability of survival (Fig. 6a–c). Combined, these findings support the human relevance of our 3D *in vitro* co-culture cell model, indicating that breast cancer cell lines are affected by the interaction with NPCs towards a more aggressive phenotype.

### Co-culture with breast cancer cells leads to activation of neurogenesis in neural progenitor cells *in vitro*

Neural progenitor cells isolated from prostate tumours in mouse models have previously been shown to induce intratumour neurogenesis and differentiate into mature sympathetic neurons *ex vivo*.^5^ To explore how co-culture with breast cancer cells affected the NPC proteome, we compared sorted NPCs before and after co-culture with breast cancer spheroids and found 235 DEPs (Supplementary Table S8, FC > ±1.5, BH adjusted FDR < 0.05). Network analysis was used to determine potential upstream regulators responsive for driving these proteomics changes in NPCs (Supplementary Fig. S3). Comparing effects of the upstream regulator network we found biological processes such as “*neurogenesis*” and “*development of nervous tissue*” predicted to be activated, while the term “*proliferation of neural cells*” was predicted to be inhibited. Considering the top three canonical pathways affected, remodelling of epithelial adherence junctions was the significantly most changed, with altered BAG2 and FAT10 signalling following, displaying an alteration in cellular adhesion and proteasomal degradation (Supplementary Fig. S3a and b). The top upstream regulators were MAPT, TP53, APP, AYC and PTP4A1. Neural stem and progenitor markers such as MSI1, DCX, NFL, HMGB1 were all significantly decreased after co-culture with breast cancer cells (Supplementary Fig. S3c), suggesting that interaction with breast cancer cells leads to initiation of neural differentiation and maturation of NPCs.

Overall, our data indicate that stromal presence of DCX + cells in breast cancers (n = 107) associates with aggressive tumour features, suggesting that these cells may play a role in breast cancer progression. Our *in vitro* model with NPC integration in breast cancer spheroids allowed us to monitor the mutual interactions between these cell populations in real time and revealed changes in the proteome of breast cancer cells as they co-evolve during interaction with NPCs.

## Discussion

Cancer neuroscience is an emerging research field with discoveries relating to the long-overlooked role of nerves in cancer.^1^^,^^2^ An increasing number of studies have shown nerve fibre involvement in cancer progression.^4^^,^^6^^,^^12^^,^^65^ Most reports have focused on the role of peripheral nerves by axonogenesis, *i.e.* the outgrowth of axons from pre-existing nerves found in the TME.^12^ However, recent observations in prostate cancer indicate a potential new route of cancer-neural cell crosstalk involving CNS-derived neural progenitor cells marked by DCX,^5^ showing a strong correlation between DCX-positive cells and a worse prognosis. In our present study, we explored whether a similar mechanism might occur in breast cancer by investigating presence and localization of DCX + cells in luminal-like and basal-like tumours. We expanded on this by generating a 3D *in vitro* model combining hESC-derived neural progenitor cells and breast cancer spheroids in co-culture to mimic breast cancer innervation.

In prostate cancer, the reported stromal expression of DCX was strongly associated with histological grade and clinical outcome, by using immunofluorescent staining to identify stromal DCX + cells.^5^ However, a more recent report, evaluating DCX expression measured by transcriptome-wide microarray data of whole tissue from radical proctectomy specimens,^66^ did not find significant differences between normal prostate, primary prostate cancer, and metastases, and found no increase with histological grade in a larger patient cohort. Notably, whole tissue analysis could be limited by the heterogenous contributions from both stroma and tumour cells in the samples studied.

To ensure that no potential cancer cell expression of DCX was included in our IMC analysis of breast tumours, we separated stromal from epithelial tissue compartments prior to focusing on the DCX + cell population. Further, we find that stromal DCX + cells also express other markers for neural progenitor cells, in both luminal-like and basal-like tumours, supporting a neural phenotype of these stromal cells. The proportion of stromal DCX + cells were higher among basal-like breast cancers and in tumours with higher histological grade. Also, a trend for decreased patient survival associated with increased DCX + stromal cells were found.

Both local signalling by direct contact between cancer cells and neural elements, or paracrine signalling, as well as systemic effects of circulating neurotrophic factors and neurotransmitters are considered important contributors for tumour neurogenesis.^26^^,^^67^ Further emphasis has been put on the complexity of neural input to tumour progression, as other neural elements including glial cells, such as Schwann cells, also play an important role in establishing a neurogenic niche in the TME.^68^, ^69^, ^70^, ^71^ The emerging field of cancer neuroscience requires further development of marker panels for spatial single-cell analysis of different neural elements contributing to tumour progression alongside the development of experimental models that can elucidate the molecular impact of cancer-neural interactions. Previous studies have used murine neuron-like cell lines, *e.g.* DRG and PC12 cells, to model cancer-nerve crosstalk,^13^^,^^72^ including a recent direct-contact co-culture study of DRG and TNBC cells highlighting a new role of sensory neurons in aggressive breast tumours.^26^ However, experimental *in vitro* models suitable for investigating direct cancer-nerve interactions in a human setting are limited.

Here, we present an experimental co-culture model using hESCs to generate human NPCs *in vitro* that maintained multipotent differentiation potential in culture. During co-culture, we allowed these cells to interacted with breast cancer cells, in direct contact and by paracrine secretion. These interactions triggered significant changes in the proteomic profile both in breast cancer cells and in the NPC population. However, we were unable to reproduce the findings from the PyMT-MMTV breast cancer model, claiming that multipotent NPCs can give rise to mature sympathetic neurons in the TME.^5^ DCX + cells in patient breast cancer tissue did not express TH, nor did we find TH expression in our GFP + cells in the *in vitro* co-culture model following proteomics analysis. This could likely be due to the immaturity of neural progenitor cells, not expressing TH at their cellular state. Still, we did detect TH expression during *in vitro* differentiation of NPCs into maturing dopaminergic neurons at week 5 of differentiation, which suggest that these hESC-derived NPCs have the potential to express TH. Further studies are needed to determine whether NPCs in the breast cancer TME holds the potential to generate mature and functional sympathetic neurons.

The bidirectional interplay between neural progenitors and breast cancer cells is rarely studied. A previous study of MDA-MB-231 and the brain metastatic breast cancer cell line (MDA-MB-231Br) co-cultured with NPCs demonstrated boosted proliferation of MDA-MB-231Br in co-culture, while MDA-MB-231 failed to proliferate, and with NPCs differentiating into astrocytes.^73^ Our proteomics data indicate that a co-culture with NPC promoted aggressive tumour features even in MDA-MB-231, such as induction of proteins involved in cell proliferation, migration, and invasion. By *in vitro* functional invasion assays, we confirmed that NPC interaction with the basal-like cell line MDA-MB-231 increased invasion capacity. At the same time, the proteome of NPCs showed decreased expression of the neural stem cell marker MSI1, neural progenitor marker DCX, and axonal regeneration marker HMGB when co-cultured with breast cancer cells, which may indicate a shift from multipotent neural progenitor cells towards a more mature neural phenotype. Our hESC-derived NPCs were able to integrate with both basal-like (MDA-MB-231, HS578T, BT549) and luminal-like (MCF7, BT474, T47D) breast cancer spheroids, but at varying degrees. Still, our findings appear to suggest a preference for NPC integration in basal-like spheroids, which agree with higher stromal presence of DCX + cells in basal-like breast cancer by IMC analysis, although further studies would be needed to confirm this observation.

Direct-contact cell culture models,^26^ such as ours, hold the potential to characterize yet unidentified factors contributing to drive tumour-nerve crosstalk. The data provided by the co-culture experiments revealed significant changes in the proteomic landscape of cancer cells. We found TP53, MYC and HNF4A, proteins related to cell cycle progression, apoptosis, cellular transformation, genome stability and epithelial-to-mesenchymal transition,^74^ as upstream regulators in breast cancer cells. This is compatible with the expression profile of key regulators predicted by our downstream analysis, indicating increased tumour cell proliferation, invasion and spread. The effect on breast cancer cells by interaction with NPCs points to an induction of aggressive tumour features with increased expression of markers involved in EMT, cell adhesion and cell survival, such as AKT1, AHNAK2 MUC1, and MUC5B.^75^^,^^76^ Cadherin 11 (CDH11) and TGFβ receptor 2 (TGFBR2), showing increased abundance in the basal-like cell line MDA-MB231 after co-culture, but not in the MCF7 luminal-like cell line, might suggest a differential influence of NPC interaction with cancer subtypes. Whether the differences in predicted aggressive tumour behaviour was attributed to subtype differences was further assessed by evaluation of basal cytokeratin expression in luminal-like breast cancer cells after co-culture with NPCs, suggesting enhanced expression of basal-like characteristics by CK14 and CK5 after interaction with NPCs.

Our downstream proteomics analysis generated radial networks displaying potential central upstream master regulators for MDA-MB-231 and MCF7, where the proteins FASN, STAT2, RIPK2 and CEBPD were central nodes. FASN-mediated changes of specific fatty acids has been linked to promote cancer migration in both cell lines, and patients with invasive ductal carcinoma.^77^ STAT2s role in breast cancer is dependent on the specific cancer microenvironment,^78^ which might be reflected in the predicted changes in expression profile in MDA-MB-231 and MCF7 cells. Also, RIPK2 has shown overexpression in tumour tissues, triggering cytotoxic T lymphocyte dysfunction.^79^ Our analysis predicted increased expression of RIPK2 in MDA-MB-231, with no change in MCF7 cells. CEBPD is a transcription factor involved in differentiation and inflammation and is associated with a good prognosis in breast cancer.^80^ The predicted downregulation in MDA-MB-231 and upregulation in MCF7, might also display some of the candidate markers involved in differential interactions between breast cancer subtypes. Thus, NPCs seem to alter differentiation, metabolism, and inflammation responses in co-culture with these two breast cancer cell lines.

Finally, the effect of co-culture on neural progenitors revealed changes in processes such as neurogenesis, development of nervous tissue and proliferation, with downregulation of markers such as MSI1^81^ and DCX,^82^ indicating that these cells are exiting the neural progenitor stage and ceasing a more mature neural differentiation stage, from which proteins such as PA2G4 and HMGB2 seem to play a key role in altering the cellular phenotype of hESC-derived NPCs. PA2G4 encodes for an RNA-binding protein which is involved in growth regulation, and it is associated with neural development by inducing DNA methylation repression.^83^ HMGB2 is a protein highly expressed in adult neural stem cells, where it regulates neural stem cell proliferation and maintenance.^84^ Loss of HMGB2 seems to lead to downstream changes in histone modification, inducing a shift to favour neurogenesis over gliogenesis.^85^

There are certain limitations to this study. Although the causal effect of removing DCX + cells was previously demonstrated by Mauffrey et al. for prostate cancer, by use of transgenic mice with selective genetic depletion of DCX + cells,^5^ this has not been demonstrated in breast cancer. As such, our *in vitro* models and breast cancer tissue spatial proteomics analysis do not demonstrate any causal effect of reduction of DCX + stromal cells in breast cancer and needs to be validated by *in vivo* model experiments. Further, the potential clinical relevance of candidate neural progenitors in breast cancer tissues, would require evaluation of more patient cohorts to dissect the presence of these cells in different breast cancer subtypes. A comprehensive assessment of whether the biological function of NPCs varies in different subtypes of breast cancer also warrants the inclusion of more breast cancer cell lines to assess the role of neural progenitors in breast cancer.

In summary, we identified DCX + cells in the stroma of breast tumours, and the presence of stromal DCX + cells was higher in basal-like BC and tumours with higher histological grade, based on our single-cell analysis. We established a 3D direct-interaction co-culture model mimicking breast cancer NPC-innervation, and proteomics analysis of these co-cultures indicate that NPC interactions induce aggressive tumour features in BC cells. Considering the stromal presence of DCX + cells, we believe that our model may serve as a useful tool for future studies to investigate how neural progenitors and sprouting axons interacts directly with cancer cells and other stromal cells of the breast cancer TME, such as endothelial and immune cells.

## Contributors

H.V. and L.A.A. designed the study; O.V.B. and M.C. performed the experiments, analysed, and interpreted the data, together with H.V. and L.A.A.; K.F., I.W., D.K. and L.A.A. contributed with IMC experiments, data analysis and interpretation; C.A., J.B.A., and G.K. organized the patient cohorts with collection of tissues and patient data, and gathered the tissue microarrays,; J.A.P. performed MS-based proteomics analysis; O.V.B., M.C., D.K., L.A.A. and H.V. wrote the manuscript. The underlying data for the single-cell proteomics by IMC was verified by C.A., K.F., DK., and L.A.A. Underlying data for global proteomics by mass spectrometry analysis was verified by K.F. and H.V. All authors have read, revised, and approved the manuscript.

## Data sharing statement

The mass spectrometry proteomics data have been deposited to the ProteomeXchange Consortium via the PRIDE repository with the dataset identifiers: PXD040662 and PXD044553.

## Declaration of interests

The authors declare no competing interests.

## Appendix ASupplementary data

Supplementary Figs. S1–S6 and Tables S1–S8

BT474 HTB-20_63837200

BT-549 HTB-122_70023401

H9 hESCs original unmodified cells

H9inGFPhESCs.pdf

Hs 578T HTB-126_63271338

MCF7 HTB-22_63288596

MDA-MB-231 HTB-26_70000792

T-47D HTB-133_70036273
