# Supplementary material for: Global and single-cell proteomics view of the co-evolution between neural progenitors and breast cancer cells in a co-culture model
Source: eBioMedicine. 2024 Sep 4;108:105325. doi: 10.1016/j.ebiom.2024.105325 (PMC11404160; doi:10.1016/j.ebiom.2024.105325)
Supplement: Supplementary Figs. S1–S6 and Tables S1–S8 [file mmc1.docx]

**Table of Contents**

[Supplementary Figure 1 2](#_Toc170136078)

[Supplementary Figure 2 3](#_Toc170136079)

[Supplementary Figure 3 4](#_Toc170136080)

[Supplementary Figure 4 5](#_Toc170136081)

[Supplementary Figure 5 6](#_Toc170136082)

[Supplementary Figure 6 7](#_Toc170136083)

[Supplementary Table 1 8](#_Toc170136084)

[Supplementary Table 2 9](#_Toc170136085)

[Supplementary Table 3 12](#_Toc170136086)

[Supplementary Table 4 13](#_Toc170136087)

[Supplementary Table 5 21](#_Toc170136088)

[Supplementary Table 6 22](#_Toc170136089)

[Supplementary Table 7 23](#_Toc170136090)

[Supplementary Table 8 24](#_Toc170136091)

# Supplementary Figure 1


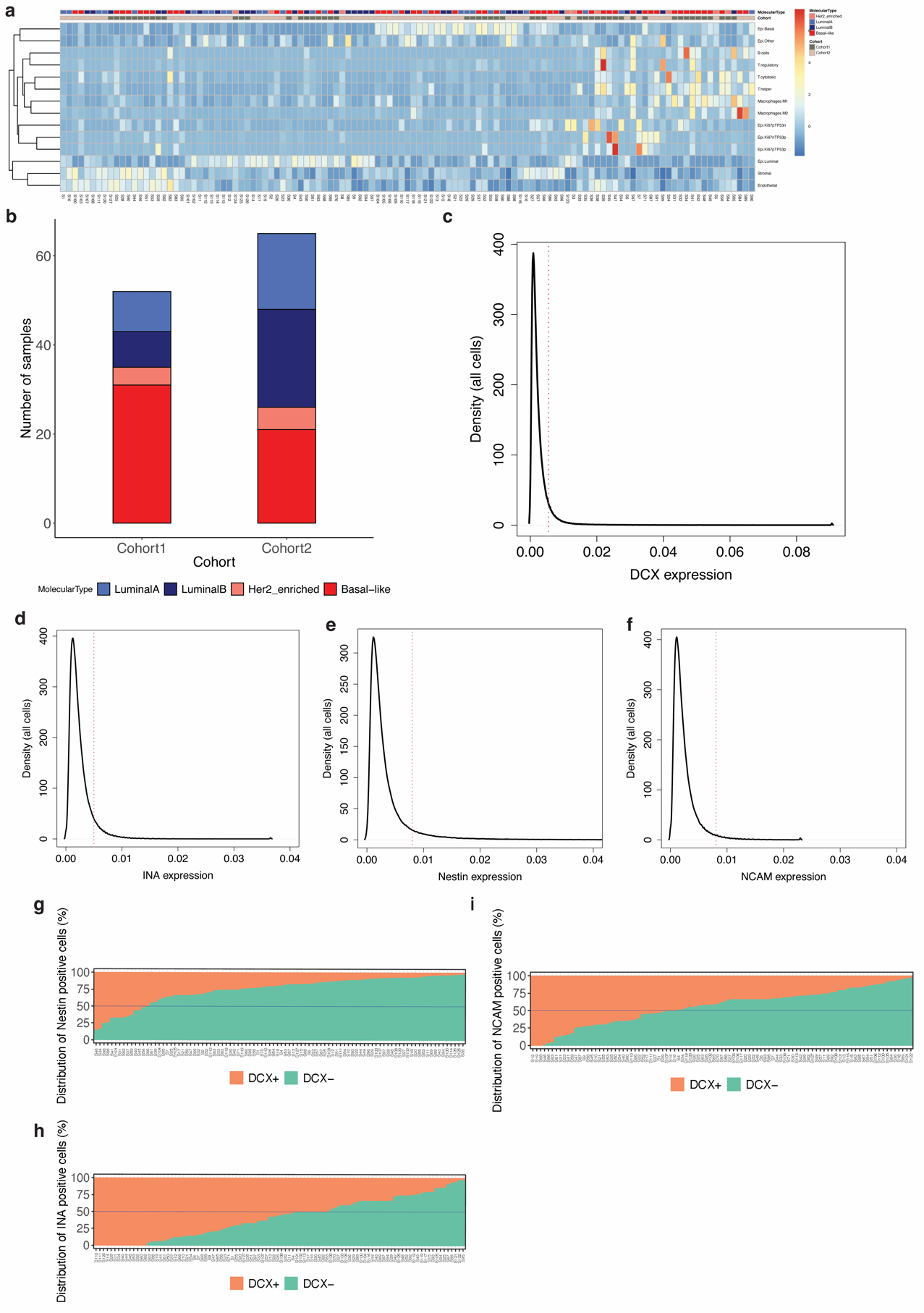


# Supplementary Figure 2


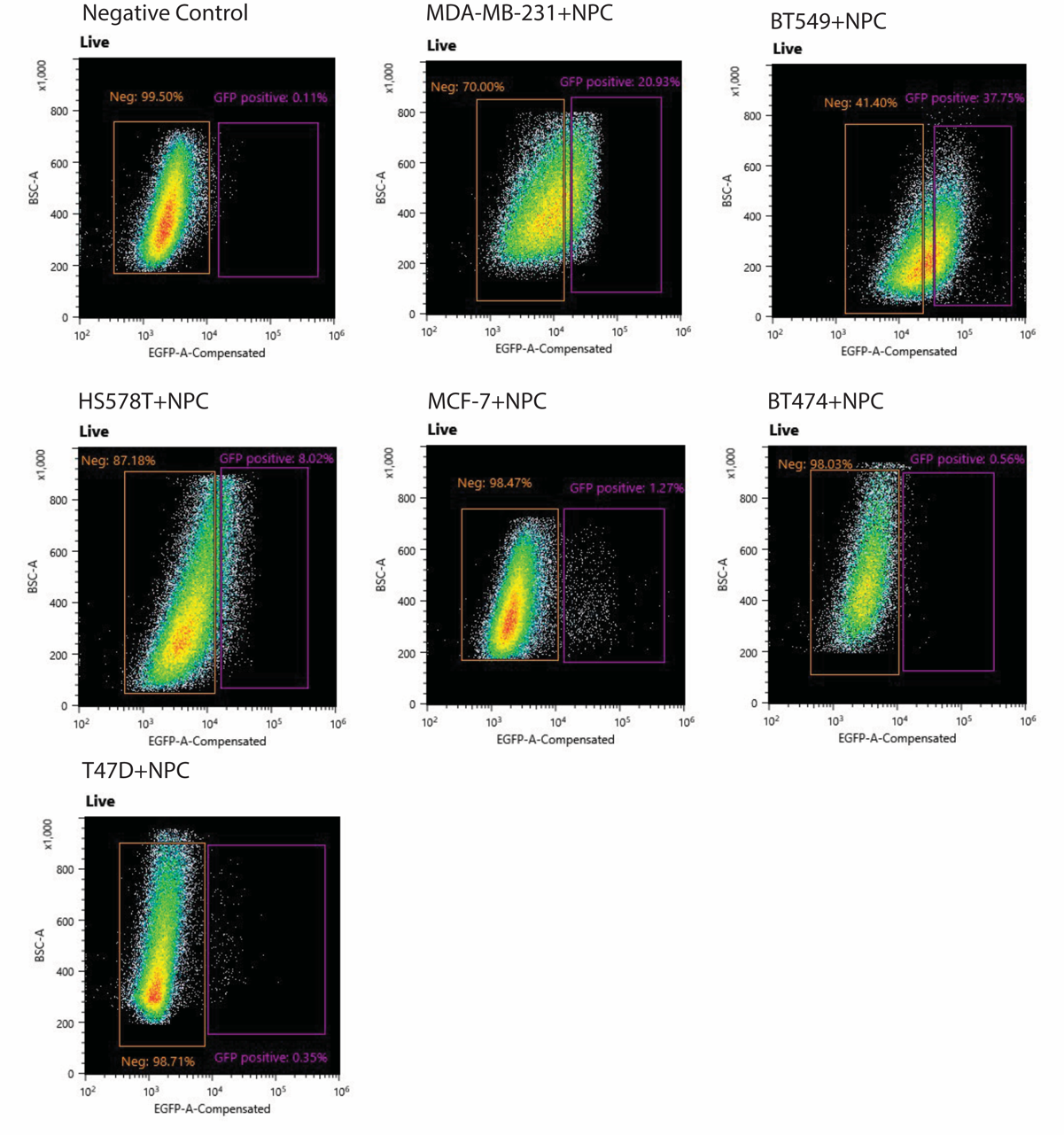


# Supplementary Figure 3


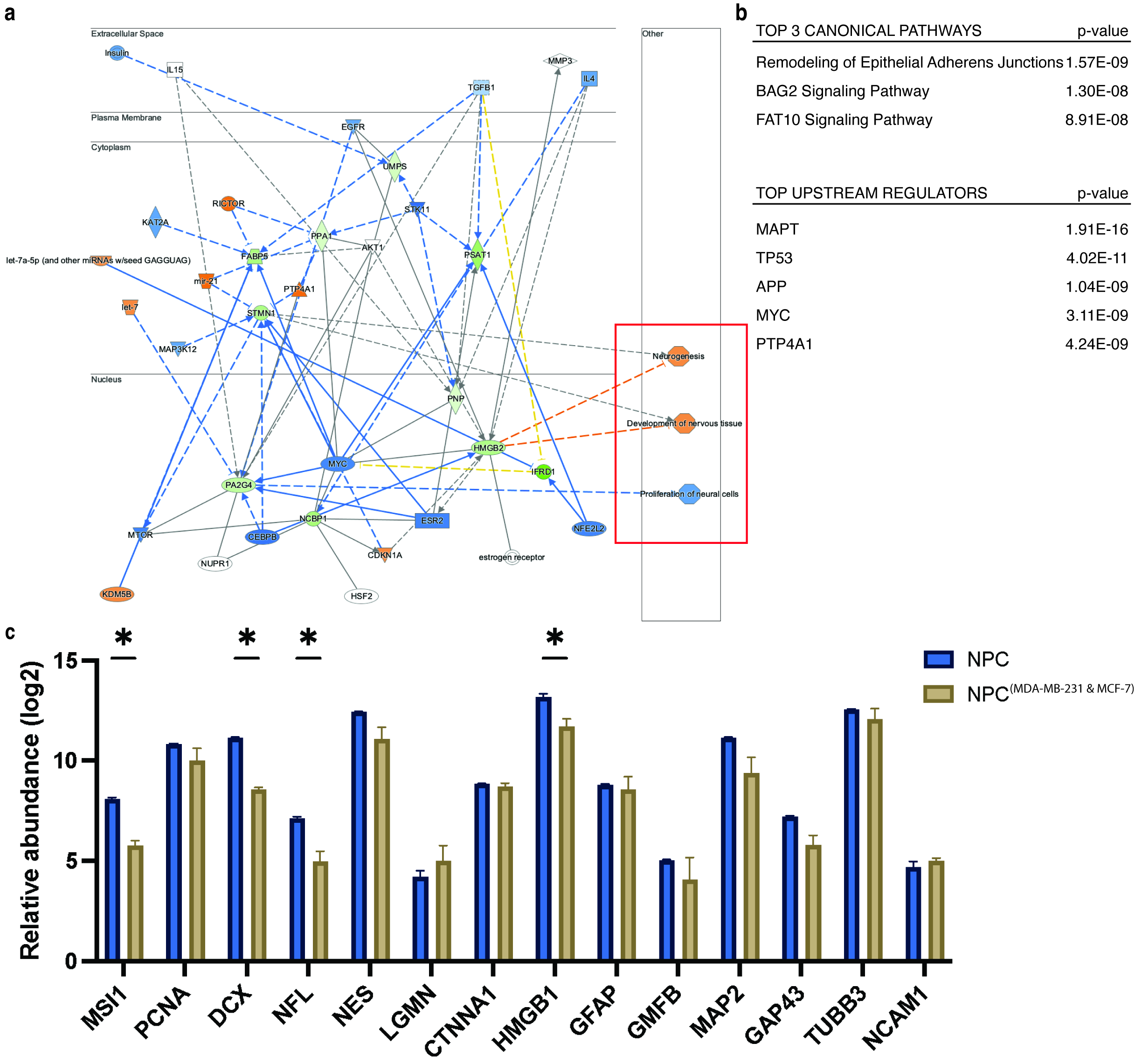


# Supplementary Figure 4

# **Supplementary Figure 5**

# Supplementary Figure 6


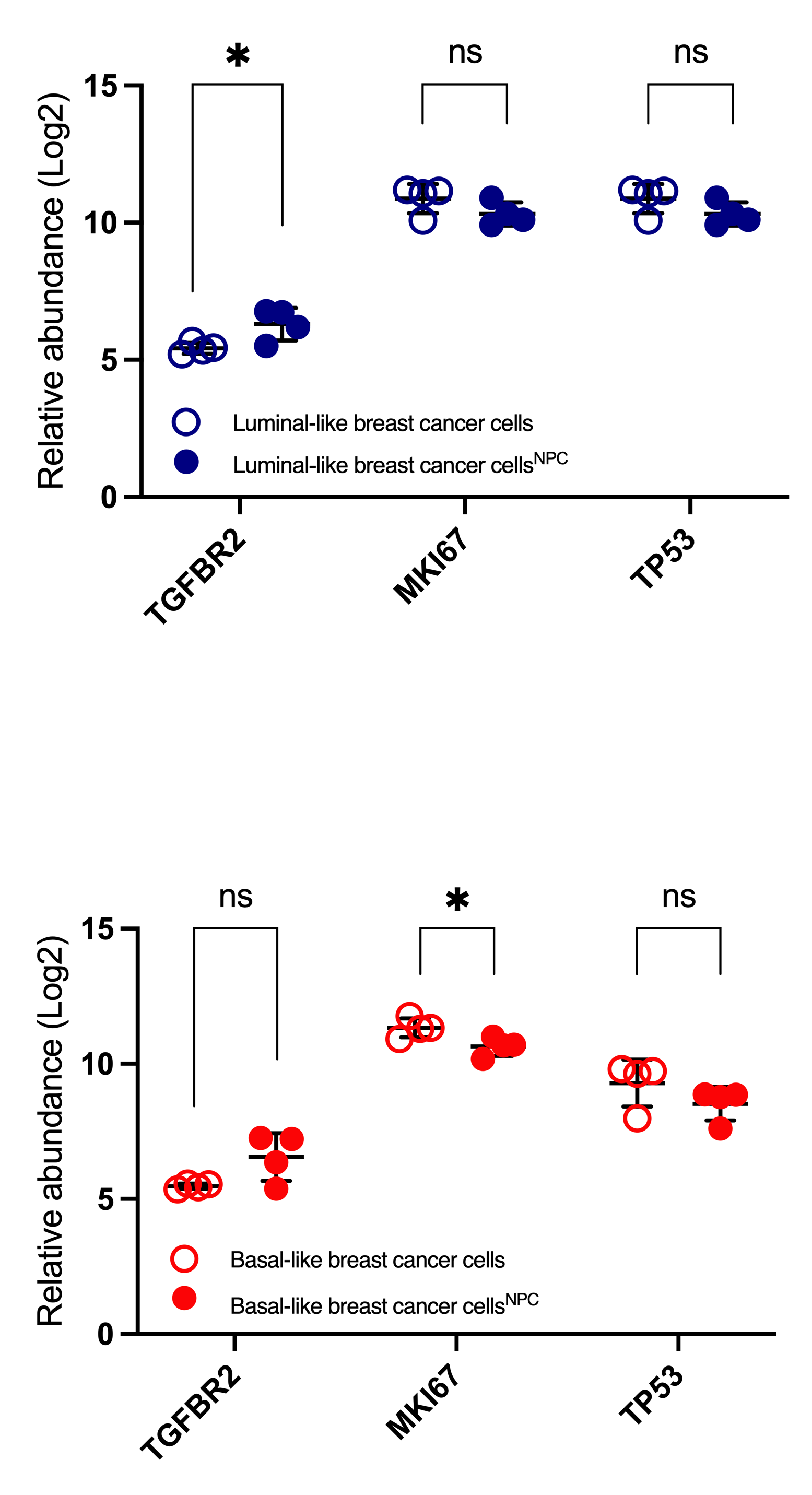


#

# Supplementary Table 1

| **Cell types** | **Antibody** | **Description** | **Vendor, catalog number** | **RRID** |
| --- | --- | --- | --- | --- |
| **Epithelial** | CKAE1/AE3 | Cytokeratin  (Pan CK) | Fluidigm, 3148022D | RRID:AB_2927682 |
|  | CK 5/6 | Basal CK | CST, 71536BF |  |
|  | CK14 | Basal CK | Abcam, ab236439 |  |
|  | CK8/18 | Luminal CK | Fluidigm, 3174022D |  |
| **Stromal** | Alpha-smooth muscle actin (αSMA) | Myofibroblast/  Mesenchymal | Fluidigm, 3141017D | RRID:AB_2890139 |
|  | Vimentin |  | Fluidigm, 3143027D | RRID:AB_3106905 |
| **Endothelial** | CD31 | Endothelial | Fluidigm, 3151025D | RRID:AB_2890140 |
|  | CD34 |  | CST, 3569BF |  |
| **Immune** | CD45 | Leukocyte  (Pan immune) | Fluidigm, 3152018D | RRID:AB_2909538 |
|  | CD4 | T-cell | Fluidigm, 3156033D | RRID:AB_2811051 |
|  | CD8 | T-cell | Fluidigm, 3162034D | RRID:AB_2811053 |
|  | CD3 | T-cell | Fluidigm, 3170019D | RRID:AB_2811048 |
|  | FoxP3 | Regulatory T-cell | CST, 12653BF |  |
|  | CD20 | B-cell | Fluidigm, 3161029D | RRID:AB_2811016 |
|  | CD163 | Macrophage | Fluidigm, 3147021D | RRID:AB_2892115 |
|  | CD68 | Macrophage | Fluidigm, 3159035D | RRID:AB_2810859 |
| **Neural** | Neurofilament-L (NFL) | Neural progenitor | CST, 2837BF |  |
|  | Internexin-alpha (INA) |  | Abcam, ab247282 |  |
|  | Doublecortin (DCX) |  | Abcam, ab222921 |  |
|  | Neural cell adhesion molecule (NCAM) | Neural | CST, 99746BF |  |
|  | Nestin | Neural and endothelial | Biolegend, 656802 | RRID:AB_2562474 |
| **Miscellaneous** | Histone H3 |  | Fluidigm, 3176023D | RRID:AB_2811058 |
|  | PD1  PDL-1  Foxa1  Ki67  Stathmin  PDGFR-beta  CD44  PDPN |  | Fluidigm-3165039D  CST-13684BF  Abcam- ab240935  Fluidigm- 3168022D  CST-13655BF  CST-3169BF  Fluidigm-3153029D  CST-26981BF | RRID:AB_3106909  RRID:AB_2811061  RRID:AB_2890141 |

#

# Supplementary Table 2

| **Case ID** | **Molecular subtype** | **ER** | **PR** | **HER2** | **Tumour diameter (mm)** | **Histological grade** | **Lymph node metastasis** |  |
| --- | --- | --- | --- | --- | --- | --- | --- | --- |
|  |  |  |  |  |  |  |  |  |
| S10 | Luminal A | 1 | 1 | 0 | 10 | 1 |  |  |
| S12 | Luminal A | 1 | 1 | 0 | 15 | 2 | 0 |  |
| S1 | Luminal A | 1 | 1 | 0 | 25 | 1 | 0 |  |
| S26 | Luminal A | 0 | 1 | 0 | 1 | 2 | 1 |  |
| S16 | Luminal A | 1 | 1 | 0 | 30 | 1 | 1 |  |
| S17 | Luminal A | 1 | 1 | 0 | 12 | 2 | 1 |  |
| S19 | Luminal A | 1 | 1 | 0 | 5 | 1 | 1 |  |
| S22 | Luminal A | 1 | 1 | 0 | 10 | 1 | 1 |  |
| S23 | Luminal A | 1 | 1 | 0 | 20 | 1 | 0 |  |
| S29 | Luminal A | 1 | 1 | 0 | 13 | 2 | 0 |  |
| S35 | Luminal A | 1 | 1 | 0 | 8 | 2 | 0 |  |
| S44 | Luminal A | 1 | 1 | 0 | 10 | 1 |  |  |
| S54 | Luminal A | 0 | 1 | 0 | 65 | 1 | 1 |  |
| S55 | Luminal A | 1 | 1 | 0 | 57 | 1 |  |  |
| S60 | Luminal A | 1 | 1 | 0 | 12 | 2 |  |  |
| S61 | Luminal A | 1 | 1 | 0 | 15 | 2 | 0 |  |
| S68 | Luminal A | 1 | 1 | 0 | 9 | 1 | 0 |  |
| S102 | Luminal A | 1 | 1 | 0 | 10 | 1 | 0 |  |
| S90 | Luminal A | 1 | 1 | 0 | 12 | 2 | 0 |  |
| S115 | Luminal A | 1 | 1 | 0 | 8 | 1 | 1 |  |
| S120 | Luminal A | 1 | 1 | 0 | 15 | 2 | 1 |  |
| S121 | Luminal A | 1 | 1 | 0 | 15 | 1 | 0 |  |
| S109 | Luminal A | 1 | 1 | 0 | 20 | 1 | 1 |  |
| S111 | Luminal A | 1 | 1 | 0 | 15 | 1 | 0 |  |
| S112 | Luminal A | 1 | 1 | 0 | 15 | 1 | 0 |  |
| S113 | Luminal A | 1 | 1 | 0 | 7 | 1 | 0 |  |
| S9 | Luminal B | 0 | 1 | 0 | 12 | 2 | 0 |  |
| S7 | Luminal B | 0 | 1 | 0 | 10 | 2 | 0 |  |
| S11 | Luminal B | 1 | 1 | 0 | 10 | 2 |  |  |
| S14 | Luminal B | 1 | 0 | 0 | 30 | 2 | 1 |  |
| S15 | Luminal B | 0 | 1 | 0 | 35 | 3 |  |  |
| S4 | Luminal B | 1 | 1 | 0 | 20 | 3 | 0 |  |
| S6 | Luminal B | 1 | 1 | 0 | 17 | 3 |  |  |
| S18 | Luminal B | 0 | 1 | 0 | 20 | 3 | 1 |  |
| S59 | Luminal B | 0 | 1 | 0 |  | 3 | 0 |  |
| S62 | Luminal B | 1 | 1 | 0 | 30 | 3 |  |  |
| S50 | Luminal B | 1 | 0 | 0 | 8 | 3 |  |  |
| S65 | Luminal B | 0 | 1 | 0 | 8 | 3 |  |  |
| S70 | Luminal B | 1 | 0 | 0 | 15 | 2 |  |  |
| S125 | Luminal B | 1 | 1 | 0 | 20 | 2 | 1 |  |
| S126 | Luminal B | 1 | 0 | 0 | 110 | 3 | 1 |  |
| S127 | Luminal B | 1 | 0 | 0 | 10 | 2 | 0 |  |
| S92 | Luminal B | 1 | 0 | 0 | 16 | 1 | 1 |  |
| S97 | Luminal B | 1 | 1 | 1 | 9 | 1 | 0 |  |
| S101 | Luminal B | 1 | 1 | 0 | 18 | 2 | 1 |  |
| S98 | Luminal B | 1 | 1 | 0 | 40 | 2 | 1 |  |
| S99 | Luminal B | 1 | 1 | 0 | 24 | 3 | 0 |  |
| S85 | Luminal B | 1 | 1 | 0 | 20 | 2 | 0 |  |
| S88 | Luminal B | 1 | 1 | 0 | 30 | 3 | 0 |  |
| S94 | Luminal B | 1 | 1 | 0 | 30 | 3 | 0 |  |
| S96 | Luminal B | 1 | 1 | 0 | 18 | 2 | 0 |  |
| S114 | Luminal B | 1 | 1 | 1 | 25 | 2 | 1 |  |
| S116 | Luminal B | 1 | 1 | 0 | 35 | 2 | 0 |  |
| S117 | Luminal B | 1 | 1 | 0 | 35 | 2 | 0 |  |
| S107 | Luminal B | 1 | 1 | 0 | 12 | 1 | 0 |  |
| S108 | Luminal B | 1 | 1 | 0 | 18 | 1 | 0 |  |
| S25 | Basal-like | 0 | 0 | 0 | 32 | 3 | 0 |  |
| S13 | Basal-like | 0 | 0 | 0 | 30 | 3 | 0 |  |
| S27 | Basal-like | 0 | 0 | 0 | 30 | 2 |  |  |
| S20 | Basal-like | 0 | 0 | 0 | 35 | 2 | 1 |  |
| S21 | Basal-like | 0 | 0 | 0 | 20 | 3 | 0 |  |
| S28 | Basal-like | 0 | 0 | 0 | 15 | 3 |  |  |
| S30 | Basal-like | 0 | 0 | 0 | 13 | 3 | 0 |  |
| S31 | Basal-like | 0 | 0 | 0 | 20 | 3 |  |  |
| S32 | Basal-like | 0 | 0 | 0 | 9 | 3 |  |  |
| S33 | Basal-like | 0 | 0 | 0 | 45 | 3 | 1 |  |
| S34 | Basal-like | 0 | 0 | 0 | 20 |  | 0 |  |
| S36 | Basal-like | 0 | 0 | 0 | 20 | 3 |  |  |
| S41 | Basal-like | 0 | 0 | 0 | 10 |  | 0 |  |
| S42 | Basal-like | 0 | 0 | 0 | 20 | 3 |  |  |
| S43 | Basal-like | 0 | 0 | 0 | 21 | 3 | 0 |  |
| S45 | Basal-like | 0 | 0 | 0 | 30 | 3 | 1 |  |
| S46 | Basal-like | 0 | 0 | 0 | 40 | 3 |  |  |
| S47 | Basal-like | 0 | 0 | 0 | 18 | 3 |  |  |
| S48 | Basal-like | 0 | 0 | 0 | 45 | 3 | 1 |  |
| S49 | Basal-like | 0 | 0 | 0 | 26 | 3 | 0 |  |
| S37 | Basal-like | 0 | 0 | 0 | 21 |  | 0 |  |
| S39 | Basal-like | 0 | 0 | 0 | 40 | 2 | 0 |  |
| S40 | Basal-like | 0 | 0 | 0 | 12 |  | 0 |  |
| S56 | Basal-like | 0 | 0 | 0 | 17 | 3 |  |  |
| S57 | Basal-like | 0 | 0 | 0 |  | 3 |  |  |
| S58 | Basal-like | 0 | 0 | 0 | 32 | 2 | 0 |  |
| S51 | Basal-like | 0 | 0 | 0 | 14 | 3 |  |  |
| S52 | Basal-like | 0 | 0 | 0 | 12 | 2 | 0 |  |
| S53 | Basal-like | 0 | 0 | 0 | 10 | 2 |  |  |
| S66 | Basal-like | 0 | 0 | 0 | 30 | 3 | 0 |  |
| S67 | Basal-like | 0 | 0 | 0 | 55 | 3 | 0 |  |
| S69 | Basal-like | 0 | 0 | 0 | 45 | 2 | 1 |  |
| S71 | Basal-like | 0 | 0 | 0 | 12 | 3 |  |  |
| S100 | Basal-like | 0 | 0 | 0 | 32 | 3 | 0 |  |
| S103 | Basal-like | 0 | 0 | 0 | 23 | 3 | 1 |  |
| S104 | Basal-like | 0 | 0 | 0 | 15 | 2 | 0 |  |
| S82 | Basal-like | 0 | 0 | 0 | 11 | 3 | 0 |  |
| S83 | Basal-like | 0 | 0 | 0 | 20 | 3 | 0 |  |
| S84 | Basal-like | 0 | 0 | 0 | 30 | 2 | 0 |  |
| S86 | Basal-like | 0 | 0 | 0 | 21 | 2 | 1 |  |
| S87 | Basal-like | 0 | 0 | 0 | 25 | 3 | 0 |  |
| S89 | Basal-like | 0 | 0 | 0 | 30 | 3 | 0 |  |
| S91 | Basal-like | 0 | 0 | 0 | 15 | 3 | 0 |  |
| S93 | Basal-like | 0 | 0 | 0 | 15 | 1 | 0 |  |
| S95 | Basal-like | 0 | 0 | 0 | 40 | 2 | 0 |  |
| S118 | Basal-like | 0 | 0 | 0 | 35 | 3 | 0 |  |
| S119 | Basal-like | 0 | 0 | 0 | 39 | 3 | 0 |  |
| S122 | Basal-like | 0 | 0 | 0 | 11 | 3 | 0 |  |
| S105 | Basal-like | 0 | 0 | 0 | 15 | 3 | 1 |  |
| S106 | Basal-like | 0 | 0 | 0 | 7 | 2 | 0 |  |
| S110 | Basal-like | 0 | 0 | 0 | 15 | 2 | 0 |  |

# Supplementary Table 3

| **Molecular subtype** | **Group** | **Number of cells** |
| --- | --- | --- |
| Basal | DCX negative | 50871 |
| Basal | DCX positive | 4696 |
| Luminal | DCX negative | 53974 |
| Luminal | DCX positive | 2188 |

# Supplementary Table 4

| **Reactome pathway** | **Reference list** | **Observed** | **Expected** | **Fold Enrichment** | **P-value** | **FDR** |
| --- | --- | --- | --- | --- | --- | --- |
| Unclassified (UNCLASSIFIED) | 10082 | 1088 | 1816 | 0.6 | 1.92E-111 | 4.78E-108 |
| Metabolism of RNA (R-HSA-8953854) | 661 | 418 | 119 | 3.51 | 7.49E-83 | 9.34E-80 |
| Metabolism of proteins (R-HSA-392499) | 1914 | 679 | 345 | 1.97 | 7.64E-53 | 6.35E-50 |
| Metabolism (R-HSA-1430728) | 2087 | 679 | 376 | 1.81 | 3.75E-42 | 2.34E-39 |
| Translation (R-HSA-72766) | 293 | 193 | 53 | 3.66 | 7.58E-40 | 3.78E-37 |
| Infectious disease (R-HSA-5663205) | 895 | 369 | 161 | 2.29 | 2.69E-38 | 1.12E-35 |
| Disease (R-HSA-1643685) | 1679 | 566 | 302 | 1.87 | 3.86E-38 | 1.38E-35 |
| Cell Cycle (R-HSA-1640170) | 648 | 292 | 117 | 2.5 | 1.27E-35 | 3.96E-33 |
| Cellular responses to stress (R-HSA-2262752) | 718 | 312 | 129 | 2.41 | 1.44E-35 | 3.99E-33 |
| Membrane Trafficking (R-HSA-199991) | 626 | 285 | 113 | 2.53 | 2.21E-35 | 5.50E-33 |
| Processing of Capped Intron-Containing Pre-mRNA (R-HSA-72203) | 238 | 163 | 43 | 3.8 | 3.01E-35 | 6.82E-33 |
| Cellular responses to stimuli (R-HSA-8953897) | 732 | 312 | 132 | 2.37 | 1.62E-34 | 3.38E-32 |
| Cell Cycle, Mitotic (R-HSA-69278) | 517 | 247 | 93 | 2.65 | 3.95E-33 | 7.57E-31 |
| mRNA Splicing - Major Pathway (R-HSA-72163) | 180 | 133 | 32 | 4.1 | 2.73E-31 | 4.86E-29 |
| mRNA Splicing (R-HSA-72172) | 188 | 135 | 34 | 3.99 | 7.44E-31 | 1.24E-28 |
| Vesicle-mediated transport (R-HSA-5653656) | 725 | 296 | 131 | 2.27 | 3.72E-30 | 5.80E-28 |
| Signalling by Rho GTPases (R-HSA-194315) | 670 | 272 | 121 | 2.25 | 1.78E-27 | 2.61E-25 |
| Signalling by Rho GTPases, Miro GTPases and RHOBTB3 (R-HSA-9716542) | 685 | 275 | 123 | 2.23 | 5.11E-27 | 7.07E-25 |
| M Phase (R-HSA-68886) | 375 | 186 | 68 | 2.75 | 1.51E-26 | 1.98E-24 |
| HIV Infection (R-HSA-162906) | 227 | 136 | 41 | 3.33 | 1.83E-25 | 2.29E-23 |
| Axon guidance (R-HSA-422475) | 549 | 232 | 99 | 2.35 | 3.30E-25 | 3.92E-23 |
| Signalling by ROBO receptors (R-HSA-376176) | 215 | 131 | 39 | 3.38 | 5.26E-25 | 5.96E-23 |
| Metabolism of amino acids and derivatives (R-HSA-71291) | 369 | 179 | 66 | 2.69 | 9.41E-25 | 1.02E-22 |
| Nervous system development (R-HSA-9675108) | 574 | 236 | 103 | 2.28 | 1.65E-24 | 1.72E-22 |
| Post-translational protein modification (R-HSA-597592) | 1396 | 437 | 251 | 1.74 | 1.45E-23 | 1.44E-21 |
| rRNA processing (R-HSA-72312) | 202 | 122 | 36 | 3.35 | 3.66E-23 | 3.51E-21 |
| Influenza Infection (R-HSA-168255) | 153 | 104 | 28 | 3.77 | 9.22E-23 | 8.51E-21 |
| Major pathway of rRNA processing in the nucleolus and cytosol (R-HSA-6791226) | 182 | 114 | 33 | 3.48 | 1.05E-22 | 9.37E-21 |
| Mitotic Anaphase (R-HSA-68882) | 227 | 129 | 41 | 3.15 | 1.37E-22 | 1.18E-20 |
| Mitotic Metaphase and Anaphase (R-HSA-2555396) | 228 | 129 | 41 | 3.14 | 1.66E-22 | 1.38E-20 |
| rRNA processing in the nucleus and cytosol (R-HSA-8868773) | 192 | 117 | 35 | 3.38 | 1.94E-22 | 1.56E-20 |
| Regulation of expression of SLITs and ROBOs (R-HSA-9010553) | 169 | 106 | 30 | 3.48 | 3.07E-21 | 2.39E-19 |
| Innate Immune System (R-HSA-168249) | 1107 | 356 | 199 | 1.79 | 9.71E-21 | 7.33E-19 |
| Immune System (R-HSA-168256) | 2055 | 569 | 370 | 1.54 | 1.93E-20 | 1.41E-18 |
| Eukaryotic Translation Initiation (R-HSA-72613) | 119 | 85 | 21 | 3.97 | 7.98E-20 | 5.53E-18 |
| Cap-dependent Translation Initiation (R-HSA-72737) | 119 | 85 | 21 | 3.97 | 7.98E-20 | 5.69E-18 |
| Influenza Viral RNA Transcription and Replication (R-HSA-168273) | 133 | 88 | 24 | 3.67 | 6.17E-19 | 4.16E-17 |
| Neutrophil degranulation (R-HSA-6798695) | 477 | 190 | 86 | 2.21 | 1.08E-18 | 7.11E-17 |
| GTP hydrolysis and joining of the 60S ribosomal subunit (R-HSA-72706) | 112 | 79 | 20 | 3.92 | 2.68E-18 | 1.71E-16 |
| Cell Cycle Checkpoints (R-HSA-69620) | 271 | 131 | 49 | 2.68 | 3.03E-18 | 1.89E-16 |
| GPCR ligand binding (R-HSA-500792) | 463 | 15 | 83 | 0.18 | 3.99E-18 | 2.43E-16 |
| Diseases of signal transduction by growth factor receptors and second messengers (R-HSA-5663202) | 418 | 172 | 75 | 2.28 | 4.10E-18 | 2.44E-16 |
| Programmed Cell Death (R-HSA-5357801) | 202 | 109 | 36 | 3 | 4.60E-18 | 2.67E-16 |
| Signal Transduction (R-HSA-162582) | 2500 | 650 | 450 | 1.44 | 4.81E-18 | 2.72E-16 |
| L13a-mediated translational silencing of Ceruloplasmin expression (R-HSA-156827) | 111 | 78 | 20 | 3.9 | 5.11E-18 | 2.83E-16 |
| Separation of Sister Chromatids (R-HSA-2467813) | 187 | 104 | 34 | 3.09 | 5.66E-18 | 3.07E-16 |
| RHO GTPase Effectors (R-HSA-195258) | 288 | 132 | 52 | 2.54 | 4.30E-17 | 2.28E-15 |
| Host Interactions of HIV factors (R-HSA-162909) | 124 | 80 | 22 | 3.58 | 6.89E-17 | 3.58E-15 |
| G2/M Transition (R-HSA-69275) | 195 | 103 | 35 | 2.93 | 9.91E-17 | 5.04E-15 |
| Apoptosis (R-HSA-109581) | 173 | 96 | 31 | 3.08 | 1.37E-16 | 6.82E-15 |
| Mitotic G2-G2/M phases (R-HSA-453274) | 197 | 103 | 35 | 2.9 | 2.29E-16 | 1.12E-14 |
| Regulation of mRNA stability by proteins that bind AU-rich elements(R-HSA-450531) | 85 | 63 | 15 | 4.11 | 1.33E-15 | 6.37E-14 |
| S Phase (R-HSA-69242) | 161 | 89 | 29 | 3.07 | 2.35E-15 | 1.11E-13 |
| Formation of a pool of free 40S subunits (R-HSA-72689) | 101 | 68 | 18 | 3.74 | 3.13E-15 | 1.45E-13 |
| RHO GTPase cycle (R-HSA-9012999) | 448 | 170 | 81 | 2.11 | 3.39E-15 | 1.54E-13 |
| Nonsense-Mediated Decay (NMD) (R-HSA-927802) | 115 | 72 | 21 | 3.48 | 7.40E-15 | 3.24E-13 |
| Nonsense Mediated Decay (NMD) enhanced by the Exon Junction Complex (EJC) (R-HSA-975957) | 115 | 72 | 21 | 3.48 | 7.40E-15 | 3.29E-13 |
| Selenoamino acid metabolism (R-HSA-2408522) | 116 | 72 | 21 | 3.45 | 1.02E-14 | 4.39E-13 |
| Eukaryotic Translation Elongation (R-HSA-156842) | 94 | 64 | 17 | 3.78 | 1.39E-14 | 5.86E-13 |
| Nonsense Mediated Decay (NMD) independent of the Exon Junction Complex (EJC) (R-HSA-975956) | 95 | 64 | 17 | 3.74 | 1.98E-14 | 8.22E-13 |
| HIV Life Cycle (R-HSA-162587) | 149 | 82 | 27 | 3.05 | 2.35E-14 | 9.62E-13 |
| Gene expression (Transcription) (R-HSA-74160) | 1454 | 402 | 262 | 1.53 | 2.52E-14 | 1.01E-12 |
| Eukaryotic Translation Termination (R-HSA-72764) | 93 | 63 | 17 | 3.76 | 2.63E-14 | 1.04E-12 |
| Developmental Biology (R-HSA-1266738) | 1090 | 320 | 196 | 1.63 | 3.11E-14 | 1.21E-12 |
| Synthesis of DNA (R-HSA-69239) | 119 | 72 | 21 | 3.36 | 4.23E-14 | 1.62E-12 |
| SRP-dependent cotranslational protein targeting to membrane (R-HSA-1799339) | 112 | 68 | 20 | 3.37 | 1.21E-13 | 4.56E-12 |
| Viral mRNA Translation (R-HSA-192823) | 89 | 60 | 16 | 3.74 | 1.25E-13 | 4.59E-12 |
| Peptide chain elongation (R-HSA-156902) | 89 | 60 | 16 | 3.74 | 1.25E-13 | 4.66E-12 |
| Late Phase of HIV Life Cycle (R-HSA-162599) | 136 | 76 | 25 | 3.1 | 1.30E-13 | 4.70E-12 |
| Response of EIF2AK4 (GCN2) to amino acid deficiency (R-HSA-9633012) | 101 | 64 | 18 | 3.52 | 1.53E-13 | 5.44E-12 |
| PTEN Regulation (R-HSA-6807070) | 137 | 76 | 25 | 3.08 | 1.63E-13 | 5.73E-12 |
| Mitotic Prometaphase (R-HSA-68877) | 199 | 95 | 36 | 2.65 | 1.75E-13 | 6.06E-12 |
| Mitotic G1 phase and G1/S transition (R-HSA-453279) | 147 | 79 | 26 | 2.98 | 2.13E-13 | 7.28E-12 |
| Intracellular signalling by second messengers (R-HSA-9006925) | 291 | 121 | 52 | 2.31 | 2.44E-13 | 8.22E-12 |
| Intra-Golgi and retrograde Golgi-to-ER traffic (R-HSA-6811442) | 203 | 96 | 37 | 2.63 | 2.49E-13 | 8.27E-12 |
| G1/S Transition (R-HSA-69206) | 130 | 73 | 23 | 3.12 | 3.63E-13 | 1.19E-11 |
| Selenocysteine synthesis (R-HSA-2408557) | 93 | 59 | 17 | 3.52 | 1.30E-12 | 4.19E-11 |
| ER to Golgi Anterograde Transport (R-HSA-199977) | 155 | 79 | 28 | 2.83 | 1.54E-12 | 4.93E-11 |
| Cellular response to heat stress (R-HSA-3371556) | 88 | 57 | 16 | 3.6 | 1.67E-12 | 5.27E-11 |
| Cellular response to starvation (R-HSA-9711097) | 156 | 79 | 28 | 2.81 | 1.91E-12 | 5.95E-11 |
| Class A/1 (Rhodopsin-like receptors) (R-HSA-373076) | 331 | 12 | 60 | 0.2 | 2.12E-12 | 6.53E-11 |
| Asparagine N-linked glycosylation (R-HSA-446203) | 305 | 121 | 55 | 2.2 | 3.03E-12 | 9.20E-11 |
| PIP3 activates AKT signalling (R-HSA-1257604) | 251 | 105 | 45 | 2.32 | 6.29E-12 | 1.89E-10 |
| Activation of the mRNA upon binding of the cap-binding complex and eIFs, and subsequent binding to 43S (R-HSA-72662) | 59 | 45 | 11 | 4.23 | 7.40E-12 | 2.20E-10 |
| APC/C-mediated degradation of cell cycle proteins (R-HSA-174143) | 87 | 55 | 16 | 3.51 | 8.17E-12 | 2.37E-10 |
| Regulation of mitotic cell cycle (R-HSA-453276) | 87 | 55 | 16 | 3.51 | 8.17E-12 | 2.40E-10 |
| Downstream signalling events of B Cell Receptor (BCR) (R-HSA-1168372) | 79 | 52 | 14 | 3.65 | 1.01E-11 | 2.88E-10 |
| Degradation of beta-catenin by the destruction complex (R-HSA-195253) | 82 | 53 | 15 | 3.59 | 1.06E-11 | 2.99E-10 |
| Translation initiation complex formation (R-HSA-72649) | 58 | 44 | 10 | 4.21 | 1.43E-11 | 3.96E-10 |
| Ribosomal scanning and start codon recognition (R-HSA-72702) | 58 | 44 | 10 | 4.21 | 1.43E-11 | 4.01E-10 |
| G2/M Checkpoints (R-HSA-69481) | 150 | 75 | 27 | 2.78 | 1.70E-11 | 4.66E-10 |
| APC/C:Cdc20 mediated degradation of mitotic proteins (R-HSA-176409) | 75 | 50 | 14 | 3.7 | 1.79E-11 | 4.85E-10 |
| ROS sensing by NFE2L2 (R-HSA-8932339) | 56 | 43 | 10 | 4.26 | 1.84E-11 | 4.94E-10 |
| APC/C:Cdh1 mediated degradation of Cdc20 and other APC/C:Cdh1 targeted proteinsin late mitosis/early G1 (R-HSA-174178) | 73 | 49 | 13 | 3.73 | 2.38E-11 | 6.32E-10 |
| Activation of APC/C and APC/C:Cdc20 mediated degradation of mitotic proteins (R-HSA-176814) | 76 | 50 | 14 | 3.65 | 2.53E-11 | 6.64E-10 |
| Regulation of PTEN stability and activity (R-HSA-8948751) | 68 | 47 | 12 | 3.84 | 2.94E-11 | 7.54E-10 |
| DNA Replication (R-HSA-69306) | 159 | 77 | 29 | 2.69 | 2.93E-11 | 7.61E-10 |
| CLEC7A (Dectin-1) signalling (R-HSA-5607764) | 96 | 57 | 17 | 3.3 | 3.07E-11 | 7.82E-10 |
| Regulation of APC/C activators between G1/S and early anaphase (R-HSA-176408) | 80 | 51 | 14 | 3.54 | 3.71E-11 | 9.33E-10 |
| Activation of NF-kappaB in B cells (R-HSA-1169091) | 66 | 46 | 12 | 3.87 | 3.88E-11 | 9.67E-10 |
| Cdc20:Phospho-APC/C mediated degradation of Cyclin A (R-HSA-174184) | 72 | 48 | 13 | 3.7 | 4.50E-11 | 1.11E-09 |
| AUF1 (hnRNP D0) binds and destabilizes mRNA (R-HSA-450408) | 53 | 41 | 10 | 4.29 | 4.59E-11 | 1.12E-09 |
| Regulation of HMOX1 expression and activity (R-HSA-9707587) | 64 | 45 | 12 | 3.9 | 5.12E-11 | 1.24E-09 |
| Switching of origins to a post-replicative state (R-HSA-69052) | 90 | 54 | 16 | 3.33 | 5.41E-11 | 1.30E-09 |
| COPI-mediated anterograde transport (R-HSA-6807878) | 102 | 58 | 18 | 3.16 | 5.51E-11 | 1.31E-09 |
| APC/C:Cdc20 mediated degradation of Securin (R-HSA-174154) | 67 | 46 | 12 | 3.81 | 5.58E-11 | 1.31E-09 |
| Dectin-1 mediated noncanonical NF-kB signalling (R-HSA-5607761) | 59 | 43 | 11 | 4.05 | 6.04E-11 | 1.41E-09 |
| APC:Cdc20 mediated degradation of cell cycle proteins prior to satisfaction of the cell cycle checkpoint (R-HSA-179419) | 73 | 48 | 13 | 3.65 | 6.37E-11 | 1.47E-09 |
| Transcriptional Regulation by TP53 (R-HSA-3700989) | 360 | 130 | 65 | 2 | 8.76E-11 | 2.00E-09 |
| Negative regulation of NOTCH4 signalling (R-HSA-9604323) | 52 | 40 | 9 | 4.27 | 8.88E-11 | 2.01E-09 |
| Signalling by Receptor Tyrosine Kinases (R-HSA-9006934) | 505 | 166 | 91 | 1.82 | 9.28E-11 | 2.08E-09 |
| SARS-CoV Infections (R-HSA-9679506) | 154 | 74 | 28 | 2.67 | 9.47E-11 | 2.11E-09 |
| Autodegradation of Cdh1 by Cdh1:APC/C (R-HSA-174084) | 63 | 44 | 11 | 3.88 | 9.76E-11 | 2.15E-09 |
| Glucose metabolism (R-HSA-70326) | 86 | 52 | 15 | 3.36 | 9.92E-11 | 2.17E-09 |
| NIK-->noncanonical NF-kB signalling (R-HSA-5676590) | 58 | 42 | 10 | 4.02 | 1.16E-10 | 2.51E-09 |
| CDK-mediated phosphorylation and removal of Cdc6 (R-HSA-69017) | 72 | 47 | 13 | 3.62 | 1.20E-10 | 2.57E-09 |
| The role of GTSE1 in G2/M progression after G2 checkpoint (R-HSA-8852276) | 78 | 49 | 14 | 3.49 | 1.29E-10 | 2.76E-09 |
| Regulation of RUNX3 expression and activity (R-HSA-8941858) | 53 | 40 | 10 | 4.19 | 1.33E-10 | 2.81E-09 |
| Orc1 removal from chromatin (R-HSA-68949) | 70 | 46 | 13 | 3.65 | 1.60E-10 | 3.36E-09 |
| Ubiquitin-dependent degradation of Cyclin D (R-HSA-75815) | 51 | 39 | 9 | 4.24 | 1.72E-10 | 3.48E-09 |
| Degradation of GLI2 by the proteasome (R-HSA-5610783) | 59 | 42 | 11 | 3.95 | 1.69E-10 | 3.49E-09 |
| Autodegradation of the E3 ubiquitin ligase COP1 (R-HSA-349425) | 51 | 39 | 9 | 4.24 | 1.72E-10 | 3.51E-09 |
| GLI3 is processed to GLI3R by the proteasome (R-HSA-5610785) | 59 | 42 | 11 | 3.95 | 1.69E-10 | 3.52E-09 |
| Transport to the Golgi and subsequent modification (R-HSA-948021) | 186 | 82 | 34 | 2.45 | 1.89E-10 | 3.79E-09 |
| Nucleotide Excision Repair (R-HSA-5696398) | 110 | 59 | 20 | 2.98 | 2.23E-10 | 4.41E-09 |
| Regulation of activated PAK-2p34 by proteasome mediated degradation (R-HSA-211733) | 49 | 38 | 9 | 4.3 | 2.21E-10 | 4.41E-09 |
| Transport of Mature Transcript to Cytoplasm (R-HSA-72202) | 80 | 49 | 14 | 3.4 | 2.46E-10 | 4.82E-09 |
| Regulation of Apoptosis (R-HSA-169911) | 52 | 39 | 9 | 4.16 | 2.57E-10 | 5.00E-09 |
| Stabilization of p53 (R-HSA-69541) | 55 | 40 | 10 | 4.04 | 2.91E-10 | 5.62E-09 |
| RNA Polymerase II Transcription (R-HSA-73857) | 1319 | 347 | 238 | 1.46 | 2.99E-10 | 5.74E-09 |
| p53-Dependent G1 DNA Damage Response (R-HSA-69563) | 64 | 43 | 12 | 3.73 | 3.80E-10 | 7.19E-09 |
| p53-Dependent G1/S DNA damage checkpoint (R-HSA-69580) | 64 | 43 | 12 | 3.73 | 3.80E-10 | 7.24E-09 |
| Degradation of DVL (R-HSA-4641258) | 56 | 40 | 10 | 3.97 | 4.26E-10 | 7.98E-09 |
| Degradation of GLI1 by the proteasome (R-HSA-5610780) | 59 | 41 | 11 | 3.86 | 4.68E-10 | 8.52E-09 |
| Mitochondrial translation (R-HSA-5368287) | 94 | 53 | 17 | 3.13 | 4.61E-10 | 8.52E-09 |
| Cyclin E associated events during G1/S transition (R-HSA-69202) | 82 | 49 | 15 | 3.32 | 4.58E-10 | 8.52E-09 |
| SCF(Skp2)-mediated degradation of p27/p21 (R-HSA-187577) | 59 | 41 | 11 | 3.86 | 4.68E-10 | 8.58E-09 |
| Ubiquitin Mediated Degradation of Phosphorylated Cdc25A (R-HSA-69601) | 51 | 38 | 9 | 4.14 | 4.95E-10 | 8.75E-09 |
| p53-Independent DNA Damage Response (R-HSA-69610) | 51 | 38 | 9 | 4.14 | 4.95E-10 | 8.81E-09 |
| p53-Independent G1/S DNA damage checkpoint (R-HSA-69613) | 51 | 38 | 9 | 4.14 | 4.95E-10 | 8.87E-09 |
| Vpu mediated degradation of CD4 (R-HSA-180534) | 51 | 38 | 9 | 4.14 | 4.95E-10 | 8.94E-09 |
| Mitochondrial translation elongation (R-HSA-5389840) | 88 | 51 | 16 | 3.22 | 5.47E-10 | 9.60E-09 |
| Cytoprotection by HMOX1 (R-HSA-9707564) | 120 | 61 | 22 | 2.82 | 5.60E-10 | 9.69E-09 |
| FBXL7 down-regulates AURKA during mitotic entry and in early mitosis (R-HSA-8854050) | 54 | 39 | 10 | 4.01 | 5.58E-10 | 9.74E-09 |
| tRNA processing (R-HSA-72306) | 104 | 56 | 19 | 2.99 | 6.04E-10 | 1.03E-08 |
| Interleukin-1 signalling (R-HSA-9020702) | 100 | 55 | 18 | 3.05 | 6.00E-10 | 1.03E-08 |
| Defective CFTR causes cystic fibrosis (R-HSA-5678895) | 60 | 41 | 11 | 3.79 | 6.72E-10 | 1.14E-08 |
| G1/S DNA Damage Checkpoints (R-HSA-69615) | 66 | 43 | 12 | 3.62 | 7.60E-10 | 1.28E-08 |
| Hh mutants are degraded by ERAD (R-HSA-5362768) | 55 | 39 | 10 | 3.94 | 8.14E-10 | 1.36E-08 |
| C-type lectin receptors (CLRs) (R-HSA-5621481) | 138 | 66 | 25 | 2.65 | 8.75E-10 | 1.45E-08 |
| tRNA Aminoacylation (R-HSA-379724) | 42 | 34 | 8 | 4.49 | 8.95E-10 | 1.48E-08 |
| Cellular response to chemical stress (R-HSA-9711123) | 154 | 71 | 28 | 2.56 | 9.62E-10 | 1.58E-08 |
| Class I MHC mediated antigen processing & presentation (R-HSA-983169) | 378 | 130 | 68 | 1.91 | 1.04E-09 | 1.69E-08 |
| Cyclin A:Cdk2-associated events at S phase entry (R-HSA-69656) | 84 | 49 | 15 | 3.24 | 1.09E-09 | 1.77E-08 |
| PCP/CE pathway (R-HSA-4086400) | 91 | 51 | 16 | 3.11 | 1.14E-09 | 1.83E-08 |
| Deadenylation-dependent mRNA decay (R-HSA-429914) | 56 | 39 | 10 | 3.87 | 1.18E-09 | 1.88E-08 |
| Formation of the ternary complex, and subsequently, the 43S complex(R-HSA-72695) | 51 | 37 | 9 | 4.03 | 1.40E-09 | 2.23E-08 |
| Regulation of HSF1-mediated heat shock response (R-HSA-3371453) | 68 | 43 | 12 | 3.51 | 1.48E-09 | 2.34E-08 |
| SCF-beta-TrCP mediated degradation of Emi1 (R-HSA-174113) | 54 | 38 | 10 | 3.91 | 1.56E-09 | 2.44E-08 |
| Metabolism of polyamines (R-HSA-351202) | 57 | 39 | 10 | 3.8 | 1.69E-09 | 2.64E-08 |
| Cytokine Signalling in Immune system (R-HSA-1280215) | 697 | 205 | 126 | 1.63 | 1.71E-09 | 2.64E-08 |
| Cross-presentation of soluble exogenous antigens (endosomes) (R-HSA-1236978) | 49 | 36 | 9 | 4.08 | 1.83E-09 | 2.82E-08 |
| Golgi-to-ER retrograde transport (R-HSA-8856688) | 135 | 64 | 24 | 2.63 | 2.02E-09 | 3.09E-08 |
| Vif-mediated degradation of APOBEC3G (R-HSA-180585) | 52 | 37 | 9 | 3.95 | 2.05E-09 | 3.12E-08 |
| SUMOylation (R-HSA-2990846) | 163 | 72 | 29 | 2.45 | 2.42E-09 | 3.60E-08 |
| Hh mutants abrogate ligand secretion (R-HSA-5387390) | 58 | 39 | 10 | 3.73 | 2.42E-09 | 3.61E-08 |
| CDT1 association with the CDC6:ORC:origin complex (R-HSA-68827) | 58 | 39 | 10 | 3.73 | 2.42E-09 | 3.63E-08 |
| RHO GTPases Activate Formins (R-HSA-5663220) | 136 | 64 | 25 | 2.61 | 2.41E-09 | 3.63E-08 |
| SUMO E3 ligases SUMOylate target proteins (R-HSA-3108232) | 157 | 70 | 28 | 2.48 | 2.57E-09 | 3.79E-08 |
| Regulation of ornithine decarboxylase (ODC) (R-HSA-350562) | 50 | 36 | 9 | 4 | 2.69E-09 | 3.95E-08 |
| Mitochondrial translation initiation (R-HSA-5368286) | 88 | 49 | 16 | 3.09 | 2.80E-09 | 4.06E-08 |
| Mitochondrial translation termination (R-HSA-5419276) | 88 | 49 | 16 | 3.09 | 2.80E-09 | 4.08E-08 |
| DNA Repair (R-HSA-73894) | 310 | 111 | 56 | 1.99 | 2.85E-09 | 4.11E-08 |
| DNA Replication Pre-Initiation (R-HSA-69002) | 116 | 58 | 21 | 2.78 | 3.71E-09 | 5.31E-08 |
| Degradation of AXIN (R-HSA-4641257) | 54 | 37 | 10 | 3.8 | 4.27E-09 | 6.09E-08 |
| Interleukin-1 family signalling (R-HSA-446652) | 138 | 64 | 25 | 2.57 | 4.86E-09 | 6.89E-08 |
| trans-Golgi Network Vesicle Budding (R-HSA-199992) | 72 | 43 | 13 | 3.32 | 5.27E-09 | 7.43E-08 |
| Resolution of Sister Chromatid Cohesion (R-HSA-2500257) | 123 | 59 | 22 | 2.66 | 5.63E-09 | 7.89E-08 |
| Adaptive Immune System (R-HSA-1280218) | 826 | 231 | 149 | 1.55 | 6.73E-09 | 9.37E-08 |
| Hedgehog ligand biogenesis (R-HSA-5358346) | 64 | 40 | 12 | 3.47 | 6.96E-09 | 9.64E-08 |
| ER-Phagosome pathway (R-HSA-1236974) | 90 | 49 | 16 | 3.02 | 7.10E-09 | 9.72E-08 |
| Regulation of RUNX2 expression and activity (R-HSA-8939902) | 70 | 42 | 13 | 3.33 | 7.15E-09 | 9.75E-08 |
| Glycolysis (R-HSA-70171) | 67 | 41 | 12 | 3.4 | 7.09E-09 | 9.77E-08 |
| UCH proteinases (R-HSA-5689603) | 95 | 50 | 17 | 2.92 | 8.52E-09 | 1.16E-07 |
| Oxygen-dependent proline hydroxylation of Hypoxia-inducible Factor Alpha (R-HSA-1234176) | 65 | 40 | 12 | 3.42 | 9.59E-09 | 1.29E-07 |
| Transport of Mature mRNA derived from an Intron-Containing Transcript (R-HSA-159236) | 71 | 42 | 13 | 3.28 | 9.72E-09 | 1.30E-07 |
| Signalling by NOTCH4 (R-HSA-9013694) | 80 | 45 | 14 | 3.12 | 1.02E-08 | 1.37E-07 |
| MAPK6/MAPK4 signaling (R-HSA-5687128) | 88 | 48 | 16 | 3.03 | 1.03E-08 | 1.37E-07 |
| Mitotic Spindle Checkpoint (R-HSA-69618) | 109 | 54 | 20 | 2.75 | 1.05E-08 | 1.39E-07 |
| ABC transporter disorders (R-HSA-5619084) | 77 | 44 | 14 | 3.17 | 1.14E-08 | 1.49E-07 |
| Chromatin organization (R-HSA-4839726) | 240 | 91 | 43 | 2.1 | 1.20E-08 | 1.56E-07 |
| Chromatin modifying enzymes (R-HSA-3247509) | 240 | 91 | 43 | 2.1 | 1.20E-08 | 1.57E-07 |
| mRNA 3'-end processing (R-HSA-72187) | 57 | 37 | 10 | 3.6 | 1.22E-08 | 1.57E-07 |
| Antigen processing: Ubiquitination & Proteasome degradation (R-HSA-983168) | 308 | 108 | 55 | 1.95 | 1.23E-08 | 1.57E-07 |
| rRNA modification in the nucleus and cytosol (R-HSA-6790901) | 60 | 38 | 11 | 3.52 | 1.26E-08 | 1.62E-07 |
| Asymmetric localization of PCP proteins (R-HSA-4608870) | 63 | 39 | 11 | 3.44 | 1.30E-08 | 1.65E-07 |
| RNA Polymerase II Transcription Termination (R-HSA-73856) | 66 | 40 | 12 | 3.36 | 1.32E-08 | 1.66E-07 |
| Hedgehog 'off' state (R-HSA-5610787) | 112 | 55 | 20 | 2.73 | 1.34E-08 | 1.69E-07 |
| VEGFA-VEGFR2 Pathway (R-HSA-4420097) | 94 | 49 | 17 | 2.89 | 1.48E-08 | 1.85E-07 |
| Clathrin-mediated endocytosis (R-HSA-8856828) | 144 | 64 | 26 | 2.47 | 1.61E-08 | 2.01E-07 |
| tRNA processing in the nucleus (R-HSA-6784531) | 55 | 36 | 10 | 3.63 | 1.63E-08 | 2.02E-07 |
| Antiviral mechanism by IFN-stimulated genes (R-HSA-1169410) | 79 | 44 | 14 | 3.09 | 1.82E-08 | 2.24E-07 |
| Translocation of SLC2A4 (GLUT4) to the plasma membrane (R-HSA-1445148) | 72 | 42 | 13 | 3.24 | 1.91E-08 | 2.35E-07 |
| Downstream TCR signalling (R-HSA-202424) | 102 | 51 | 18 | 2.78 | 2.33E-08 | 2.85E-07 |
| ISG15 antiviral mechanism (R-HSA-1169408) | 71 | 41 | 13 | 3.21 | 3.24E-08 | 3.92E-07 |
| Transcription-Coupled Nucleotide Excision Repair (TC-NER) (R-HSA-6781827) | 78 | 43 | 14 | 3.06 | 3.23E-08 | 3.93E-07 |
| Regulation of RAS by GAPs (R-HSA-5658442) | 66 | 39 | 12 | 3.28 | 3.30E-08 | 3.98E-07 |
| TCR signalling (R-HSA-202403) | 124 | 57 | 22 | 2.55 | 3.42E-08 | 4.10E-07 |
| Signalling by VEGF (R-HSA-194138) | 104 | 51 | 19 | 2.72 | 3.45E-08 | 4.12E-07 |
| Deubiquitination (R-HSA-5688426) | 282 | 100 | 51 | 1.97 | 3.51E-08 | 4.17E-07 |
| Transcriptional regulation by RUNX2 (R-HSA-8878166) | 117 | 55 | 21 | 2.61 | 4.53E-08 | 5.35E-07 |
| Cellular response to hypoxia (R-HSA-1234174) | 74 | 41 | 13 | 3.08 | 6.20E-08 | 7.29E-07 |
| EML4 and NUDC in mitotic spindle formation (R-HSA-9648025) | 115 | 54 | 21 | 2.61 | 6.49E-08 | 7.59E-07 |
| Antigen processing-Cross presentation (R-HSA-1236975) | 106 | 51 | 19 | 2.67 | 7.05E-08 | 8.21E-07 |
| RNA Polymerase II Pre-transcription Events (R-HSA-674695) | 84 | 44 | 15 | 2.91 | 7.93E-08 | 9.16E-07 |
| Global Genome Nucleotide Excision Repair (GG-NER) (R-HSA-5696399) | 84 | 44 | 15 | 2.91 | 7.93E-08 | 9.20E-07 |
| Ub-specific processing proteases (R-HSA-5689880) | 204 | 78 | 37 | 2.12 | 9.99E-08 | 1.15E-06 |
| mRNA Splicing - Minor Pathway (R-HSA-72165) | 52 | 33 | 9 | 3.52 | 1.09E-07 | 1.24E-06 |
| MHC class II antigen presentation (R-HSA-2132295) | 126 | 56 | 23 | 2.47 | 1.21E-07 | 1.38E-06 |
| Organelle biogenesis and maintenance (R-HSA-1852241) | 293 | 100 | 53 | 1.89 | 1.28E-07 | 1.45E-06 |
| Amplification of signal from the kinetochores (R-HSA-141424) | 92 | 46 | 17 | 2.78 | 1.32E-07 | 1.49E-06 |
| Amplification of signal from unattached kinetochores via a MAD2 inhibitory signal (R-HSA-141444) | 92 | 46 | 17 | 2.78 | 1.32E-07 | 1.49E-06 |
| Metabolism of carbohydrates (R-HSA-71387) | 286 | 98 | 52 | 1.9 | 1.39E-07 | 1.56E-06 |
| ABC-family proteins mediated transport (R-HSA-382556) | 102 | 49 | 18 | 2.67 | 1.43E-07 | 1.59E-06 |
| Transcriptional regulation by RUNX3 (R-HSA-8878159) | 93 | 46 | 17 | 2.75 | 1.53E-07 | 1.70E-06 |
| Gap-filling DNA repair synthesis and ligation in TC-NER (R-HSA-6782210) | 64 | 37 | 12 | 3.21 | 1.64E-07 | 1.81E-06 |
| Beta-catenin independent WNT signalling (R-HSA-3858494) | 142 | 60 | 26 | 2.35 | 1.86E-07 | 2.04E-06 |
| Cytosolic tRNA aminoacylation (R-HSA-379716) | 24 | 22 | 4 | 5.09 | 1.93E-07 | 2.11E-06 |
| Golgi Associated Vesicle Biogenesis (R-HSA-432722) | 54 | 33 | 10 | 3.39 | 2.07E-07 | 2.25E-06 |
| RHOC GTPase cycle (R-HSA-9013106) | 73 | 39 | 13 | 2.97 | 2.51E-07 | 2.72E-06 |
| Metabolism of nucleotides (R-HSA-15869) | 92 | 45 | 17 | 2.72 | 2.55E-07 | 2.75E-06 |
| Immunoregulatory interactions between a Lymphoid and a non-Lymphoid cell (R-HSA-198933) | 197 | 8 | 35 | 0.23 | 2.57E-07 | 2.76E-06 |
| Nuclear Envelope (NE) Reassembly (R-HSA-2995410) | 70 | 38 | 13 | 3.01 | 2.70E-07 | 2.89E-06 |
| Signalling by Hedgehog (R-HSA-5358351) | 148 | 61 | 27 | 2.29 | 3.06E-07 | 3.26E-06 |
| Assembly of the pre-replicative complex (R-HSA-68867) | 99 | 47 | 18 | 2.64 | 3.25E-07 | 3.45E-06 |
| Metabolism of non-coding RNA (R-HSA-194441) | 50 | 31 | 9 | 3.44 | 3.80E-07 | 4.00E-06 |
| Signalling by ALK in cancer (R-HSA-9700206) | 53 | 32 | 10 | 3.35 | 3.84E-07 | 4.01E-06 |
| snRNP Assembly (R-HSA-191859) | 50 | 31 | 9 | 3.44 | 3.80E-07 | 4.01E-06 |
| Signalling by ALK fusions and activated point mutants (R-HSA-9725370) | 53 | 32 | 10 | 3.35 | 3.84E-07 | 4.03E-06 |
| Signalling by WNT (R-HSA-195721) | 297 | 99 | 54 | 1.85 | 4.43E-07 | 4.61E-06 |
| Signalling by Interleukins (R-HSA-449147) | 448 | 135 | 81 | 1.67 | 4.55E-07 | 4.71E-06 |
| Regulation of TP53 Activity (R-HSA-5633007) | 159 | 63 | 29 | 2.2 | 4.85E-07 | 4.99E-06 |
| MAPK family signalling cascades (R-HSA-5683057) | 308 | 101 | 55 | 1.82 | 4.98E-07 | 5.11E-06 |
| TNFR2 non-canonical NF-kB pathway (R-HSA-5668541) | 98 | 46 | 18 | 2.61 | 5.21E-07 | 5.31E-06 |
| RHOA GTPase cycle (R-HSA-8980692) | 148 | 60 | 27 | 2.25 | 5.21E-07 | 5.33E-06 |
| Transcriptional regulation by RUNX1 (R-HSA-8878171) | 204 | 75 | 37 | 2.04 | 5.57E-07 | 5.64E-06 |
| Hedgehog 'on' state (R-HSA-5632684) | 84 | 42 | 15 | 2.78 | 5.67E-07 | 5.73E-06 |
| Cytosolic sensors of pathogen-associated DNA (R-HSA-1834949) | 63 | 35 | 11 | 3.08 | 5.80E-07 | 5.83E-06 |
| Potential therapeutics for SARS (R-HSA-9679191) | 85 | 42 | 15 | 2.74 | 6.28E-07 | 6.29E-06 |
| Neddylation (R-HSA-8951664) | 233 | 82 | 42 | 1.95 | 7.58E-07 | 7.56E-06 |
| RNA Polymerase II Transcription Elongation (R-HSA-75955) | 61 | 34 | 11 | 3.09 | 8.18E-07 | 8.09E-06 |
| Formation of RNA Pol II elongation complex (R-HSA-112382) | 61 | 34 | 11 | 3.09 | 8.18E-07 | 8.12E-06 |
| HSP90 chaperone cycle for steroid hormone receptors (SHR) in the presence of ligand (R-HSA-3371497) | 57 | 33 | 10 | 3.21 | 8.44E-07 | 8.32E-06 |
| The citric acid (TCA) cycle and respiratory electron transport (R-HSA-1428517) | 176 | 67 | 32 | 2.11 | 8.91E-07 | 8.74E-06 |
| HCMV Infection (R-HSA-9609646) | 125 | 53 | 23 | 2.35 | 9.02E-07 | 8.82E-06 |
| Dual incision in TC-NER (R-HSA-6782135) | 65 | 35 | 12 | 2.99 | 9.19E-07 | 8.95E-06 |
| Autophagy (R-HSA-9612973) | 150 | 60 | 27 | 2.22 | 9.39E-07 | 9.11E-06 |
| RAF/MAP kinase cascade (R-HSA-5673001) | 264 | 89 | 48 | 1.87 | 1.01E-06 | 9.73E-06 |
| TCF dependent signalling in response to WNT (R-HSA-201681) | 201 | 73 | 36 | 2.02 | 1.09E-06 | 1.05E-05 |
| Transport of Mature mRNA Derived from an Intronless Transcript(R-HSA-159231) | 39 | 26 | 7 | 3.7 | 1.21E-06 | 1.16E-05 |
| MAPK1/MAPK3 signaling (R-HSA-5684996) | 269 | 90 | 48 | 1.86 | 1.23E-06 | 1.17E-05 |
| Apoptotic execution phase (R-HSA-75153) | 51 | 30 | 9 | 3.27 | 1.31E-06 | 1.25E-05 |
| Potassium Channels (R-HSA-1296071) | 103 | 1 | 19 | 0.05 | 1.45E-06 | 1.37E-05 |
| Interactions of Rev with host cellular proteins (R-HSA-177243) | 34 | 24 | 6 | 3.92 | 1.50E-06 | 1.41E-05 |
| Transcription of the HIV genome (R-HSA-167172) | 73 | 37 | 13 | 2.81 | 1.53E-06 | 1.44E-05 |
| Transport of Mature mRNAs Derived from Intronless Transcripts (R-HSA-159234) | 40 | 26 | 7 | 3.61 | 1.70E-06 | 1.60E-05 |
| SUMOylation of DNA replication proteins (R-HSA-4615885) | 43 | 27 | 8 | 3.49 | 1.76E-06 | 1.65E-05 |
| Disorders of transmembrane transporters (R-HSA-5619115) | 173 | 65 | 31 | 2.09 | 1.77E-06 | 1.65E-05 |
| Retrograde transport at the Trans-Golgi-Network (R-HSA-6811440) | 49 | 29 | 9 | 3.29 | 1.79E-06 | 1.66E-05 |
| Epigenetic regulation of gene expression (R-HSA-212165) | 115 | 49 | 21 | 2.37 | 1.84E-06 | 1.69E-05 |
| RHOB GTPase cycle (R-HSA-9013026) | 70 | 36 | 13 | 2.85 | 1.83E-06 | 1.69E-05 |
| AURKA Activation by TPX2 (R-HSA-8854518) | 71 | 36 | 13 | 2.81 | 2.16E-06 | 1.97E-05 |
| RHOBTB GTPase Cycle (R-HSA-9706574) | 35 | 24 | 6 | 3.81 | 2.16E-06 | 1.98E-05 |
| COPI-dependent Golgi-to-ER retrograde traffic (R-HSA-6811434) | 101 | 45 | 18 | 2.47 | 2.24E-06 | 2.04E-05 |
| Regulation of PLK1 Activity at G2/M Transition (R-HSA-2565942) | 85 | 40 | 15 | 2.61 | 2.33E-06 | 2.11E-05 |
| Viral Messenger RNA Synthesis (R-HSA-168325) | 41 | 26 | 7 | 3.52 | 2.38E-06 | 2.15E-05 |
| Macroautophagy (R-HSA-1632852) | 135 | 54 | 24 | 2.22 | 3.31E-06 | 2.97E-05 |
| Formation of TC-NER Pre-Incision Complex (R-HSA-6781823) | 53 | 30 | 10 | 3.14 | 3.42E-06 | 3.05E-05 |
| SUMOylation of chromatin organization proteins (R-HSA-4551638) | 53 | 30 | 10 | 3.14 | 3.42E-06 | 3.06E-05 |
| Recruitment of mitotic centrosome proteins and complexes (R-HSA-380270) | 80 | 38 | 14 | 2.64 | 3.86E-06 | 3.43E-05 |
| Centrosome maturation (R-HSA-380287) | 80 | 38 | 14 | 2.64 | 3.86E-06 | 3.44E-05 |
| Creation of C4 and C2 activators (R-HSA-166786) | 77 | 0 | 14 | < 0.01 | 4.15E-06 | 3.66E-05 |
| SUMOylation of RNA binding proteins (R-HSA-4570464) | 43 | 26 | 8 | 3.36 | 4.53E-06 | 3.99E-05 |
| Toll Like Receptor 3 (TLR3) Cascade (R-HSA-168164) | 92 | 41 | 17 | 2.47 | 5.12E-06 | 4.49E-05 |
| Rev-mediated nuclear export of HIV RNA (R-HSA-165054) | 32 | 22 | 6 | 3.82 | 5.51E-06 | 4.82E-05 |
| SLC-mediated transmembrane transport (R-HSA-425407) | 247 | 16 | 45 | 0.36 | 5.96E-06 | 5.20E-05 |
| NS1 Mediated Effects on Host Pathways (R-HSA-168276) | 38 | 24 | 7 | 3.51 | 6.06E-06 | 5.27E-05 |
| MyD88-independent TLR4 cascade (R-HSA-166166) | 96 | 42 | 17 | 2.43 | 6.96E-06 | 6.01E-05 |
| TRIF(TICAM1)-mediated TLR4 signalling (R-HSA-937061) | 96 | 42 | 17 | 2.43 | 6.96E-06 | 6.03E-05 |
| Rab regulation of trafficking (R-HSA-9007101) | 121 | 49 | 22 | 2.25 | 7.87E-06 | 6.70E-05 |
| Protein folding (R-HSA-391251) | 97 | 42 | 17 | 2.4 | 7.80E-06 | 6.71E-05 |
| Formation of the HIV-1 Early Elongation Complex (R-HSA-167158) | 33 | 22 | 6 | 3.7 | 7.86E-06 | 6.71E-05 |
| Formation of the Early Elongation Complex (R-HSA-113418) | 33 | 22 | 6 | 3.7 | 7.86E-06 | 6.74E-05 |
| Pyruvate metabolism and Citric Acid (TCA) cycle (R-HSA-71406) | 54 | 29 | 10 | 2.98 | 8.56E-06 | 7.24E-05 |
| RHOJ GTPase cycle (R-HSA-9013409) | 54 | 29 | 10 | 2.98 | 8.56E-06 | 7.26E-05 |
| RUNX1 regulates transcription of genes involved in differentiation of HSCs (R-HSA-8939236) | 98 | 42 | 18 | 2.38 | 8.90E-06 | 7.49E-05 |
| Signalling by GPCR (R-HSA-372790) | 697 | 76 | 126 | 0.61 | 9.12E-06 | 7.66E-05 |
| Recruitment of NuMA to mitotic centrosomes (R-HSA-380320) | 94 | 41 | 17 | 2.42 | 9.81E-06 | 8.21E-05 |
| Interferon Signalling (R-HSA-913531) | 197 | 68 | 35 | 1.92 | 9.86E-06 | 8.22E-05 |
| Nuclear import of Rev protein (R-HSA-180746) | 31 | 21 | 6 | 3.76 | 1.05E-05 | 8.71E-05 |
| Loss of Nlp from mitotic centrosomes (R-HSA-380259) | 68 | 33 | 12 | 2.69 | 1.05E-05 | 8.72E-05 |
| Loss of proteins required for interphase microtubule organization from the centrosome (R-HSA-380284) | 68 | 33 | 12 | 2.69 | 1.05E-05 | 8.75E-05 |
| Interactions of Vpr with host cellular proteins (R-HSA-176033) | 34 | 22 | 6 | 3.59 | 1.11E-05 | 9.13E-05 |
| Gene and protein expression by JAK-STAT signalling after Interleukin-12 stimulation (R-HSA-8950505) | 37 | 23 | 7 | 3.45 | 1.14E-05 | 9.35E-05 |
| Fc epsilon receptor (FCERI) signalling (R-HSA-2454202) | 187 | 65 | 34 | 1.93 | 1.32E-05 | 1.08E-04 |
| SUMOylation of DNA damage response and repair proteins (R-HSA-3108214) | 71 | 34 | 13 | 2.66 | 1.41E-05 | 1.15E-04 |
| Classical antibody-mediated complement activation (R-HSA-173623) | 70 | 0 | 13 | < 0.01 | 1.52E-05 | 1.24E-04 |
| RHOF GTPase cycle (R-HSA-9035034) | 41 | 24 | 7 | 3.25 | 1.56E-05 | 1.26E-04 |
| Initial triggering of complement (R-HSA-166663) | 85 | 1 | 15 | 0.07 | 1.60E-05 | 1.29E-04 |
| Signalling by NTRKs (R-HSA-166520) | 131 | 50 | 24 | 2.12 | 1.69E-05 | 1.36E-04 |
| HSF1-dependent transactivation (R-HSA-3371571) | 24 | 18 | 4 | 4.16 | 1.69E-05 | 1.36E-04 |
| Signalling by NTRK1 (TRKA) (R-HSA-187037) | 112 | 45 | 20 | 2.23 | 1.71E-05 | 1.36E-04 |
| L1CAM interactions (R-HSA-373760) | 119 | 47 | 21 | 2.19 | 1.75E-05 | 1.39E-04 |
| RNA Polymerase I Transcription Initiation (R-HSA-73762) | 46 | 26 | 8 | 3.14 | 1.76E-05 | 1.40E-04 |
| RHOQ GTPase cycle (R-HSA-9013406) | 59 | 30 | 11 | 2.82 | 1.85E-05 | 1.47E-04 |
| G alpha (s) signalling events (R-HSA-418555) | 145 | 6 | 26 | 0.23 | 1.88E-05 | 1.49E-04 |
| Metabolism of water-soluble vitamins and cofactors (R-HSA-196849) | 120 | 47 | 22 | 2.17 | 1.91E-05 | 1.50E-04 |
| Export of Viral Ribonucleoproteins from Nucleus (R-HSA-168274) | 30 | 20 | 5 | 3.7 | 2.02E-05 | 1.58E-04 |
| Extension of Telomeres (R-HSA-180786) | 51 | 27 | 9 | 2.94 | 2.10E-05 | 1.63E-04 |
| RHOD GTPase cycle (R-HSA-9013405) | 51 | 27 | 9 | 2.94 | 2.10E-05 | 1.64E-04 |
| RAF activation (R-HSA-5673000) | 33 | 21 | 6 | 3.53 | 2.10E-05 | 1.64E-04 |
| RHOU GTPase cycle (R-HSA-9013420) | 39 | 23 | 7 | 3.27 | 2.13E-05 | 1.65E-04 |
| Complement cascade (R-HSA-166658) | 120 | 4 | 22 | 0.19 | 2.53E-05 | 1.95E-04 |
| RHOBTB2 GTPase cycle (R-HSA-9013418) | 23 | 17 | 4 | 4.1 | 3.30E-05 | 2.54E-04 |
| RHOG GTPase cycle (R-HSA-9013408) | 73 | 33 | 13 | 2.51 | 3.39E-05 | 2.60E-04 |
| HIV Transcription Elongation (R-HSA-167169) | 46 | 25 | 8 | 3.02 | 3.46E-05 | 2.62E-04 |
| Formation of HIV-1 elongation complex containing HIV-1 Tat (R-HSA-167200) | 46 | 25 | 8 | 3.02 | 3.46E-05 | 2.63E-04 |
| Cilium Assembly (R-HSA-5617833) | 201 | 67 | 36 | 1.85 | 3.48E-05 | 2.63E-04 |
| Tat-mediated elongation of the HIV-1 transcript (R-HSA-167246) | 46 | 25 | 8 | 3.02 | 3.46E-05 | 2.64E-04 |
| Interleukin-12 signalling (R-HSA-9020591) | 46 | 25 | 8 | 3.02 | 3.46E-05 | 2.64E-04 |
| Toll Like Receptor 10 (TLR10) Cascade (R-HSA-168142) | 84 | 36 | 15 | 2.38 | 3.62E-05 | 2.71E-04 |
| Toll Like Receptor 5 (TLR5) Cascade (R-HSA-168176) | 84 | 36 | 15 | 2.38 | 3.62E-05 | 2.72E-04 |
| MyD88 cascade initiated on plasma membrane (R-HSA-975871) | 84 | 36 | 15 | 2.38 | 3.62E-05 | 2.73E-04 |
| EPH-Ephrin signalling (R-HSA-2682334) | 91 | 38 | 16 | 2.32 | 3.78E-05 | 2.82E-04 |
| Olfactory Signalling Pathway (R-HSA-381753) | 66 | 0 | 12 | < 0.01 | 3.86E-05 | 2.84E-04 |
| Peptide ligand-binding receptors (R-HSA-375276) | 196 | 12 | 35 | 0.34 | 3.85E-05 | 2.84E-04 |
| Transport of Ribonucleoproteins into the Host Nucleus (R-HSA-168271) | 29 | 19 | 5 | 3.64 | 3.84E-05 | 2.84E-04 |
| NEP/NS2 Interacts with the Cellular Export Machinery (R-HSA-168333) | 29 | 19 | 5 | 3.64 | 3.84E-05 | 2.85E-04 |
| mRNA Capping (R-HSA-72086) | 29 | 19 | 5 | 3.64 | 3.84E-05 | 2.86E-04 |
| SUMOylation of SUMOylation proteins (R-HSA-4085377) | 32 | 20 | 6 | 3.47 | 3.96E-05 | 2.89E-04 |
| DNA strand elongation (R-HSA-69190) | 32 | 20 | 6 | 3.47 | 3.96E-05 | 2.89E-04 |
| Transport of the SLBP independent Mature mRNA (R-HSA-159227) | 32 | 20 | 6 | 3.47 | 3.96E-05 | 2.90E-04 |
| Protein ubiquitination (R-HSA-8852135) | 74 | 33 | 13 | 2.48 | 4.02E-05 | 2.92E-04 |
| RNA Polymerase II Transcription Initiation And Promoter Clearance (R-HSA-76042) | 47 | 25 | 8 | 2.95 | 4.18E-05 | 2.98E-04 |
| HIV Transcription Initiation (R-HSA-167161) | 47 | 25 | 8 | 2.95 | 4.18E-05 | 2.98E-04 |
| RNA Polymerase II HIV Promoter Escape (R-HSA-167162) | 47 | 25 | 8 | 2.95 | 4.18E-05 | 2.99E-04 |
| Post-translational modification: synthesis of GPI-anchored proteins (R-HSA-163125) | 93 | 2 | 17 | 0.12 | 4.13E-05 | 3.00E-04 |
| RNA Polymerase II Promoter Escape (R-HSA-73776) | 47 | 25 | 8 | 2.95 | 4.18E-05 | 3.00E-04 |
| RNA Polymerase II Transcription Pre-Initiation And Promoter Opening (R-HSA-73779) | 47 | 25 | 8 | 2.95 | 4.18E-05 | 3.01E-04 |
| RNA Polymerase II Transcription Initiation (R-HSA-75953) | 47 | 25 | 8 | 2.95 | 4.18E-05 | 3.02E-04 |
| Formation of Incision Complex in GG-NER (R-HSA-5696395) | 43 | 24 | 8 | 3.1 | 4.29E-05 | 3.05E-04 |
| Interleukin-12 family signalling (R-HSA-447115) | 56 | 28 | 10 | 2.78 | 4.34E-05 | 3.06E-04 |
| RHO GTPases activate PAKs (R-HSA-5627123) | 21 | 16 | 4 | 4.23 | 4.34E-05 | 3.06E-04 |
| Branched-chain amino acid catabolism (R-HSA-70895) | 21 | 16 | 4 | 4.23 | 4.34E-05 | 3.07E-04 |
| SARS-CoV-2 Infection (R-HSA-9694516) | 71 | 32 | 13 | 2.5 | 4.74E-05 | 3.33E-04 |
| Postmitotic nuclear pore complex (NPC) reformation (R-HSA-9615933) | 24 | 17 | 4 | 3.93 | 4.84E-05 | 3.39E-04 |
| Signalling by NOTCH (R-HSA-157118) | 201 | 66 | 36 | 1.82 | 5.04E-05 | 3.52E-04 |
| Formation of HIV elongation complex in the absence of HIV Tat (R-HSA-167152) | 48 | 25 | 9 | 2.89 | 5.14E-05 | 3.58E-04 |
| RNA Pol II CTD phosphorylation and interaction with CE during HIV infection (R-HSA-167160) | 27 | 18 | 5 | 3.7 | 5.19E-05 | 3.59E-04 |
| RNA Pol II CTD phosphorylation and interaction with CE (R-HSA-77075) | 27 | 18 | 5 | 3.7 | 5.19E-05 | 3.60E-04 |
| Gluconeogenesis (R-HSA-70263) | 33 | 20 | 6 | 3.36 | 5.45E-05 | 3.75E-04 |
| Transport of the SLBP Dependant Mature mRNA (R-HSA-159230) | 33 | 20 | 6 | 3.36 | 5.45E-05 | 3.77E-04 |
| RHO GTPases Activate ROCKs (R-HSA-5627117) | 19 | 15 | 3 | 4.38 | 5.65E-05 | 3.88E-04 |
| Nucleotide biosynthesis (R-HSA-8956320) | 14 | 13 | 3 | 5.15 | 5.78E-05 | 3.96E-04 |
| Antimicrobial peptides (R-HSA-6803157) | 91 | 2 | 16 | 0.12 | 6.26E-05 | 4.27E-04 |
| MTOR signalling (R-HSA-165159) | 41 | 23 | 7 | 3.11 | 6.30E-05 | 4.29E-04 |
| Response to elevated platelet cytosolic Ca2+ (R-HSA-76005) | 132 | 48 | 24 | 2.02 | 6.71E-05 | 4.56E-04 |
| Deadenylation of mRNA (R-HSA-429947) | 25 | 17 | 5 | 3.77 | 6.98E-05 | 4.73E-04 |
| Prefoldin mediated transfer of substrate to CCT/TriC (R-HSA-389957) | 28 | 18 | 5 | 3.57 | 7.31E-05 | 4.93E-04 |
| Chromosome Maintenance (R-HSA-73886) | 120 | 45 | 22 | 2.08 | 7.30E-05 | 4.93E-04 |
| Vpr-mediated nuclear import of PICs (R-HSA-180910) | 31 | 19 | 6 | 3.4 | 7.45E-05 | 5.01E-04 |
| Toll Like Receptor 9 (TLR9) Cascade (R-HSA-168138) | 94 | 38 | 17 | 2.24 | 7.64E-05 | 5.12E-04 |
| HCMV Early Events (R-HSA-9609690) | 101 | 40 | 18 | 2.2 | 8.00E-05 | 5.35E-04 |
| Signalling by the B Cell Receptor (BCR) (R-HSA-983705) | 169 | 57 | 30 | 1.87 | 8.48E-05 | 5.65E-04 |
| mRNA decay by 5' to 3' exoribonuclease (R-HSA-430039) | 15 | 13 | 3 | 4.81 | 9.28E-05 | 6.17E-04 |
| Abortive elongation of HIV-1 transcript in the absence of Tat (R-HSA-167242) | 23 | 16 | 4 | 3.86 | 9.37E-05 | 6.20E-04 |
| RHOBTB1 GTPase cycle (R-HSA-9013422) | 23 | 16 | 4 | 3.86 | 9.37E-05 | 6.21E-04 |
| FGFR2 alternative splicing (R-HSA-6803529) | 26 | 17 | 5 | 3.63 | 9.91E-05 | 6.54E-04 |
| RHO GTPases Activate WASPs and WAVEs (R-HSA-5663213) | 35 | 20 | 6 | 3.17 | 1.00E-04 | 6.55E-04 |
| Lysosome Vesicle Biogenesis (R-HSA-432720) | 35 | 20 | 6 | 3.17 | 1.00E-04 | 6.56E-04 |
| Generic Transcription Pathway (R-HSA-212436) | 1197 | 279 | 216 | 1.29 | 1.01E-04 | 6.57E-04 |
| SUMOylation of ubiquitinylation proteins (R-HSA-3232142) | 35 | 20 | 6 | 3.17 | 1.00E-04 | 6.58E-04 |
| TBC/RABGAPs (R-HSA-8854214) | 44 | 23 | 8 | 2.9 | 1.02E-04 | 6.66E-04 |
| MAP kinase activation (R-HSA-450294) | 63 | 29 | 11 | 2.56 | 1.04E-04 | 6.74E-04 |
| RNA polymerase II transcribes snRNA genes (R-HSA-6807505) | 74 | 32 | 13 | 2.4 | 1.06E-04 | 6.83E-04 |
| Anti-inflammatory response favouring Leishmania parasite infection (R-HSA-9662851) | 229 | 17 | 41 | 0.41 | 1.10E-04 | 7.10E-04 |
| Leishmania parasite growth and survival (R-HSA-9664433) | 229 | 17 | 41 | 0.41 | 1.10E-04 | 7.12E-04 |
| Regulation of Complement cascade (R-HSA-977606) | 110 | 4 | 20 | 0.2 | 1.13E-04 | 7.24E-04 |
| Metabolism of vitamins and cofactors (R-HSA-196854) | 186 | 61 | 34 | 1.82 | 1.14E-04 | 7.30E-04 |
| Mitochondrial tRNA aminoacylation (R-HSA-379726) | 21 | 15 | 4 | 3.97 | 1.25E-04 | 8.01E-04 |
| CDC42 GTPase cycle (R-HSA-9013148) | 154 | 53 | 28 | 1.91 | 1.30E-04 | 8.28E-04 |
| TP53 Regulates Transcription of DNA Repair Genes (R-HSA-6796648) | 65 | 29 | 12 | 2.48 | 1.31E-04 | 8.32E-04 |
| Platelet activation, signalling and aggregation (R-HSA-76002) | 260 | 78 | 47 | 1.67 | 1.31E-04 | 8.32E-04 |
| Chemokine receptors bind chemokines (R-HSA-380108) | 57 | 0 | 10 | < 0.01 | 1.33E-04 | 8.39E-04 |
| FCGR activation (R-HSA-2029481) | 74 | 1 | 13 | 0.08 | 1.34E-04 | 8.44E-04 |
| Nuclear Pore Complex (NPC) Disassembly (R-HSA-3301854) | 33 | 19 | 6 | 3.2 | 1.38E-04 | 8.67E-04 |
| VEGFR2 mediated vascular permeability (R-HSA-5218920) | 27 | 17 | 5 | 3.5 | 1.39E-04 | 8.69E-04 |
| TRAF6 mediated induction of NFkB and MAP kinases upon TLR7/8 or 9 activation (R-HSA-975138) | 89 | 36 | 16 | 2.25 | 1.38E-04 | 8.69E-04 |
| Synthesis of active ubiquitin: roles of E1 and E2 enzymes (R-HSA-8866652) | 30 | 18 | 5 | 3.33 | 1.40E-04 | 8.73E-04 |
| TP53 Regulates Metabolic Genes (R-HSA-5628897) | 83 | 34 | 15 | 2.27 | 1.41E-04 | 8.79E-04 |
| MyD88 dependent cascade initiated on endosome (R-HSA-975155) | 90 | 36 | 16 | 2.22 | 1.48E-04 | 9.19E-04 |
| Toll Like Receptor 7/8 (TLR7/8) Cascade (R-HSA-168181) | 91 | 36 | 16 | 2.2 | 1.61E-04 | 9.93E-04 |
| Chaperonin-mediated protein folding (R-HSA-390466) | 91 | 36 | 16 | 2.2 | 1.61E-04 | 9.96E-04 |
| Unfolded Protein Response (UPR) (R-HSA-381119) | 91 | 36 | 16 | 2.2 | 1.61E-04 | 9.98E-04 |

# Supplementary Table 5

| **Breast cancer cells** | | | | | | | | |
| --- | --- | --- | --- | --- | --- | --- | --- | --- |
| **Gene symbol** | **Sample 1** | **Sample 2** | **Sample 3** | **Sample 4** | **Sample 5** | **Sample 6** | **Sample 7** | **Sample 8** |
| AHNAK2 | 11,31 | 11,25 | 10,00 | 9,82 | 11,25 | 11,01 | 10,02 | 9,91 |
| AKT1 | 8,28 | 8,36 | 8,20 | 9,12 | 8,59 | 8,38 | 8,98 | 9,26 |
| CASP8 | 7,98 | 8,17 | 9,02 | 8,48 | 7,24 | 7,34 | 9,04 | 8,29 |
| CDH1 | 9,59 | 9,34 | 9,57 | 9,36 | 10,54 | 10,51 | 9,95 | 10,00 |
| CDH2 | 3,51 | 4,11 | 3,56 | 3,96 | 3,98 | 3,72 | 5,37 | 4,16 |
| DPYSL2 | 11,21 | 11,13 | 11,25 | 11,28 | 11,88 | 11,82 | 13,26 | 12,99 |
| DPYSL5 | 9,19 | 9,14 | 8,64 | 8,24 | 9,40 | 10,96 | 8,76 | 8,66 |
| GIT1 | 7,94 | 7,80 | 8,04 | 7,69 | 7,57 | 7,71 | 7,79 | 7,54 |
| GRB2 | 11,25 | 11,26 | 10,50 | 11,76 | 10,72 | 10,79 | 10,07 | 10,28 |
| MAP2K4 | 8,55 | 8,66 | 9,00 | 8,76 | 7,68 | 8,17 | 8,64 | 8,53 |
| MUC1 | 9,39 | 9,49 | 9,25 | 9,15 | 10,31 | 10,71 | 9,49 | 10,07 |
| MUC5B | 5,37 | 5,58 | 6,97 | 6,70 | 6,42 | 5,82 | 6,24 | 6,64 |
| PTPN11 | 11,63 | 11,58 | 11,82 | 11,60 | 10,98 | 11,42 | 11,47 | 11,43 |
| PXN | 7,85 | 7,79 | 7,74 | 8,20 | 8,68 | 8,48 | 7,76 | 7,93 |
| RAC1 | 10,41 | 10,29 | 12,02 | 11,46 | 9,58 | 9,72 | 10,98 | 10,09 |
| RTN4 | 8,94 | 8,81 | 8,35 | 8,33 | 9,08 | 9,35 | 8,60 | 8,96 |
| SHANK2 | 7,26 | 7,33 | 7,11 | 7,98 | 8,06 | 8,23 | 7,77 | 8,67 |
| TGFBR2 | 5,45 | 5,20 | 5,68 | 5,35 | 6,74 | 6,77 | 5,51 | 6,19 |
|  |  |  |  |  |  |  |  |  |
| **Breast cancer cells (NPC)** | | | | | | | | |
| **Gene symbol** | **Sample 1** | **Sample 2** | **Sample 3** | **Sample 4** | **Sample 5** | **Sample 6** | **Sample 7** | **Sample 8** |
| AHNAK2 | 10,94 | 10,99 | 10,67 | 10,58 | 12,21 | 12,17 | 12,02 | 10,26 |
| AKT1 | 8,20 | 8,10 | 8,30 | 8,17 | 8,74 | 8,76 | 7,85 | 8,03 |
| CASP8 | 8,22 | 8,42 | 8,24 | 8,34 | 8,79 | 8,79 | 7,63 | 7,87 |
| CDH1 | 7,32 | 7,21 | 7,88 | 7,54 | 7,41 | 7,42 | 8,59 | 7,71 |
| CDH2 | 3,32 | 3,03 | 6,38 | 6,55 | 3,59 | 3,54 | 6,27 | 5,54 |
| DPYSL2 | 11,47 | 11,40 | 11,41 | 11,36 | 12,91 | 12,96 | 13,41 | 13,79 |
| DPYSL5 | 10,82 | 10,62 | 10,15 | 10,81 | 8,94 | 8,90 | 11,52 | 8,51 |
| GIT1 | 7,07 | 6,65 | 7,99 | 7,38 | 7,78 | 7,70 | 6,71 | 6,88 |
| GRB2 | 9,81 | 9,97 | 9,82 | 10,82 | 10,35 | 10,36 | 9,68 | 9,80 |
| MAP2K4 | 8,70 | 8,71 | 8,63 | 8,85 | 8,92 | 8,86 | 8,07 | 8,26 |
| MUC1 | 9,23 | 9,44 | 9,43 | 9,31 | 11,01 | 11,00 | 11,78 | 8,90 |
| MUC5B | 5,94 | 5,78 | 5,78 | 5,86 | 8,38 | 8,33 | 6,97 | 6,04 |
| PTPN11 | 11,50 | 11,48 | 11,13 | 10,96 | 11,74 | 11,78 | 10,78 | 11,16 |
| PXN | 8,80 | 8,57 | 8,22 | 8,03 | 8,56 | 8,68 | 8,35 | 8,17 |
| RAC1 | 10,93 | 11,03 | 10,56 | 10,68 | 11,10 | 10,94 | 9,39 | 9,97 |
| RTN4 | 8,94 | 9,21 | 9,41 | 8,70 | 10,04 | 9,99 | 9,80 | 9,61 |
| SHANK2 | 6,68 | 6,71 | 7,01 | 7,10 | 6,31 | 6,28 | 6,53 | 6,14 |
| TGFBR2 | 5,57 | 5,36 | 5,43 | 5,54 | 7,21 | 7,26 | 6,35 | 5,38 |

# Supplementary Table 6

|  | | | **MCF7** | | | **MCF7(NPC)** | | |
| --- | --- | --- | --- | --- | --- | --- | --- | --- |
| **Gene symbol** | **Sample 1** | **Sample 2** | | **Sample 3** | **Sample 1** | | **Sample 2** | **Sample 3** |
| ABI1 | 6,4 | 6,3 | | 6,5 | 6,1 | | 6,0 | 5,9 |
| AHNAK | 13,5 | 13,6 | | 13,6 | 13,0 | | 13,0 | 13,0 |
| AHNAK2 | 7,9 | 7,7 | | 7,9 | 8,2 | | 8,0 | 8,2 |
| AKT1 | 8,5 | 8,7 | | 8,5 | 8,8 | | 8,8 | 9,0 |
| ARHGAP35 | 7,9 | 7,7 | | 7,6 | 7,8 | | 7,8 | 7,7 |
| BRAF | 2,8 | 3,3 | | 3,5 | 3,6 | | 3,4 | 3,7 |
| BRCA1 | 2,9 | 2,7 | | 2,9 | 2,5 | | 2,8 | 2,6 |
| BRCA2 | 3,5 | 3,1 | | 3,5 | 3,4 | | 3,5 | 2,9 |
| CALD1 | 6,9 | 6,8 | | 6,7 | 6,9 | | 7,1 | 7,0 |
| CASP8 | 7,5 | 7,5 | | 7,4 | 7,3 | | 7,3 | 7,3 |
| CDC42 | 4,6 | 4,0 | | 5,0 | 3,4 | | 4,3 | 3,9 |
| CDH1 | 7,2 | 7,2 | | 7,1 | 7,4 | | 7,2 | 7,3 |
| CDH11 | 4,9 | 4,7 | | 5,4 | 5,1 | | 5,0 | 5,2 |
| CLPP | 6,8 | 6,8 | | 6,8 | 7,3 | | 7,2 | 7,1 |
| CTNNA2 | 8,7 | 8,7 | | 8,4 | 8,3 | | 8,4 | 8,4 |
| CTNNB1 | 7,3 | 7,6 | | 7,3 | 6,9 | | 6,9 | 7,1 |
| EGFR | 6,0 | 5,8 | | 5,6 | 5,4 | | 5,7 | 5,8 |
| ESR1 | 3,6 | 3,8 | | 3,7 | 5,4 | | 5,3 | 5,4 |
| FASN | 11,5 | 11,5 | | 11,4 | 12,0 | | 12,0 | 12,1 |
| FGFR1 | 2,8 | 2,7 | | 3,4 | 3,4 | | 3,1 | 3,2 |
| FGFR1OP | 4,3 | 4,8 | | 4,8 | 4,4 | | 4,7 | 4,5 |
| FGFR1OP2 | 4,9 | 4,8 | | 4,8 | 4,9 | | 4,9 | 4,6 |
| KRAS | 5,3 | 5,1 | | 5,3 | 5,0 | | 4,9 | 5,0 |
| MAP2K4 | 6,7 | 6,6 | | 6,6 | 6,0 | | 6,3 | 6,4 |
| MAPT | 7,5 | 7,6 | | 7,7 | 7,1 | | 7,0 | 6,9 |
| MKI67 | 9,7 | 9,4 | | 9,5 | 8,9 | | 8,8 | 8,8 |
| MTOR | 7,6 | 7,5 | | 7,5 | 7,2 | | 7,3 | 7,3 |
| MUC1 | 6,2 | 6,1 | | 6,2 | 7,2 | | 7,2 | 7,3 |
| MUC5B | 6,4 | 6,4 | | 6,4 | 6,5 | | 6,5 | 6,5 |
| NRAS | 7,1 | 6,8 | | 7,0 | 7,0 | | 7,1 | 6,9 |
| NUP93 | 8,4 | 8,4 | | 8,3 | 8,6 | | 8,6 | 8,7 |
| PAK4 | 7,8 | 7,9 | | 8,0 | 7,8 | | 7,9 | 7,8 |
| PKP2 | 6,5 | 6,4 | | 6,4 | 6,4 | | 6,2 | 6,3 |
| PTEN | 6,6 | 6,6 | | 6,5 | 6,3 | | 6,3 | 6,2 |
| PTPN11 | 8,1 | 8,1 | | 8,1 | 8,0 | | 8,0 | 7,9 |
| PXN | 7,0 | 7,2 | | 7,0 | 6,6 | | 6,7 | 6,3 |
| RAC1 | 6,3 | 6,4 | | 6,4 | 5,3 | | 5,3 | 5,4 |
| RB1 | 7,9 | 7,8 | | 8,0 | 8,0 | | 7,9 | 7,9 |
| RHOA | 3,6 | 3,8 | | 4,1 | 4,4 | | 3,8 | 3,4 |
| RIPK2 | 4,0 | 4,2 | | 4,2 | 4,5 | | 3,9 | 4,1 |
| ROBO1 | 2,8 | 3,1 | | 3,0 | 3,9 | | 3,5 | 2,9 |
| ROCK1 | 7,5 | 7,4 | | 7,7 | 7,6 | | 7,7 | 7,6 |
| STAG2 | 7,2 | 7,3 | | 7,0 | 7,4 | | 7,5 | 7,5 |
| TGFB1 | 3,5 | 3,0 | | 2,9 | 3,9 | | 3,4 | 3,8 |
| TGFB1I1 | 4,1 | 4,0 | | 4,5 | 4,2 | | 4,2 | 4,3 |
| TGFB2 | 4,3 | 4,7 | | 4,1 | 4,6 | | 4,0 | 4,5 |
| TGFBI | 5,1 | 5,1 | | 4,9 | 5,1 | | 5,2 | 5,3 |
| TGFBR2 | 3,9 | 3,7 | | 3,4 | 3,4 | | 3,4 | 3,7 |
| TGFBRAP1 | 6,4 | 6,2 | | 6,2 | 6,9 | | 6,9 | 6,9 |
| TP53 | 4,7 | 5,0 | | 5,2 | 5,2 | | 5,0 | 5,5 |
| TRIM21 | 6,4 | 6,5 | | 6,2 | 6,6 | | 6,4 | 6,5 |
| VIM | 10,7 | 10,6 | | 10,8 | 10,9 | | 10,8 | 10,9 |

# Supplementary Table 7

|  | | | **MDA-MB-231** | | | **MDA-MB-231(NPC)** | | | |
| --- | --- | --- | --- | --- | --- | --- | --- | --- | --- |
| **Gene symbol** | **Sample 1** | **Sample 2** | | **Sample 3** | **Sample 1** | | **Sample 2** | **Sample 3** |  |
| ABI1 | 6,0 | 5,7 | | 5,8 | 5,9 | | 5,9 | 6,0 |  |
| AHNAK | 13,8 | 13,7 | | 13,7 | 14,7 | | 14,7 | 14,7 |  |
| AHNAK2 | 9,2 | 9,2 | | 9,2 | 10,7 | | 10,7 | 10,7 |  |
| AKT1 | 7,8 | 7,8 | | 7,9 | 8,3 | | 8,4 | 8,4 |  |
| ARHGAP35 | 7,4 | 7,5 | | 7,6 | 7,8 | | 7,8 | 7,8 |  |
| BRAF | 3,6 | 3,6 | | 3,8 | 3,2 | | 3,4 | 3,4 |  |
| BRCA1 | 3,5 | 3,5 | | 3,3 | 2,7 | | 2,9 | 2,4 |  |
| BRCA2 | 3,6 | 3,4 | | 3,2 | 3,5 | | 3,5 | 3,1 |  |
| CALD1 | 8,4 | 8,5 | | 8,4 | 8,4 | | 8,4 | 8,3 |  |
| CASP8 | 7,6 | 7,5 | | 7,5 | 8,0 | | 8,0 | 8,0 |  |
| CDC42 | 4,7 | 4,2 | | 4,3 | 4,2 | | 3,8 | 3,9 |  |
| CDH1 | 5,6 | 5,4 | | 5,6 | 5,4 | | 5,4 | 5,8 |  |
| CDH11 | 6,5 | 6,3 | | 6,5 | 7,5 | | 7,4 | 7,5 |  |
| CLPP | 8,0 | 8,1 | | 8,0 | 7,6 | | 7,7 | 7,6 |  |
| CTNNA2 | 7,5 | 7,2 | | 7,4 | 7,3 | | 7,4 | 7,3 |  |
| CTNNB1 | 6,3 | 6,3 | | 6,4 | 6,6 | | 6,7 | 6,8 |  |
| EGFR | 8,0 | 7,7 | | 7,7 | 7,4 | | 7,3 | 7,2 |  |
| ESR1 | 3,4 | 3,3 | | 3,3 | 3,3 | | 3,5 | 3,6 |  |
| FASN | 10,9 | 10,8 | | 10,9 | 12,2 | | 12,1 | 12,1 |  |
| FGFR1 | 2,8 | 3,2 | | 3,0 | 3,2 | | 3,5 | 3,4 |  |
| FGFR1OP | 4,9 | 5,2 | | 4,8 | 5,0 | | 4,8 | 4,8 |  |
| FGFR1OP2 | 5,0 | 4,7 | | 4,8 | 4,7 | | 4,9 | 4,9 |  |
| KRAS | 5,0 | 5,0 | | 5,1 | 5,1 | | 5,4 | 4,9 |  |
| MAP2K4 | 6,4 | 6,3 | | 6,3 | 7,0 | | 7,0 | 7,1 |  |
| MAPT | 4,0 | 3,8 | | 3,8 | 4,8 | | 4,4 | 4,4 |  |
| MKI67 | 8,4 | 8,3 | | 8,3 | 8,3 | | 8,2 | 8,3 |  |
| MTOR | 7,4 | 7,4 | | 7,6 | 7,8 | | 7,8 | 7,9 |  |
| MUC1 | 6,1 | 6,0 | | 6,1 | 7,8 | | 7,9 | 8,0 |  |
| MUC5B | 5,8 | 5,8 | | 5,8 | 7,7 | | 7,8 | 7,9 |  |
| NRAS | 5,9 | 5,5 | | 5,7 | 5,8 | | 5,7 | 5,5 |  |
| NUP93 | 8,7 | 8,7 | | 8,7 | 8,5 | | 8,4 | 8,4 |  |
| PAK4 | 7,5 | 7,4 | | 7,5 | 7,7 | | 7,4 | 7,6 |  |
| PKP2 | 6,7 | 6,5 | | 6,6 | 7,3 | | 7,2 | 7,2 |  |
| PTEN | 6,2 | 6,0 | | 6,1 | 6,4 | | 6,6 | 6,3 |  |
| PTPN11 | 8,0 | 8,0 | | 8,0 | 8,4 | | 8,4 | 8,3 |  |
| PXN | 7,2 | 7,2 | | 7,1 | 7,4 | | 7,6 | 7,5 |  |
| RAC1 | 6,0 | 6,2 | | 6,1 | 6,4 | | 6,4 | 6,3 |  |
| RB1 | 8,5 | 8,5 | | 8,5 | 7,8 | | 7,8 | 7,8 |  |
| RHOA | 4,3 | 4,6 | | 4,6 | 4,7 | | 5,0 | 4,8 |  |
| RIPK2 | 3,9 | 3,6 | | 4,1 | 3,7 | | 4,0 | 4,0 |  |
| ROBO1 231 | 2,6 | 1,8 | | 2,7 | 3,2 | | 2,5 | 2,4 |  |
| ROCK1 | 7,4 | 7,5 | | 7,5 | 7,3 | | 7,3 | 7,4 |  |
| STAG2 | 7,1 | 6,9 | | 7,1 | 6,7 | | 6,8 | 6,7 |  |
| TGFB1 | 4,3 | 3,9 | | 4,3 | 3,9 | | 4,0 | 4,3 |  |
| TGFB1I1 | 6,2 | 6,2 | | 6,2 | 6,5 | | 6,5 | 6,6 |  |
| TGFB2 | 5,6 | 5,4 | | 5,7 | 5,1 | | 5,0 | 4,7 |  |
| TGFBI | 5,9 | 5,8 | | 5,8 | 4,9 | | 4,3 | 4,4 |  |
| TGFBR2 | 3,9 | 3,9 | | 3,8 | 6,2 | | 6,2 | 6,0 |  |
| TGFBRAP1 | 6,4 | 6,3 | | 6,2 | 6,3 | | 6,3 | 6,3 |  |
| TP53 | 8,6 | 8,4 | | 8,4 | 7,5 | | 7,6 | 7,5 |  |
| TRIM21 | 6,7 | 7,0 | | 6,9 | 7,3 | | 7,3 | 7,0 |  |
| VIM | 13,3 | 13,4 | | 13,4 | 12,8 | | 12,8 | 12,8 |  |

# Supplementary Table 8

|  | **NPC** | | | | | **NPC(MCF7)** | | | **NPC(231)** | |
| --- | --- | --- | --- | --- | --- | --- | --- | --- | --- | --- |
| **Gene Symbol** | **Sample1** | | **Sample2** | | **Sample1** | | | **Sample1** | |  |
| AASDHPPT | 7.372 | | 17.350 | | 19.589 | | | 6.246 | |  |
| ABI2 | 17.616 | | 17.474 | | 17.312 | | | 18.114 | |  |
| ACAT2 | 18.826 | | 18.881 | | 17.513 | | | 17.671 | |  |
| ACLY | 11.076 | | 11.032 | | 10.309 | | | 10.416 | |  |
| ACTA1 | 13.242 | | 13.292 | | 11.655 | | | 11.872 | |  |
| ACTB | 16.778 | | 16.730 | | 18.509 | | | 18.017 | |  |
| ACTL6A | 18.880 | | 18.941 | | 16.994 | | | 16.856 | |  |
| ALDH7A1 | 19.036 | | 19.079 | | 18.571 | | | 18.512 | |  |
| ANP32E | 19.349 | | 19.339 | | 17.345 | | | 6.991 | |  |
| APEH | 18.169 | | 18.191 | | 17.399 | | | 17.487 | |  |
| APEX1 | 19.852 | | 10.006 | | 18.408 | | | 18.213 | |  |
| API5 | 19.351 | | 9.363 | | 17.998 | | | 17.688 | |  |
| ARPC2 | 10.284 | | 10.237 | | 18.479 | | | 18.726 | |  |
| ARPC4 | 18.403 | | 18.459 | | 19.637 | | | 16.701 | |  |
| ASF1A | 17.087 | | 16.980 | | 18.873 | | | 18.960 | |  |
| ATIC | 10.178 | | 10.112 | | 19.476 | | | 19.444 | |  |
| ATL1 | 18.334 | | 18.365 | | 15.499 | | | 18.782 | |  |
| BAG6 | 19.821 | | 19.875 | | 18.013 | | | 15.086 | |  |
| BASP1 | 17.578 | | 17.515 | | 15.680 | | | 19.230 | |  |
| BUB3 | 18.508 | | 18.566 | | 17.050 | | | 16.886 | |  |
| CACYBP | 10.639 | | 10.576 | | 19.020 | | | 19.285 | |  |
| CAP2 | 7.804 | | 17.697 | | 19.480 | | | 19.495 | |  |
| CASP3 | 17.179 | | 17.167 | | 18.521 | | | 18.511 | |  |
| CCNK | 19.876 | | 16.654 | | 17.217 | | | 17.230 | |  |
| CCT3 | 11.025 | | 11.061 | | 19.703 | | | 19.531 | |  |
| CCT6A | 10.067 | | 10.105 | | 19.258 | | | 19.136 | |  |
| CCT8 | 10.880 | | 10.818 | | 10.054 | | | 10.056 | |  |
| CDK1 | 18.975 | | 18.985 | | 17.516 | | | 17.695 | |  |
| CFDP1 | 17.674 | | 17.714 | | 16.843 | | | 16.900 | |  |
| CGAS | 16.806 | | 17.129 | | 18.814 | | | 18.896 | |  |
| CHD4 | 19.278 | | 9.450 | | 17.832 | | | 17.790 | |  |
| COPA | 18.466 | | 18.444 | | 17.959 | | | 17.940 | |  |
| COTL1 | 8.661 | | 18.580 | | 16.997 | | | 6.747 | |  |
| CPSF6 | 18.145 | | 18.271 | | 19.704 | | | 19.739 | |  |
| CRABP1 | 19.322 | | 19.396 | | 17.415 | | | 17.503 | |  |
| CRKL | 19.008 | | 19.007 | | 8.284 | | | 18.286 | |  |
| CSNK2A3 | 18.581 | | 18.453 | | 19.014 | | | 18.518 | |  |
| CTNNBL1 | 19.197 | | 19.162 | | 17.656 | | | 17.886 | |  |
| CUL7 | 18.199 | | 18.276 | | 17.338 | | | 14.114 | |  |
| DBN1 | 10.443 | | 10.427 | | 18.965 | | | 18.746 | |  |
| DBR1 | 18.848 | | 18.873 | | 19.211 | | | 19.607 | |  |
| DCTN2 | 18.588 | | 18.617 | | 17.613 | | | 17.445 | |  |
| DCX | 11.171 | | 11.118 | | 18.606 | | | 18.474 | |  |
| DDAH1 | 11.456 | | 11.497 | | 17.531 | | | 18.564 | |  |
| DDX1 | 18.308 | | 18.160 | | 19.631 | | | 19.695 | |  |
| DDX39B | 11.277 | | 11.233 | | 19.695 | | | 19.870 | |  |
| DHFR | 18.260 | | 18.329 | | 17.179 | | | 17.233 | |  |
| DMKN | 16.891 | | 17.017 | | 18.647 | | | 18.599 | |  |
| DNAJB1 | 18.806 | | 18.809 | | 19.060 | | | 19.739 | |  |
| DPP3 | 9.326 | | 19.317 | | 18.396 | | | 8.539 | |  |
| DPYSL3 | 11.573 | | 11.631 | | 10.129 | | | 10.127 | |  |
| DPYSL4 | 19.611 | | 19.713 | | 17.801 | | | 17.402 | |  |
| DSTN | 9.396 | | 9.352 | | 19.734 | | | 9.775 | |  |
| DYNC1H1 | 12.270 | | 12.264 | | 11.723 | | | 11.799 | |  |
| DYNLL2 | 19.714 | | 19.707 | | 16.989 | | | 19.134 | |  |
| EEF2 | 12.581 | | 12.524 | | 11.627 | | | 11.462 | |  |
| EFHD2 | 14.677 | | 17.979 | | 18.371 | | | 15.063 | |  |
| EFTUD2 | 19.750 | | 19.728 | | 18.743 | | | 18.871 | |  |
| EIF2A | 18.902 | | 18.974 | | 18.131 | | | 18.195 | |  |
| EIF2S3 | 9.999 | | 9.868 | | 18.247 | | | 18.278 | |  |
| EIF3E | 19.445 | | 19.505 | | 18.499 | | | 18.451 | |  |
| EIF3F | 18.516 | | 18.459 | | 16.960 | | | 16.517 | |  |
| EIF4A2 | 10.607 | | 10.575 | | 18.679 | | | 19.027 | |  |
| EIF5 | 8.115 | | 17.992 | | 19.251 | | | 19.578 | |  |
| ELOB | 18.631 | | 18.653 | | 19.718 | | | 16.742 | |  |
| ENSA | 18.104 | | 8.114 | | 7.754 | | | 17.750 | |  |
| EPS15L1 | 9.049 | | 9.066 | | 17.672 | | | 17.846 | |  |
| EXOSC9 | 17.155 | | 17.099 | | 19.419 | | | 19.217 | |  |
| FABP5 | 11.117 | | 11.118 | | 19.458 | | | 9.285 | |  |
| FAM192A | 19.238 | | 19.163 | | 18.068 | | | 18.231 | |  |
| FIS1 | 17.695 | | 17.621 | | 18.197 | | | 18.855 | |  |
| G3BP1 | 18.097 | | 17.987 | | 16.822 | | | 16.737 | |  |
| GDI1 | 11.577 | | 11.588 | | 10.401 | | | 10.200 | |  |
| GDI2 | 11.656 | | 11.686 | | 10.396 | | | 10.499 | |  |
| GNAO1 | 17.402 | | 17.469 | | 18.650 | | | 18.562 | |  |
| GPC4 | 17.511 | | 7.588 | | 16.042 | | | 19.192 | |  |
| GPHN | 18.846 | | 18.739 | | 16.903 | | | 16.729 | |  |
| GRHPR | 16.615 | | 19.930 | | 15.512 | | | 18.668 | |  |
| GTF2I | 19.102 | | 19.038 | | 17.514 | | | 17.492 | |  |
| H2AZ1 | 18.735 | | 18.694 | | 19.540 | | | 18.792 | |  |
| HDAC2 | 19.482 | | 19.487 | | 18.175 | | | 17.949 | |  |
| HEBP2 | 16.906 | | 16.919 | | 17.386 | | | 7.409 | |  |
| HMGB2 | 8.585 | | 8.623 | | 17.055 | | | 16.816 | |  |
| HNRNPK | 13.131 | | 13.173 | | 11.022 | | | 11.369 | |  |
| HPRT1 | 19.081 | | 18.951 | | 17.472 | | | 17.366 | |  |
| HSPA1A | 11.426 | | 11.383 | | 10.965 | | | 10.979 | |  |
| IFRD1 | 19.629 | | 19.827 | | 18.981 | | | 19.223 | |  |
| IGF2BP1 | 19.516 | | 9.570 | | 7.319 | | | 19.763 | |  |
| IGF2BP3 | 18.826 | | 18.896 | | 17.202 | | | 16.841 | |  |
| ILF2 | 11.581 | | 11.633 | | 10.127 | | | 9.923 | |  |
| INA | 10.026 | | 10.007 | | 7.964 | | | 17.097 | |  |
| INTS13 | 15.858 | | 19.118 | | 18.074 | | | 18.104 | |  |
| INTS3 | 19.695 | | 19.697 | | 17.525 | | | 17.769 | |  |
| ISYNA1 | 19.511 | | 19.488 | | 17.497 | | | 19.916 | |  |
| KARS1 | 19.725 | | 19.681 | | 18.271 | | | 8.059 | |  |
| KHDRBS1 | 11.090 | | 11.152 | | 19.341 | | | 18.992 | |  |
| KRT18 | 11.956 | | 12.134 | | 13.220 | | | 13.306 | |  |
| KRT8 | 12.615 | | 12.590 | | 13.990 | | | 13.967 | |  |
| KYNU | 19.918 | | 16.678 | | 17.188 | | | 17.154 | |  |
| LAMB1 | 17.662 | | 17.597 | | 16.742 | | | 16.632 | |  |
| LARS | 18.657 | | 18.676 | | 17.478 | | | 17.524 | |  |
| LDHB | 11.159 | | 11.108 | | 19.446 | | | 19.286 | |  |
| LRRC40 | 16.894 | | 16.846 | | 17.877 | | | 18.305 | |  |
| LRRC57 | 18.747 | | 18.688 | | 19.619 | | | 19.418 | |  |
| LSM7 | 19.557 | | 19.570 | | 17.209 | | | 17.679 | |  |
| MAP1A | 8.511 | | 18.611 | | 17.986 | | | 17.775 | |  |
| MAP1S | 18.078 | | 18.027 | | 16.989 | | | 16.744 | |  |
| MAPRE1 | 9.974 | | 9.984 | | 18.861 | | | 18.914 | |  |
| MARCKS | 10.483 | | 10.442 | | 17.148 | | | 17.480 | |  |
| MCM3 | 19.775 | | 19.755 | | 18.205 | | | 18.039 | |  |
| MDH1 | 19.114 | | 19.067 | | 17.158 | | | 17.498 | |  |
| ME2 | 18.668 | | 18.649 | | 17.147 | | | 17.290 | |  |
| MEMO1 | 18.949 | | 18.966 | | 18.319 | | | 18.210 | |  |
| MRPL19 | 16.942 | | 16.914 | | 17.537 | | | 17.514 | |  |
| MRPS23 | 18.917 | | 18.876 | | 16.973 | | | 17.098 | |  |
| MSI1 | 18.119 | | 18.037 | | 18.911 | | | 19.231 | |  |
| MSI2 | 16.175 | | 16.152 | | 16.651 | | | 16.696 | |  |
| MVK | 18.086 | | 18.040 | | 16.872 | | | 16.658 | |  |
| MYEF2 | 19.910 | | 19.916 | | 14.304 | | | 17.316 | |  |
| NAPA | 18.275 | | 18.263 | | 17.486 | | | 17.328 | |  |
| NCBP1 | 10.412 | | 10.360 | | 18.482 | | | 18.563 | |  |
| NLN | 17.898 | | 17.810 | | 16.756 | | | 16.816 | |  |
| NONO | 19.231 | | 19.280 | | 8.065 | | | 18.193 | |  |
| NTNG1 | 17.351 | | 17.225 | | 17.466 | | | 19.605 | |  |
| NUDT21 | 19.721 | | 19.698 | | 17.781 | | | 18.125 | |  |
| NUP93 | 18.962 | | 18.956 | | 17.612 | | | 17.712 | |  |
| NUTF2 | 17.786 | | 17.704 | | 19.798 | | | 19.567 | |  |
| NXN | 19.413 | | 19.504 | | 16.821 | | | 17.403 | |  |
| OLA1 | 10.098 | | 10.167 | | 19.183 | | | 19.136 | |  |
| PA2G4 | 10.590 | | 10.601 | | 19.225 | | | 19.224 | |  |
| PAPSS1 | 18.773 | | 18.751 | | 14.328 | | | 17.417 | |  |
| PARK7 | 10.080 | | 10.053 | | 19.517 | | | 19.447 | |  |
| PARP1 | 10.920 | | 11.039 | | 19.593 | | | 19.460 | |  |
| PEA15 | 17.126 | | 17.178 | | 19.712 | | | 6.296 | |  |
| PFDN4 | 16.641 | | 16.661 | | 17.675 | | | 17.701 | |  |
| PFDN5 | 16.797 | | 16.886 | | 18.380 | | | 18.587 | |  |
| PFKM | 17.310 | | 17.347 | | 19.256 | | | 19.008 | |  |
| PGAM5 | 17.437 | | 17.401 | | 19.458 | | | 19.697 | |  |
| PGD | 10.762 | | 10.715 | | 19.460 | | | 9.205 | |  |
| PHKB | 19.412 | | 19.318 | | 17.009 | | | 6.346 | |  |
| PIP4K2A | 16.863 | | 16.843 | | 18.436 | | | 18.437 | |  |
| PITPNB | 18.200 | | 8.247 | | 16.845 | | | 16.668 | |  |
| PKM | 12.718 | | 12.724 | | 12.030 | | | 12.115 | |  |
| PNP | 17.517 | | 17.460 | | 16.733 | | | 16.703 | |  |
| POLR3C | 19.278 | | 19.246 | | 17.867 | | | 18.035 | |  |
| POTEF | 11.680 | | 11.692 | | 10.623 | | | 10.620 | |  |
| PPA1 | 10.763 | | 10.786 | | 19.892 | | | 19.887 | |  |
| PPID | 17.343 | | 17.244 | | 19.113 | | | 18.800 | |  |
| PPM1G | 18.168 | | 8.182 | | 16.795 | | | 16.828 | |  |
| PPP1CA | 10.259 | | 10.192 | | 18.679 | | | 18.745 | |  |
| PPP1R18 | 19.899 | | 16.653 | | 17.782 | | | 17.807 | |  |
| PPP1R7 | 18.963 | | 18.933 | | 16.913 | | | 17.160 | |  |
| PPP1R9A | 18.747 | | 18.668 | | 19.898 | | | 19.864 | |  |
| PPP2R1A | 10.547 | | 10.484 | | 19.655 | | | 19.593 | |  |
| PPP2R5D | 19.605 | | 19.579 | | 14.308 | | | 17.807 | |  |
| PPP6C | 16.948 | | 16.980 | | 18.079 | | | 18.299 | |  |
| PPWD1 | 18.116 | | 18.133 | | 16.706 | | | 16.854 | |  |
| PRDX2 | 11.307 | | 11.385 | | 9.790 | | | 9.893 | |  |
| PRDX5 | 10.782 | | 10.781 | | 9.782 | | | 19.774 | |  |
| PRKDC | 10.855 | | 10.847 | | 10.464 | | | 10.413 | |  |
| PSAT1 | 10.039 | | 10.046 | | 17.954 | | | 17.889 | |  |
| PSIP1 | 7.773 | | 17.790 | | 18.523 | | | 18.408 | |  |
| PSMA7 | 18.571 | | 18.656 | | 17.534 | | | 17.497 | |  |
| PSMC4 | 19.067 | | 19.025 | | 18.276 | | | 18.144 | |  |
| PSMD1 | 18.563 | | 18.552 | | 18.133 | | | 18.145 | |  |
| PSMD10 | 19.625 | | 19.575 | | 17.552 | | | 17.576 | |  |
| PSMD12 | 10.140 | | 10.170 | | 17.896 | | | 18.075 | |  |
| PSMD13 | 19.868 | | 19.898 | | 17.853 | | | 18.251 | |  |
| PSMD2 | 19.024 | | 9.080 | | 18.820 | | | 18.795 | |  |
| PSMD8 | 17.970 | | 18.018 | | 19.498 | | | 18.996 | |  |
| PTK2 | 15.872 | | 19.114 | | 17.803 | | | 17.711 | |  |
| PTMS | 17.625 | | 17.568 | | 19.284 | | | 5.599 | |  |
| PTPN11 | 18.257 | | 18.247 | | 17.409 | | | 17.421 | |  |
| RABGEF1 | 18.778 | | 18.782 | | 16.695 | | | 17.039 | |  |
| RAN | 10.242 | | 10.242 | | 19.007 | | | 18.825 | |  |
| RANBP1 | 18.838 | | 18.889 | | 17.789 | | | 17.741 | |  |
| RANBP3 | 18.882 | | 18.970 | | 7.595 | | | 17.489 | |  |
| RB1 | 16.626 | | 19.814 | | 18.041 | | | 17.942 | |  |
| RBBP7 | 18.174 | | 18.190 | | 16.898 | | | 17.095 | |  |
| RBM10 | 15.878 | | 19.162 | | 18.197 | | | 18.270 | |  |
| RBM4 | 19.076 | | 19.113 | | 17.234 | | | 17.533 | |  |
| RCC2 | 10.608 | | 10.675 | | 18.737 | | | 18.599 | |  |
| RECQL | 17.649 | | 17.645 | | 19.425 | | | 19.175 | |  |
| RIF1 | 19.446 | | 19.428 | | 18.378 | | | 18.271 | |  |
| RINT1 | 19.076 | | 19.166 | | 17.971 | | | 17.862 | |  |
| RNMT | 18.911 | | 18.813 | | 16.987 | | | 16.887 | |  |
| RO60 | 18.460 | | 18.429 | | 7.062 | | | 16.755 | |  |
| RPLP1 | 18.636 | | 8.704 | | 17.024 | | | 16.628 | |  |
| RPS15A | 18.612 | | 18.693 | | 7.384 | | | 17.480 | |  |
| RPSA | 11.656 | | 11.607 | | 10.248 | | | 19.906 | |  |
| RUFY3 | 17.811 | | 17.850 | | 18.651 | | | 18.583 | |  |
| SAR1B | 7.613 | | 17.610 | | 18.844 | | | 18.931 | |  |
| SARS | 10.732 | | 10.680 | | 19.162 | | | 19.186 | |  |
| SCRIB | 18.840 | | 18.821 | | 18.120 | | | 18.207 | |  |
| SELENOH | 16.611 | | 16.678 | | 18.155 | | | 15.203 | |  |
| SEPTIN11 | 10.015 | | 9.997 | | 8.497 | | | 18.666 | |  |
| SET | 11.126 | | 11.080 | | 18.967 | | | 19.349 | |  |
| SMC3 | 19.258 | | 19.356 | | 18.010 | | | 17.909 | |  |
| SNRNP200 | 11.222 | | 11.185 | | 19.630 | | | 19.797 | |  |
| SNRNP40 | 18.391 | | 18.331 | | 17.125 | | | 17.260 | |  |
| SNX12 | 19.210 | | 19.153 | | 17.968 | | | 18.013 | |  |
| SPC24 | 16.794 | | 16.673 | | 14.031 | | | 16.759 | |  |
| SPG11 | 17.204 | | 17.149 | | 18.691 | | | 14.932 | |  |
| SRP9 | 19.450 | | 19.559 | | 18.110 | | | 18.032 | |  |
| SRRM1 | 16.925 | | 16.871 | | 19.015 | | | 19.035 | |  |
| SRSF6 | 18.018 | | 17.979 | | 17.111 | | | 17.177 | |  |
| SSB | 11.671 | | 11.604 | | 10.985 | | | 10.932 | |  |
| SSBP3 | 17.631 | | 17.608 | | 16.213 | | | 19.297 | |  |
| ST13 | 11.371 | | 11.438 | | 9.970 | | | 19.756 | |  |
| STMN1 | 11.764 | | 11.746 | | 19.862 | | | 10.267 | |  |
| SUB1 | 11.116 | | 11.170 | | 19.624 | | | 9.875 | |  |
| SUGP2 | 19.504 | | 19.517 | | 18.700 | | | 18.545 | |  |
| SUPT16H | 10.287 | | 10.328 | | 18.802 | | | 18.753 | |  |
| TBCB | 11.086 | | 11.149 | | 17.589 | | | 18.511 | |  |
| TCP1 | 10.624 | | 10.661 | | 10.056 | | | 10.038 | |  |
| TNPO1 | 19.690 | | 19.729 | | 18.187 | | | 18.436 | |  |
| TRNT1 | 7.112 | | 17.083 | | 18.801 | | | 18.515 | |  |
| TSN | 10.152 | | 10.147 | | 17.990 | | | 18.205 | |  |
| TUBA1A | 14.235 | | 14.241 | | 12.394 | | | 12.554 | |  |
| TUBB | 10.573 | | 10.575 | | 19.074 | | | 19.358 | |  |
| TUBB1 | 12.482 | | 12.610 | | 10.516 | | | 10.753 | |  |
| TUBB2A | 11.885 | | 11.913 | | 10.256 | | | 10.152 | |  |
| TUBB2B | 19.520 | | 9.682 | | 16.652 | | | 16.975 | |  |
| TXLNA | 17.618 | | 17.559 | | 16.384 | | | 19.748 | |  |
| UBLCP1 | 17.530 | | 17.377 | | 17.927 | | | 18.108 | |  |
| UMPS | 19.508 | | 19.598 | | 18.637 | | | 18.661 | |  |
| USP39 | 17.030 | | 17.021 | | 18.363 | | | 18.335 | |  |
| USP5 | 8.855 | | 18.817 | | 17.587 | | | 17.253 | |  |
| UTRN | 19.640 | | 19.665 | | 17.459 | | | 17.352 | |  |
| VAMP3 | 16.933 | | 16.774 | | 17.979 | | | 14.700 | |  |
| VPS26A | 19.549 | | 19.567 | | 18.082 | | | 18.182 | |  |
| XPO1 | 18.722 | | 18.686 | | 7.455 | | | 17.523 | |  |
| XRCC5 | 12.268 | | 12.346 | | 10.437 | | | 10.823 | |  |
| XRCC6 | 11.730 | | 11.776 | | 9.613 | | | 19.531 | |  |
| YARS | 18.551 | | 18.627 | | 17.673 | | | 17.567 | |  |
|  |  |  | |  | | |  | | |  |
