# Supplementary material for: Global and single-cell proteomics view of the co-evolution between neural progenitors and breast cancer cells in a co-culture model
Source: eBioMedicine. 2024 Sep 4;108:105325. doi: 10.1016/j.ebiom.2024.105325 (PMC11404160; doi:10.1016/j.ebiom.2024.105325)

Thaw and Culture Details

Cell Line Name	**WA09**
WiCell Lot Number	**WB66446**
Parent Material	WA09-MCB-01
Provider	University of Wisconsin – Dr. James Thomson
Banked By	WiCell
Thaw and Culture Recommendations	WiCell recommends thawing 1 vial into 1 well of a 6 well plate. WiCell recommends only collagenase passaging.
Culture Platform	Feeder Dependent
	Medium: hESC Medium (KOSR)
	Matrix: MEF
Protocol	WiCell Feeder Dependent Protocol
Passage Number	p28<br>These cells were cultured for 27 passages prior to freeze and post reprogramming. WiCell adds +1 to the passage number to best represent the overall passage number of the cells at thaw.
Date Vialed	20-July-2017
Vial Label	WA09<br>p28<br>WB66446
Biosafety and Use Information	Appropriate biosafety precautions should be followed when working with these cells. The end user is responsible for ensuring that the cells are handled and stored in an appropriate manner. WiCell is not responsible for damages or injuries that may result from the use of these cells.<br>Cells distributed by WiCell are intended for research purposes only and are not intended for use in humans.

Testing Performed by WiCell

Test Description	Test Provider	Test Method	Test Specification	Result
Karyotype by G-banding	WiCell	SOP-CH-003	Expected karyotype	Pass
Post-Thaw Viable Cell Recovery	WiCell	SOP-CH-305	≥ 15 Undifferentiated Colonies, ≤ 30% Differentiation and recoverable attachment after passage<br>Recoverable attachment after passage	Pass
Identity by STR	UW Translational Research Initiatives in Pathology Laboratory	PowerPlex 16 HS System by Promega	Consistent with known profile	Pass
Sterility	Biotest Laboratories	ST/07	Negative	Pass
Mycoplasma	WiCell	SOP-QU-004	Negative	Pass

Approval Date	Quality Assurance Approval
28-August-2017	9/6/2017<br>X AMK<br>AMK<br>Quality Assurance<br>Signed by: Klade, Anjelica

©2017 WiCell Research Institute
The material provided under this certificate has been subjected to the tests specified and the results and data described herein are accurate based on WiCell's reasonable knowledge and belief. Appropriate Biosafety Level practices and universal precautions should always be used with this material. For clarity, the foregoing is governed solely by WiCell's Terms and Conditions of Service, which can be found at http://www.wicell.org/privacyandterms.

Chromosome Analysis Report: 067448

Date Reported: Friday, August 18, 2017
Cell Line: WA09-WB66446 12683
Passage#: 29
Date of Sample: 8/8/2017
Specimen: Human ESC
Results: 46,XX

Cell Line Gender: Female
Reason for Testing: lot release testing

Investigator: [redacted], WiCell CDM

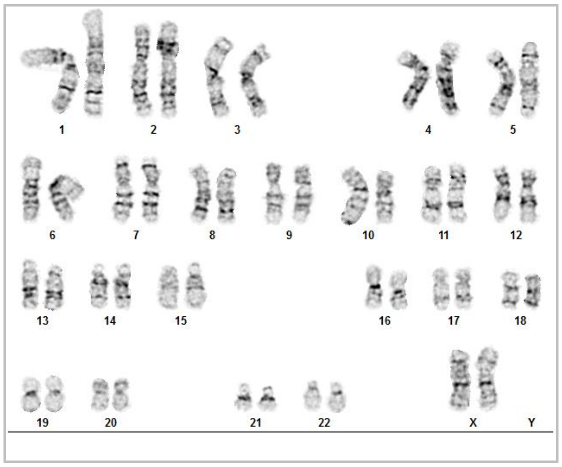

Cell: 34
Slide: G03
Slide Type: Karyotype

Total Counted: 20
Total Analyzed: 8
Total Karyogrammed: 4
Band Resolution: 425 - 475

Interpretation:

This is a normal karyotype. No clonal abnormalities were detected at the stated band level of resolution.

Completed by: [redacted], CG(ASCP)
Reviewed and Interpreted by: [redacted], PhD, FACMG
A signed copy of this report is available upon request.

Date:________________________ **Sent By:**____ **Sent To:**______________________ **QC Review By:** ____

Limitations: This assay allows for microscopic visualization of numerical and structural chromosome abnormalities. The size of structural abnormality that can be detected is >3-10Mb, dependent upon the G-band resolution obtained from this specimen. For the purposes of this report, band level is defined as the number of G-bands per haploid genome. It is documented here as "band level", i.e., the range of bands determined from the four karyograms in this assay. Detection of heterogeneity of clonal cell populations in this specimen (i.e.,mosaicism) is limited by the number of metaphase cells examined, documented here as "# of cells counted".

This assay was conducted solely for listed investigator/institution. The results may not be relied upon by any other party without the prior written consent of the Director of the WiCell Cytogenetics Laboratory. The resu lts of this assay are for research use only. If the results of this assay are to be used for any other purpose, contact the Director of the WiCell Cytogenetics Laboratory.

Unless otherwise mutually agreed in writing, the services provided to you hereunder by WiCell Research Institute, Inc. ("WiCell") are governed solely by WiCell's Terms and Conditions of Service, found at www.wicell.org/privacyandterms. Any terms you may attach to a purchase order or other document that are inconsistent, add to, or conflict with WiCell's Terms and Conditions of Service are null and void and of no legal force or effect.

Short Tandem Repeat Analysis

School of Medicine and Public Health
UNIVERSITY OF WISCONSIN-MADISON
Department of Pathology and Laboratory Medicine
TRIP Laboratory (Molecular)
http://www.pathology.wisc.edu/research/trip

WiCell®
info@wicell.org
(888) 204-1782

Sample Report:
12683-STR
Sample Name on Tube: 12683-STR
47.9 ng/µL, (A260/280=2.03)
Sample Type: Cells
Cell Count: ~2 million cells

Requestor:
WiCell Research Institute
Quality Department

Sample Date: N/A
Receive Date: 08/14/17
Assay Date: 08/16/17
File Name: STR 170817 wmr
Report Date: 08/21/17

STR Locus	STR Genotype Repeat #	STR Genotype
FGA	16–18,18.2,19,19.2,20,20.2,21,21.2,22, 22.2, 23, 23.2, 24, 24.2, 25, 25.2, 26–30, 31.2, 43.2, 44.2,45.2, 46.2	26,28
TPOX	6-13	10,11
D8S1179	7-18	8,14
vWA	10-22	17,17
Amelogenin	X,Y	X,X
Penta_D	2.2, 3.2, 5, 7-17	9,13
CSF1PO	6-15	11,11
D16S539	5, 8-15	12,13
D7S820	6-14	9,11
D13S317	7-15	9,9
D5S818	7-16	11,12
Penta_E	5-24	11,14
D18S51	8-10, 10.2, 11-13, 13.2, 14-27	13,13
D21S11	24,24.2,25,25.2,26-28,28.2,29,29.2, 30, 30.2,31, 31.2,32,32.2,33,33.2, 34,34.2,35,35.2,36-38	30,30
TH01	4-9,9.3,10-11,13.3	9.3,9.3
D3S1358	12-20	13,16

Results: **Based on the 12683-STR cells submitted by WiCell QA dated and received on 08/14/17, this sample (Label on Tube: 12683-STR) exactly matches the STR profile of the human stem cell line WA09 comprising 24 allelic polymorphisms across the 15 STR loci analyzed.**

Interpretation: **No STR polymorphisms other than those corresponding to the human WA09 stem cell line were detected and the concentration of DNA required to achieve an acceptable STR genotype (signal/ noise) was equivalent to that required for the standard procedure (~1 ng/amplification reaction) from human genomic DNA. This result suggests that the 12683-STR sample submitted corresponds to the WA09 stem cell line and was not contaminated with any other human stem cells or a significant amount of mouse feeder layer cells.**

Sensitivity: **Sensitivity limits for detection of STR polymorphisms unique to either this or other human stem cell lines is ~2-5%.**

X *RMB* **Digitally Signed on 08/23/17**

[redacted]
TRIP Laboratory, Molecular

X *WMR* **Digitally Signed on 08/23/17**

[redacted]*, PhD, Director / Co-Director*
UWHC Molecular Diagnostics Laboratory / UWSMPH TRIP Laboratory

Testing was accomplished by analysis of human genetic polymorphisms at STR loci. This methodology has not yet been approved by the FDA and is for investigational use only.
Acknowledge TRIP in your publications, posters & presentations. For details, see: *http://www.pathology.wisc.edu/research/trip/acknowledging*
TRIP agrees to maintain the confidentiality of any information provided to it in connection with its performance of this STR analysis on the same conditions as set forth in paragraph 2 of WiCell's Terms and Conditions of Service (http://www.wicell.org/media.acux/1a429b84-2b54-44a4-8ad8-5c05db93dd8a).

Native Product Sterility Report

STERIS

SAMPLE #:	17071725
DATE RECEIVED:	27-Jul-17
TEST INITIATED:	31-Jul-17
TEST COMPLETED:	14-Aug-17

WiCell
504 S Rosa Rd, Rm 101
Madison, WI 53719

SAMPLE NAME / DESCRIPTION:
HVRDi001-A-WB66391 12659
JHU024i-WB66445 12660
WA09-WB66444 12661
WA09-WB66446 12662
UCSD005i-43-1-WB62267 12663
UCSD008i-44-1-WB66286 12664
UCSD036i-4-5-WB65173 12665
UCSD233i-SAD-2-DB26810 12666
UCSD235i-SAD2-4-DB26816 12667
iPS(IMR90)-2-WB66447 12668

UNIQUE IDENTIFIER: NA

PRODUCT REGISTRATION: Human iPS cells

TEST RESULTS:

# Tested	# Positives (Growth)	- Control
10	0	2 Negatives

TEST SUMMARY:

# Samples	Media Type	Volume (mL)	Incubation Temperature (° C)	Incubation Duration (Days)
10	TSB	40	20-25	14
10	FTG	40	30 - 35	14

REFERENCE: Processed according to LAB-003: Sterility Test Procedure

METHOD VALIDATION / PD #: 000053

TEST METHODOLOGY: USP - Direct Transfer

COMMENTS: NA

REVIEWED BY ______________________ DATE 15 Aug 17

Specific test results may not be indicative of the characteristics of any other samples from the same lot or similar lots. This test report shall not be reproduced, except in full, without prior written approval. Liability is limited to the costs of the tests.

Mycoplasma Detection Assay Report

Testing Performed by WiCell

Lot Release Testing

July 28, 2017

FORM SOP-QU-004.01
Version F Edition 02
Reported by: OG
Reviewed by: JB
BD Monolight 180

#	Sample Name	Reading A		A	Reading B		B	Ratio	Result	Comments/Suggestions
		RLU1	RLU2	Ave	RLU1	RLU2	Ave	B/A		
1	WA09-WB66446 12683	486	493	489.5	185	184	184.5	0.38	Negative	
2	Positive (+) Control	451	453	452	43705	43895	43800	96.90	Positive	
3	Negative (-) Control	803	817	810	89	88	88.5	0.11	Negative	

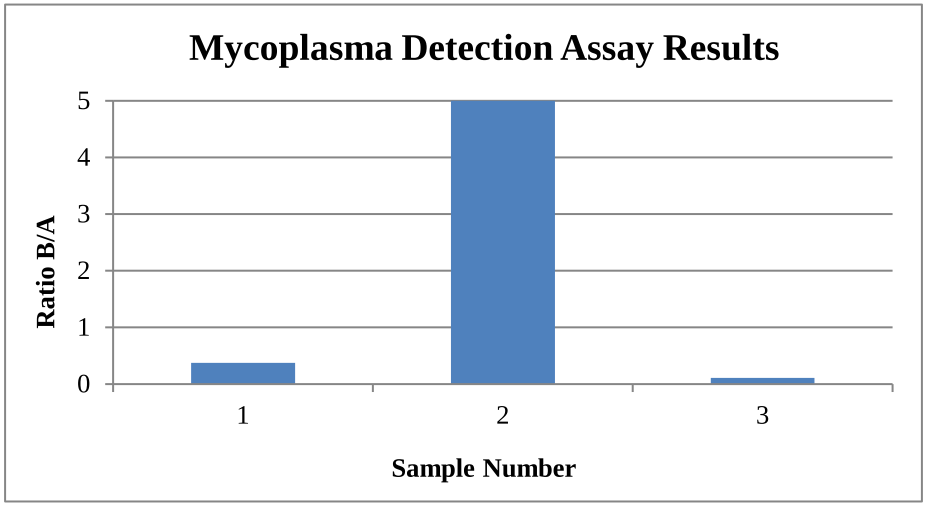

Supplement: H9 hESCs original unmodified cells [file mmc4.pdf]
