# Supplementary material for: Global and single-cell proteomics view of the co-evolution between neural progenitors and breast cancer cells in a co-culture model
Source: eBioMedicine. 2024 Sep 4;108:105325. doi: 10.1016/j.ebiom.2024.105325 (PMC11404160; doi:10.1016/j.ebiom.2024.105325)
Supplement: H9inGFPhESCs.pdf [file mmc5.pdf]

Product Information and Testing - Amended

Product Information

Product Name	H9inGFPhES
Alias	inGFPhES#3
Lot Number	H9inGFPhES-MCB-01
Depositor	University of Wisconsin – Laboratory of Dr. Su-Chun Zhang
Banked by	WiCell
Thaw Recommendation	Thaw 1 vial into 4 wells of a 6 well plate.
Culture Platform	Feeder Independent
	Medium: mTeSR1
	Matrix: Matrigel
Protocol	Feeder Independent Stem Cell Protocol Supplement: Culturing with Doxycycline
Passage Number	p37(5)<br><br>These cells were cultured for 36 passages prior to freeze, 5 of them in mTeSR1/Matrigel. WiCell adds +1 to the passage number at freeze so that the number on the vial best represents the overall passage number of the cells at thaw.
Date Vialed	09-October-2009
Vial Label	H9inGFPhES-MCB-01<br>p37(5) KR<br>09 OCT 09<br>SOPCC038A
Biosafety and Use Information	Appropriate biosafety precautions should be followed when working with these cells. The end user is responsible for ensuring that the cells are handled and stored in an appropriate manner. WiCell is not responsible for damages or injuries that may result from the use of these cells. Cells distributed by WiCell are intended for research purposes only and are not intended for use in humans.

Testing Performed by WiCell

Test Description	Test Provider	Test Method	Test Specification	Result
Post-Thaw Viable Cell Recovery	WiCell	SOP-CH-305	≥ 15 Undifferentiated Colonies, ≤ 30% Differentiation	Pass
Identity by STR	UW Molecular Diagnostics Laboratory	PowerPlex 1.2 System by Promega	Consistent with STR profile of deposited cell line	Pass
Sterility - Direct transfer method	Apptec	30744	No contamination detected	Pass
Mycoplasma	Bionique	M250	No contamination detected	Pass
Karyotype by G-banding	WiCell	SOP-CH-003	Normal karyotype	Pass

Date of Lot Release	Quality Assurance Approval
28-October-2010	8/17/2020<br>X HEB<br>HEB<br>Quality Assurance<br>Signed by: Bruner, Haley

© 2020 WiCell®
The material provided under this certificate has been subjected to the tests specified and the results and data described herein are accurate based on WiCell's reasonable knowledge and belief. Appropriate Biosafety Level practices and universal precautions should always be used with this material. For clarity, the foregoing is governed solely by WiCell's Terms and Conditions of Service, which can be found at http://www.wicell.org/privacyandterms.

UWHealth
University of Wisconsin
Hospital and Clinics

Short Tandem Repeat Analysis*

Sample Report: 0841-STR UW HLA#: 62056 Sample Date: 11/13/09
Received Date: 11/16/09

Requestor: WiCell Research Institute
Test Date: 11/19/09 File Name: 091120 Report Date: 11/21/09

Sample Name: (label on tube) 0841-STR **Description:** DNA Extracted by WiCell
223.09 ug/mL; 260/280 = 1.96

Locus	Repeat #	STR Genotype
D16S539	5, 8-15	12,13
D7S820	6-14	9,11
D13S317	7-15	9,9
D5S818	7-15	11,12
CSF1PO	6-15	11,11
TPOX	6-13	10,11
Amelogenin	NA	X,X
TH01	5-11	9.3,9.3
vWA	11, 13-21	17,17

Comments: Based on the DNA 0841-STR dated 11/13/09 and received on 11/16/09 from WI Cell, this sample (UW HLA# 62056) matches exactly the STR profile of the human stem cell line H9 comprising 12 allelic polymorphisms across the 8 STR loci analyzed. No STR polymorphisms other than those corresponding to the human H9 stem cell line were detected and the concentration of DNA required to achieve an acceptable STR genotype (signal/noise) was equivalent to that required for the standard procedure (~1 ng/amplification reaction) from human genomic DNA. These results suggest that the 0841-STR DNA sample submitted corresponds to the H9 stem cell line and it was not contaminated with any other human stem cells or a significant amount of mouse feeder layer cells. Sensitivity limits for detection of STR polymorphisms unique to either this or other human stem cell lines is ~5%.

HLA/Molecular Diagnostics Laboratory HLA/Molecular Diagnostics Laboratory

* Testing to assess engraftment following bone marrow transplantation was accomplished by analysis of human genetic polymorphisms at STR loci. This methodology has not yet been approved by the FDA and is for investigational use only.

File: Final STR Report

Test Facility:

This report is confidential. No part may be used for advertising or public announcement without written permission. Results apply only to the sample(s) tested.

WuXi AppTec

Report Number
821878.A01.R01

WiCell Research Institute

January 12. 2010
P.O. #:
Original Report Date:
11-12-09
AMENDED REPORT
❖ Amendment Summary
REISSUE # 1

Sample Information: hES Cells

1: UC01-DL-02, #0418
2: WIC-WA09-RB-003, #3178
3: SA01-DL-01, #4853
4: H9 (SYN-GFP)-MCB-01, #5205
5: H9 in GFPhES-MCB-01, #9519

Date Received: October 27, 2009
Date in Test: October 28, 2009
Date Completed: November 11, 2009

Test Information: Test Codes: 30744, 30744A
Immersion, USP / 21 CFR 610.12
Procedure #: BS210WCR.201

❖ A01 – Dated 12-16-09: Based on additional analysis, growth confirmed on sample # 3 (SCD). Amended pages 1 and 4.

QA Reviewer **Date** **Technical Reviewer** **Date**

Testing conducted in accordance with current Good Manufacturing Practices.

This report is confidential. No part may be used for advertising or public announcement without written permission. Results apply only to the sample(s) tested.

WuXi AppTec

WiCell Research Institute

January 12, 2010
P.O. #:
Original Report Date:
11-12-09
REISSUE # 1

Sample Information: hES Cells
5: H9 in GFPhES-MCB-01, #9519

Date Received: October 27, 2009
Date in Test: October 28, 2009
Date Completed: November 11, 2009

Test Information: Test Codes: 30744, 30744A
Immersion, USP / 21 CFR 610.12
Procedure #: BS210WCR.201

TEST PARAMETERS	PRODUCT	
**Approximate Volume Tested**	0.5 mL	0.5 mL
**Number Tested**	2	2
**Type of Media**	SCD	FTM
**Media Volume**	400 mL	400 mL
**Incubation Period**	14 Days	14 Days
**Incubation Temperature**	20 °C to 25 °C	30 °C to 35 °C
**RESULTS**	2 NEGATIVE	2 NEGATIVE

QA Reviewer **Date** **Technical Reviewer** **Date**

Testing conducted in accordance with current Good Manufacturing Practices.

Mycoplasma Testing Services
Safe Cells

BIONIQUE® TESTING LABORATORIES, INC.

APPENDIX BIONIQUE® TESTING LABORATORIES, INC.

Document ID #:	DCF9002E
Title:	QUALITY ASSURANCE REPORT - GMP
Effective Date:	05/21/09
Edition #:	01

QUALITY ASSURANCE REPORT – GMP

TEST PERFORMED	PROCEDURAL REFERENCE	TEST PERFORMED	PROCEDURAL REFERENCE
☒ M-250	SOP's 3008, 3011, 3013	☐ M-700	SOP's 3008, 3009, 3010
☐ M-300	SOP's 3008, 3014	☐ M-800	SOP's 3008, 3011, 3016
☐ M-350	SOP's 3008, 3014, 3015		

Bionique Sample ID #(s) 59387

This testing procedure was performed in compliance with the FDA's Current Good Manufacturing Practice (cGMP) standards (to the extent that the regulations pertain to the procedures performed) as specified in the Code of Federal Regulations, Title 21 Parts 210 and 211 [21 CFR 210 & 211]. All related records derived from the test procedures have been reviewed by the Quality Assurance Department. The individual's signature below verifies that the methods and procedures referenced above have been followed and that the Final Report accurately reflects the raw data generated during the course of the procedures. All records, including raw data and final reports are archived on site for a minimum of seven years.

The specified test's procedures determine the intervals at which samples are inspected. The medium used for testing must pass quality control mycoplasmal growth promotion testing and sterility testing. Traceability of all of the components used is assured and supporting documentation can be supplied upon request.

Quality Assurance Review Date: 12/10/09

Reviewed By: QA Assistant: [illegible]

NOTE:

1. Prior to receipt at Bionique® Testing Laboratories, Inc., the stability of the test article is the responsibility of the company submitting the sample. Bionique Testing Laboratories Inc. will assume responsibility for sample stability following receipt and prior to being placed on test.
2. This test is for the detection of microbiological growth and does not require statistical validation.

Document ID #:	DCF9002E
Title:	**QUALITY ASSURANCE REPORT - GMP**
Effective Date:	05/21/09
Edition #:	01

REFERENCES

Regulatory:

1. Department of Health and Human Services, Food and Drug Administration (USA) [FDA]. Code of Federal Regulations [CFR], Title 21 CFR Part 210, Current Good Manufacturing Practice in Manufacturing, Processing, Packing, or Holding of Drugs; General. FDA. Office of the Federal Register, National Archives and Records Department.
2. Department of Health and Human Services, Food and Drug Administration (USA) [FDA]. Code of Federal Regulations [CFR], Title 21 CFR Part 211, Current Good Manufacturing Practice for Finished Pharmaceuticals. FDA. Office of the Federal Register, National Archives and Records Department.
3. Department of Health and Human Services, Food and Drug Administration (USA) [FDA]. Points to Consider in the Characterization of Cell Lines Used to Produce Biologicals, Director, Center for Biologics Evaluation and Research, FDA. May, 1993. Docket No. 84N-0154.
4. Department of Health and Human Services, Food and Drug Administration (USA) [FDA]. Code of Federal Regulations [CFR], Title 21 CFR Part 610.30, General Biological Products Standards; Subpart D, Test for Mycoplasma. FDA. Office of the Federal Register, National Archives and Records Department.

General:

1. Barile MF, Kern J. Isolation of Mycoplasma arginini from commercial bovine sera and its implication in contaminated cell cultures. Proceedings of the Society for Experimental Biology and Medicine, Volume 138, Number 2, November 1971.
2. Chen, T.R. In situ detection of mycoplasma contamination in cell cultures by fluorescent Hoechst 33258 stain. Experimental Cell Research, 104: 255-262, 1977.
3. Carolyn K. Lincoln and Daniel J. Lundin. Mycoplasma Detection and Control. U. S. Fed. for Culture Collections Newsletter, Vol. 20, Number 4, 1990.
4. Fetal Bovine Serum; Proposed Guideline. National Committee For Clinical Laboratory Standards (NCCLS), Vol. 10, Number 6, 1990. (NCCLS publication M25-P).
5. McGarrity GJ, Sarama J, Vanaman V. Cell Culture Techniques. ASM News, Vol. 51, No. 4, 1985.
6. Tully JG, Razin S. Methods in Mycoplasmology, Volumes I and II. Academic Press, N.Y., 1983.
7. Barile MF, Razin S, Tully JG, Whitcomb RF. The Mycoplasmas, Volumes 1-4. Academic Press, N.Y., 1979.
8. http://www.bionique.com/ - Safe Cells Insights

Mycoplasma Testing Services Safe Cells

BIONIQUE TESTING LABORATORIES, INC.

APPENDIX IV

Document#:	DCF3013D
Edition#:	10
Effective Date:	07/15/2003
Title:	**M-250 FINAL REPORT SHEET**

M-250 FINAL REPORT

Direct Specimen Culture
Procedure 3008, 3011, 3013

TO: Wicell QA
WiCell Research Institute

BTL SAMPLE ID#: **59387** P.O.#: DATE REC'D: **11/12/2009**

TEST/CONTROL ARTICLE:

H9inGFPhES-MCB-01-G #0841

LOT#: **NA**

DIRECT CULTURE SET-UP (DAY 0) DATE: **11/12/2009**

INDICATOR CELL LINE (VERO) SEE DNA FLUOROCHROME RECORD SHEET

			DATE
THIOGLYCOLLATE BROTH	DAY 7	+ ⊖	**11/19/2009**
	DAY 28	+ ⊖	**12/10/2009**
BROTH-FORTIFIED COMMERCIAL			
**0.5** mL SAMPLE	DAY 7	+ ⊖	**11/19/2009**
**6.0** mL BROTH	DAY 28	+ ⊖	**12/10/2009**
BROTH-MODIFIED HAYFLICK			
**0.5** mL SAMPLE	DAY 7	+ ⊖	**11/19/2009**
**6.0** mL BROTH	DAY 28	+ ⊖	**12/10/2009**
BROTH-HEART INFUSION			
**0.5** mL SAMPLE	DAY 7	+ ⊖	**11/19/2009**
**6.0** mL BROTH	DAY 28	+ ⊖	**12/10/2009**

(See Reverse)

Document#:	DCF3013D
Edition#:	10
Effective Date:	07/15/2003
Title:	M-250 FINAL REPORT SHEET

SAMPLE ID#: **59387**		AEROBIC	MICROAEROPHILIC	DATE
AGAR PLATES-FORTIFIED COMMERCIAL	DAY 7	+ ⊖	+ ⊖	11/19/2009
	DAY 14	+ ⊖	+ ⊖	11/26/2009
	DAY 21	+ ⊖	+ ⊖	12/03/2009
AGAR PLATES-MODIFIED HAYFLICK	DAY 7	+ ⊖	+ ⊖	11/19/2009
	DAY 14	+ ⊖	+ ⊖	11/26/2009
	DAY 21	+ ⊖	+ ⊖	12/03/2009
AGAR PLATES-HEART INFUSION	DAY 7	+ ⊖	+ ⊖	11/19/2009
	DAY 14	+ ⊖	+ ⊖	11/26/2009
	DAY 21	+ ⊖	+ ⊖	12/03/2009

BROTH SUBCULTURES (DAY 7) DATE: **11/19/2009**

		AEROBIC	MICROAEROPHILIC	DATE
AGAR PLATES-FORTIFIED COMMERCIAL	DAY 7	+ ⊖	+ ⊖	11/26/2009
	DAY 14	+ ⊖	+ ⊖	12/03/2009
	DAY 21	+ ⊖	+ ⊖	12/10/2009
AGAR PLATES-MODIFIED HAYFLICK	DAY 7	+ ⊖	+ ⊖	11/26/2009
	DAY 14	+ ⊖	+ ⊖	12/03/2009
	DAY 21	+ ⊖	+ ⊖	12/10/2009
AGAR PLATES-HEART INFUSION	DAY 7	+ ⊖	+ ⊖	11/26/2009
	DAY 14	+ ⊖	+ ⊖	12/03/2009
	DAY 21	+ ⊖	+ ⊖	12/10/2009

RESULTS: No detectable mycoplasmal contamination

12/10/09
Date

M-250 Procedural Summary: The objective of this test is to ascertain whether or not detectable mycoplasmas are present in an in vitro cell culture sample, be it a primary culture, hybridoma, master seed stock or cell line. This procedure combines an indirect DNA staining approach to detect non-cultivable mycoplasmas with a direct culture methodology utilizing three different mycoplasmal media formulations. The indirect approach involves the inoculation of the sample into a mycoplasma-free VERO (ATCC) indicator cell line and performing a DNA fluorochrome assay after 72-120 hours of incubation. The direct culture aspect of the test utilizes three different mycoplasmal media including both broth and agar formulations. The sample is inoculated into each of the 3 broth formulations and also onto duplicate plates (0.1 mL/plate) for each of the 3 agar formulations. Subculture from broth to fresh agar plates is carried out after 7 days incubation. Agar plates are incubated aerobically and microaerophillically in order to detect any colony forming units morphologically indicative of mycoplasmal contamination. Issuance of the final report with signature of the Laboratory Director signifies that the required controls were performed concurrently with the test sample(s) as detailed in the referenced SOPs and that all test conditions have been found to meet the required acceptance criteria for a valid test, including the appropriate results for the positive and negative controls.

Mycoplasma Testing Services
Safe Cells

MYCOPLASMA
TESTING SERVICES

BIONIQUE TESTING LABORATORIES, INC

APPENDIX I
Document #: DCF3008A
Edition #: 06
Effective date: 9/17/2003
Title: DNA FLUOROCHROME ASSAY RESULTS

DNA-FLUOROCHROME ASSAY RESULTS

Procedures 3008, 3009, 3011

Sample ID # **59387** **M-250** Date Rec'd: **11/12/2009** P.O. #

Indicator Cells Inoculated: Date/Initials: 11/13/09 / JB

Fixation: Date/Initials: 11/17/09 / KG

Staining: Date/Initials: 11/17/09 / KG

TEST/CONTROL ARTICLE:

H9inGFPhES-MCB-01-G #0841

LOT# **NA**

Wicell QA
WiCell Research Institute

DNA FLUOROCHROME ASSAY RESULTS:

X **NEGATIVE:** A reaction with staining limited to the nuclear region, which indicates no mycoplasmal contamination.

___ **POSITIVE:** A significant amount of extranuclear staining which strongly suggests mycoplasmal contamination.

___ **INCONCLUSIVE:**

___ A significant amount of extranuclear staining consistent with low - level mycoplasmal contamination or nuclear degeneration.

___ A significant amount of extranuclear staining consistent with bacterial, fungal or other microbial contaminant or viral CPE. Morphology not consistent for mycoplasmal contamination.

COMMENTS:

Date: 11/17/09 Results Read by: KG Date of Review: 11/17/09 Reviewed by: [illegible]

WiCell Cytogenetics Report: *001435-102809*

WISC 0841

Report Date: November 04, 2009

Case Details:

Cell Line: *H9inGFPhES (0841)*

Passage #: *39(7)*

Date Completed: *11/4/2009*

Cell Line Gender: *Female*

Investigator: *WiCell Stem Cell Bank*

Specimen: *hESC on Matrigel*

Date of Sample: *10/28/2009*

Tests,Reason for: *WISC Testing*

Results: *46,XX*
Completed by , CLSp(CG), on 11/4/2009

Reviewed and interpreted by , PhD, FACMG, on 11/4/2009

Interpretation: *No clonal abnormalities were detected at the stated band level of resolution.*

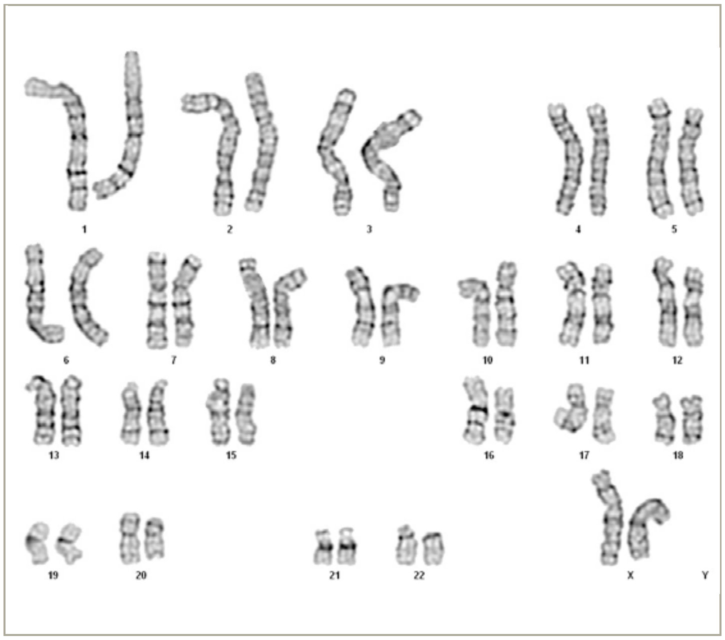

Cell: *S01-01*

Slide: *A-6*

Slide Type: *Karyotyping*

***# of Cells Counted:* 20**

***# of Cells Karyotyped:* 4**

***# of Cells Analyzed:* 8**

***Band Level:* 425-525**

Results Transmitted by Fax / Email / Post
Sent By:____________________________
QC Review By: ________________________

Date:____________________________
Sent To:__________________________
Results Recorded: _______________
