# Supplementary material for: Global and single-cell proteomics view of the co-evolution between neural progenitors and breast cancer cells in a co-culture model
Source: eBioMedicine. 2024 Sep 4;108:105325. doi: 10.1016/j.ebiom.2024.105325 (PMC11404160; doi:10.1016/j.ebiom.2024.105325)
Supplement: MDA-MB-231 HTB-26_70000792 [file mmc8.pdf]

CERTIFICATE OF ANALYSIS

ATCC® Number: HTB-26™
Lot Number: 70000792

Name: MDA-MB-231
Description: Breast Adenocarcinoma
Species: Human (*Homo sapiens*)
Volume/Ampule: Approximately 1 mL
Date Frozen: 22DEC2016
Recovery: A T-75 setup at a dilution of 1:15 reaches approximately 50% confluence in 2 days.
Product Format: Cells cryopreserved in the appropriate cryopreservation medium
Expiration Date: Not applicable
Storage Conditions: Vapor phase of liquid nitrogen

Test / Method	Specification	Result
**Ampule passage number**	Report results	33
**Population doubling level (PDL)**	Report results	Not applicable
**Total cells/ampule**<br>(Cell count using Trypan Blue stain method)	Report results	1.9×10^6 total cells/ampule
**Post-freeze viability**<br>(Cell count using Trypan Blue stain method)	≥ 50.0%	99.3%
**Growth properties**<br>(Visual observation method)	Adherent	Adherent
**Morphology**<br>(Visual observation method)	Epithelial-like*	Epithelial-like
**Test for mycoplasma contamination**<br>Hoechst DNA stain (indirect) method<br>Agar culture (direct) method<br>PCR-based assay	 None detected<br>None detected<br>None detected	 None detected<br>None detected<br>None detected
**Species determination: COI assay (interspecies)**	Human	Human

CERTIFICATE OF ANALYSIS

ATCC® Number: **HTB-26™**

Lot Number: **70000792**

**Species determination: STR analysis (intraspecies)**	**Human (Unique DNA Profile)**<br>TH01: 7, 9.3<br>D5S818: 12<br>D13S317: 13<br>D7S820: 8, 9<br>D16S539: 12<br>CSF1PO: 12, 13<br>Amelogenin: X<br>vWA: 15, 18<br>TPOX: 8, 9	**Human (Unique DNA Profile)**<br>TH01: 7, 9.3<br>D5S818: 12<br>D13S317: 13<br>D7S820: 8, 9<br>D16S539: 12<br>CSF1PO: 12, 13<br>Amelogenin: X<br>vWA: 15, 18<br>TPOX: 8, 9
**Sterility test (BacT/ALERT 3D)**<br>iAST bottle (aerobic) at 32°C<br>iNST bottle (anaerobic) at 32°C	 No growth<br>No growth	 No growth<br>No growth
**Human pathogenic virus testing**<br>(PCR-based assay for HIV, HepB, HPV, EBV, and CMV)	Report results	HIV – None detected<br>HepB – None detected<br>HPV – None detected<br>EBV – None detected<br>CMV – None detected

* Epithelial-like: Any adherent cells of a polygonal shape with clear, sharp boundaries between them.

Robbin L Smith

Digitally signed by Robbin L Smith
DN: cn=Robbin L Smith, o=ATCC, ou=Quality Assurance Specialist, email=rsmith@atcc.org, c=US
Date: 2017.03.10 14:19:49 -05'00'

Quality Assurance Specialist; Quality Assurance

ATCC hereby represents and warrants that the material provided under this certificate is pure and has been subjected to the tests and procedures specified and that the results described, along with any other data provided in this certificate, are true and correct to the best of the company's knowledge and belief. This certificate does not extend to the growth and/or passage of any living organism or cell line beyond what is supplied within the container received from ATCC.

This product is intended to be used for laboratory research use only. It is not intended for use in humans, animals, or for diagnostics. Appropriate Biosafety Level (BSL) practices should always be used with this material. Refer to the Product Information Sheet for instructions on the correct use of this product.

ATCC products may not be resold, modified for resale, used to provide commercial services, or to manufacture commercial products without prior written agreement from ATCC.

© 2014 American Type Culture Collection. The ATCC trademark and trade name are owned by the American Type Culture Collection.

ATCC
10801 University Boulevard
Manassas, VA 20110-2209 USA
www.atcc.org

800-638-6597 or 703-365-2700
Fax: 703-365-2750
E-mail: tech@atcc.org
or contact your local distributor
